# Supplementary material for: Brain-restricted mTOR inhibition with binary pharmacology
Source: Nature. 2022 Sep 14;609(7928):822–8. doi: 10.1038/s41586-022-05213-y (PMC9492542; doi:10.1038/s41586-022-05213-y)

nature portfolio

https://doi.org/10.1038/s41586-022-05213-y

Supplementary information

Brain-restricted mTOR inhibition with binary pharmacology

In the format provided by the authors and unedited

Supporting Information for

Brain-Restricted mTOR Inhibition with Binary Pharmacology

Ziyang Zhang[1], Qiwen Fan[2,3], Xujun Luo[2,3], Kevin Lou[1], William A. Weiss[2,3,4,5], Kevan M. Shokat[1,*]

[1] Department of Cellular and Molecular Pharmacology and Howard Hughes Medical Institute, University of California, San Francisco, California

[2] Helen Diller Family Comprehensive Cancer Center, San Francisco, California

[3] Department of Neurology, University of California, San Francisco, California

[4] Department of Pediatrics, University of California, San Francisco, California

[5] Department of Neurological Surgery, University of California, San Francisco, California

[*] Corresponding author. kevan.shokat@ucsf.edu (K.M.S.).

Table of Contents

Supplementary Figures 3

Supplementary Figure 1. Unprocessed immunoblot images.. 3

Supplementary Figure 2. Gating strategy for the cellular dye (TAMRA) retention assay. 3

Supplementary Tables 4

Supplementary Table 1. Kinase profiling results of FK-dasatinib (provided separately as a spreadsheet file). 4

Supplementary Table 2. List of antibodies.. 4

Supplementary Notes.. 5

Supplementary Note 1. Computational Modeling of RapaBlock's Action 5

Supplementary Note 2. Chemical Synthesis 6

Supplementary Note 3. ^{1}H NMR and ^{13}C NMR Spectra of RapaBlock.. 68

Supplementary Figures

Supplementary Figure 1. Unprocessed immunoblot images.

Supplementary Figure 2. Gating strategy for the cellular dye (TAMRA) retention assay. This gating strategy is used for data shown in Extended Data Figure 1c and 6b.

Figure 1d. Immunoblot analysis of mTOR signaling in MCF7 cells treated with DMSO, RapaLink-1, FK506, or a combination of RapaLink-1 and FK506.

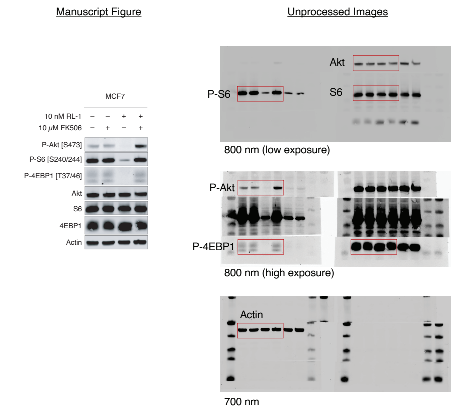

Note:
Lanes 5 and 6 contain samples that were treated with 10 nM MLN128 and 10 nM MLN128+10 μM RapaBlock. These two lanes were not central to the discussion and therefore were not included in Figure 1d.

Figure 3a and 3d. MCF7 cells were treated with a combination of RapaLink-1 and RapaBlock (**a**), or Rapamycin and RapaBlock (**d**) for 4 h, then phosphorylation of mTOR substrates were analyzed by immunoblotting. Results shown are representative of three independent experiments. GDPDH is a loading control.

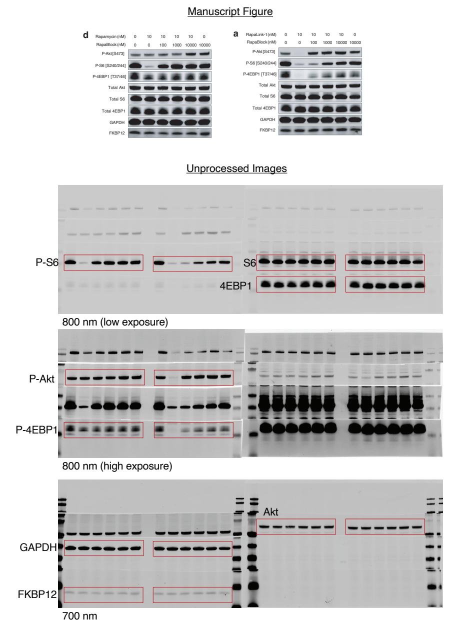

Figure 4a and Extended Data Figure 5. Biological replicates of the samples used in the quantification in Fig. 4a.

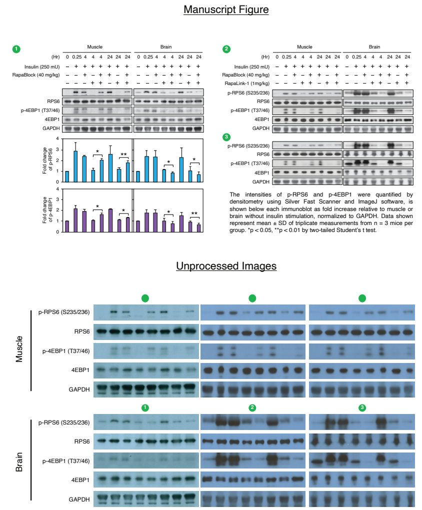

Extended Data Figure 3. Four candidate RapaBlock molecules were evaluated in cells (a) and *in vivo* (b).

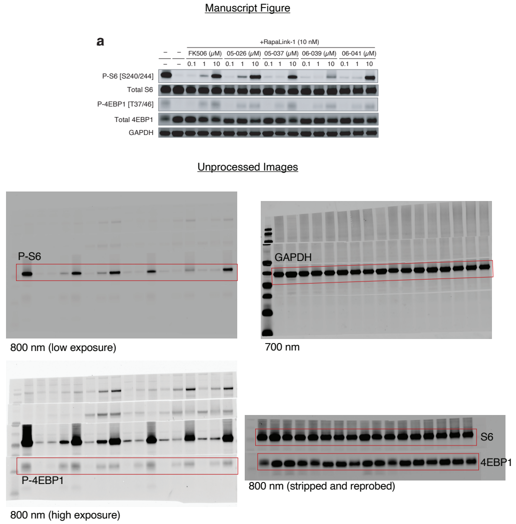

Extended Data Figure 4c. RapaBlock ethyl oxime is less potent at blocking RapaLink-1.

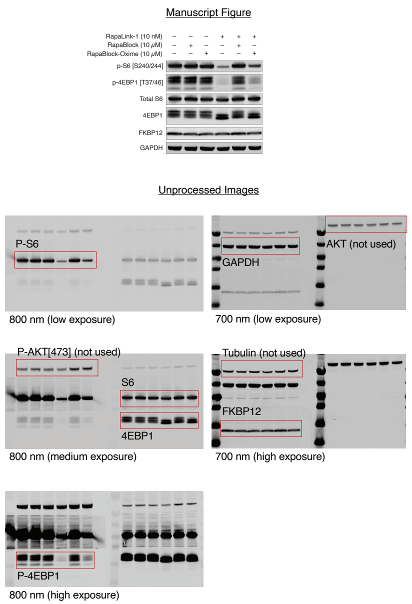

Extended Data Figure 3. Four candidate RapaBlock molecules were evaluated in cells (a) and *in vivo* (b).

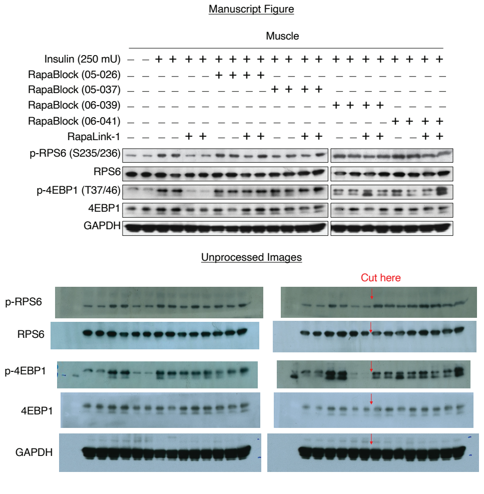

Extended Data Figure 3. Four candidate RapaBlock molecules were evaluated in cells (a) and *in vivo* (b).

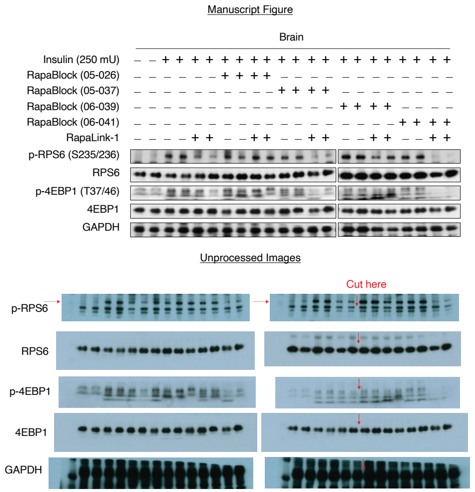

Extended Data Figure 7e. Jurkat cells were stimulated with anti-CD3 antibody (OKT3) in the presence of dasatinib, FK-dasatinib, and/or RapaBlock and analyzed by immunoblot. COX IV is a loading control.

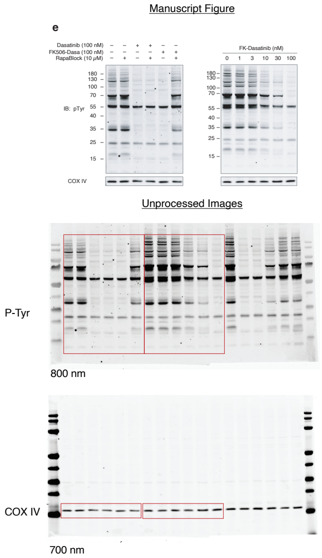

Note:
The 6 lanes on the right side of the gel contain samples for an unrelated experiment (notebook page ZZY-BIO07-050).

Extended Data Figure 7f. Jurkat cells were pulse-treated with Dasatinib (100 nM) or FK-Dasatinib (100 nM) for 1 h, then drugs were washed out and cells were incubated in drug-free media for various amounts of time, stimulated with anti-CD3 antibody (OKT3) for 5 min and analyzed by immunoblot. Results shown are representative of three independent experiments. COX-IV is a loading control.

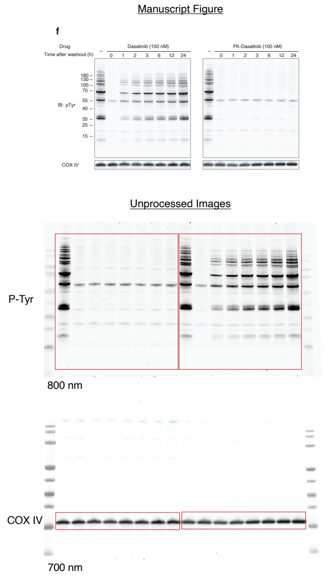

Extended Data Figure 8d. SK-BR3 cells were treated with Lapatinib, FK-Lapatinib, and/or RapaBlock for 1 h and analyzed by immunoblot. Results shown are representative of three independent experiments. GAPDH is a sample processing control.

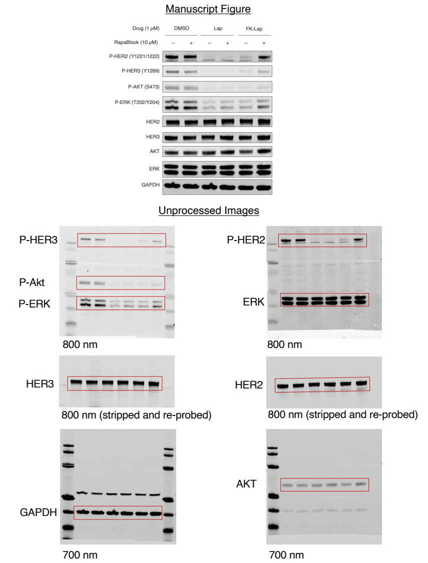

Note:
Because of the overlapping molecular weights of targets, two gels were run with the same samples and identical PAGE and transfer conditions. GAPDH is a sample processing control in this experiment.

Supplementary Figure 1. Unprocessed immunoblot images.

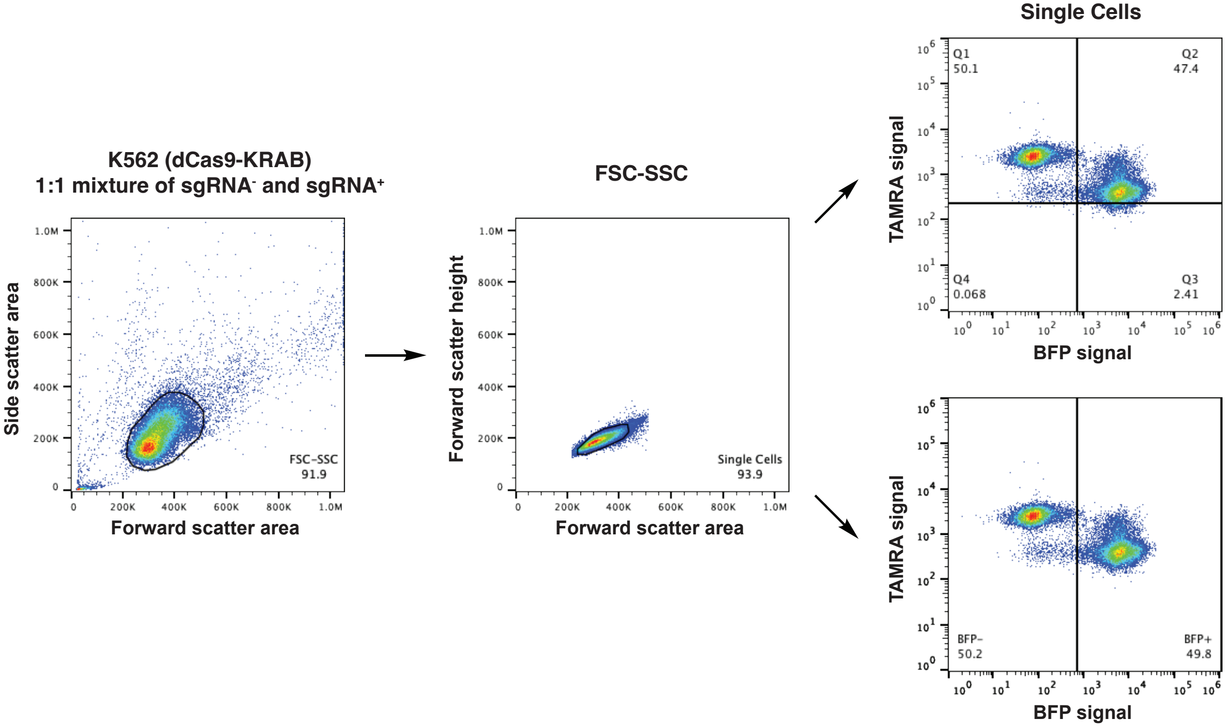

Supplementary Figure 2. Gating strategy for the cellular dye (TAMRA) retention assay. This gating strategy is used for data shown in Extended Data Figure 1c and 6b.

Supplementary Tables

Supplementary Table 1. Kinase profiling results of FK-dasatinib (provided separately as a spreadsheet file).

Supplementary Table 2. List of antibodies.

Target	Supplier	Identifier	Dilution
P-AKT [S473]	Cell Signaling Technology	4060	1:1000
AKT	Cell Signaling Technology	2920	1:1000
P-S6 [S240/S244]	Cell Signaling Technology	5364	1:2000
P-S6 [S235/S236]	Cell Signaling Technology	4858	1:2000
S6	Cell Signaling Technology	2217	1:1000
P-4EBP1 [T37/46]	Cell Signaling Technology	2855	1:1000
4EBP1	Cell Signaling Technology	9644	1:1000
FKBP12	abcam	58072	1:1000
Actin	Proteintech	60008-1-Ig	1:50000
GAPDH	Proteintech	60004-1-Ig	1:50000
P-Tyr (4G10)	EMD Millipore	05-321	1:1000
COX IV	Cell Signaling Technology	4850	1:1000
P-ERK [T202/Y204]	Cell Signaling Technology	9101	1:1000
Total ERK	Cell Signaling Technology	4695	1:1000
P-HER2 [Y1221/1222]	Cell Signaling Technology	2243	1:1000
P-HER3 [Y1289]	Cell Signaling Technology	2842	1:1000
HER2	Cell Signaling Technology	4290	1:1000
HER3	Cell Signaling Technology	4754	1:1000
Goat anti-mouse IgG-IRDye 800	LI-COR	926-32211	1:5000
Goat anti-mouse IgG-IRDye 800	LI-COR	926-68070	1:5000

Supplementary Notes

Supplementary Note 1. Computational Modeling of RapaBlock's Action

RapaBlock Modeling

In this discussion, we consider how a FKBP ligand blocks the inhibitory activity of Rapamycin and/or RapaLink-1 by competing for the cellular FKBP protein.

Model

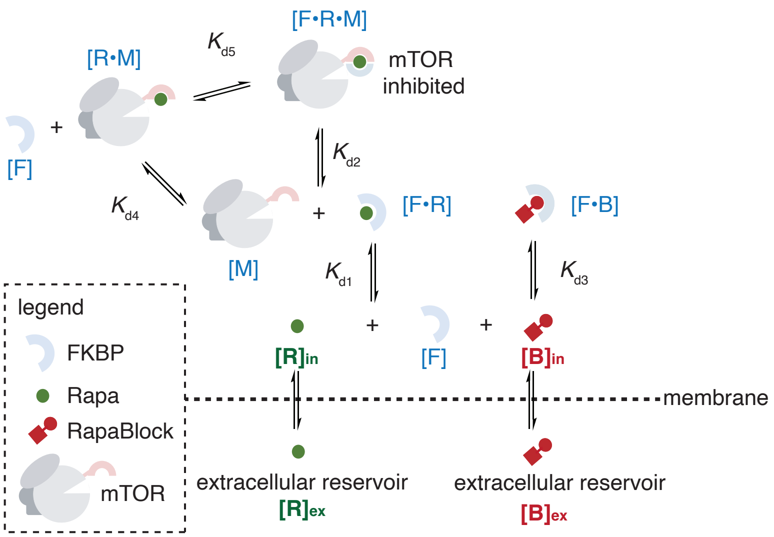

Assumption: extracelluar drugs constitute an infinite reagent reservoir
intracellular free drug concentration 10% that of extracellular because of permeability

We first consider the five equilibria in this system. The species are abbreviated as follows:

$[F]$ - free FKBP,
$[M]$ – free MTORC1,
$[R]_{in}$ – intracellular free Rapamycin or RapaLink-1,
$[B]_{in}$ – intrecellular free RapaBlock,
$[F \cdot R]$ – FKBP-Rapamycin or FKBP-RapaLink-1 complex,
$[F \cdot B]$ – FKBP-Rapamycin or RapaLink-1 complex,
$[R \cdot M]$ – Rapamycin-MTORC1 or RapaLink-1–MTORC1 complex without FKBP,
$[F \cdot R \cdot M]$ – FKBP-Rapamycin-MTORC1 or FKBP-RapaLink-1-MTORC1 ternary complex.

$$K_{d1} = \frac{[F][R]_{in}}{[F \cdot R]}$$

$$K_{d2} = \frac{[F \cdot R][M]}{[F \cdot R \cdot M]}$$

$$K_{d3} = \frac{[F][B]_{in}}{[F \cdot B]}$$

$$K_{d4} = \frac{[R][M]}{[R \cdot M]}$$

K_{d5} is constrained by K_{d1}, K_{d2} and K_{d4} and calculated as below

$$K_{d5} = \frac{K_{d1} \cdot K_{d2}}{K_{d4}} = \frac{[F][R \cdot M]}{[F \cdot R \cdot M]}$$

The mass balances of the system are

$$FKBP_{total} = [F] + [F \cdot R] + [F \cdot B] + [F \cdot R \cdot M]$$

$$MTORC1_{total} = [M] + [R \cdot M] + [F \cdot R \cdot M]$$

Substituting the complex concentrations with equilibria

$$FKBP_{total} = [F] + \frac{[F][R]_{in}}{K_{d1}} + \frac{[F][B]_{in}}{K_{d3}} + \frac{[F][R]_{in}[M]}{K_{d1} \cdot K_{d2}}$$

$$MTORC1_{total} = [M] + \frac{[R]_{in}[M]}{K_{d4}} + \frac{[F][R]_{in}[M]}{K_{d1} \cdot K_{d2}}$$

These can be transformed into a quadratic equation with a single variable $[F]$

$$\frac{[R]_{in}}{K_{d1} \cdot K_{d2}} \left(1 + \frac{[R]_{in}}{K_{d1}} + \frac{[B]_{in}}{K_{d3}}\right) [F]^2 + \left[\left(1 + \frac{[R]_{in}}{K_{d1}} + \frac{[B]_{in}}{K_{d3}}\right)\left(1 + \frac{[R]_{in}}{K_{d4}}\right) + \frac{[R]_{in}}{K_{d1} \cdot K_{d2}} (MTORC1_{total} - FKBP_{total})\right] [F] - \left(1 + \frac{[R]_{in}}{K_{d4}}\right) FKBP_{total} = 0 \quad (1)$$

The non-negative solution of $[F]$ from Eq (1) can be used to calculate inhibition% of MTORC1:

$$\begin{aligned} inhibition\% &= 1 - \frac{[M]}{MTORC1_{total}} \\ &= 1 - \frac{1}{1 + \frac{[R]_{in}}{K_{d4}} + \frac{[F][R]_{in}}{K_{d1} \cdot K_{d2}}} \end{aligned} \quad (2)$$

In our model, the intracellular total FKBP concentration $[FKBP]_{total}$ is estimated at 10 μM (Siekiera et al, PMID 1701173), the intracellular total mTOR concentration $[MTORC1]_{total}$ is estimated at 10 nM (Milo et al, PMID 19854939). Equilibrium constants K_{d2} and K_{d4} are from Banaszynski et al 2005; K_{d1} and K_{d3} are measured in-house in a single fluorescence polarization experiment for fair comparison.

We assume that the extracellular drugs constitute an infinite reagent reservoir, and the membrane permeability of both molecules are 10%. This gives a $[R]_{in}$ of $1\,nM$ and a $[B]_{in}$ of $0.1X\,nM$. We solve the quadratic equation (1) above and calculate the percentage of MTORC1 inhibition as a function of extracellular RapaBlock concentration X and plot the results in the figure below.

For Rapamycin, K_{d4} is estimated at 26 μM (Banaszynski et al, PMID 15796538).

Model – Rapamycin

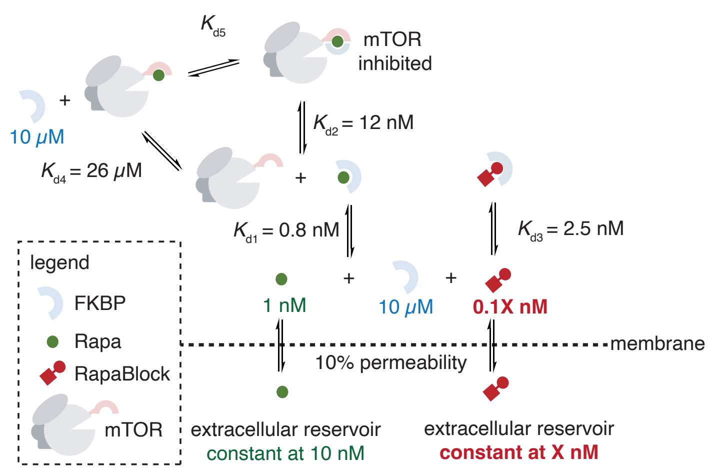

Assumption: extracelluar drugs constitute an infinite reagent reservoir intracellular free drug concentration 10% that of extracellular because of permeability

How much RapaBlock is needed to block Rapamycin?

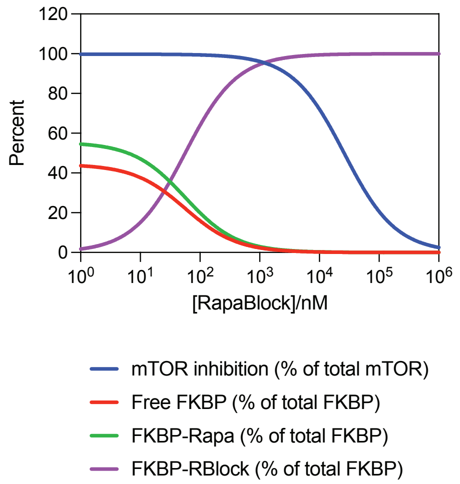

For RapaLink-1, K_{d4} is estimated at 5 nM which is the affinity of the TORKi portion for MTORC1.

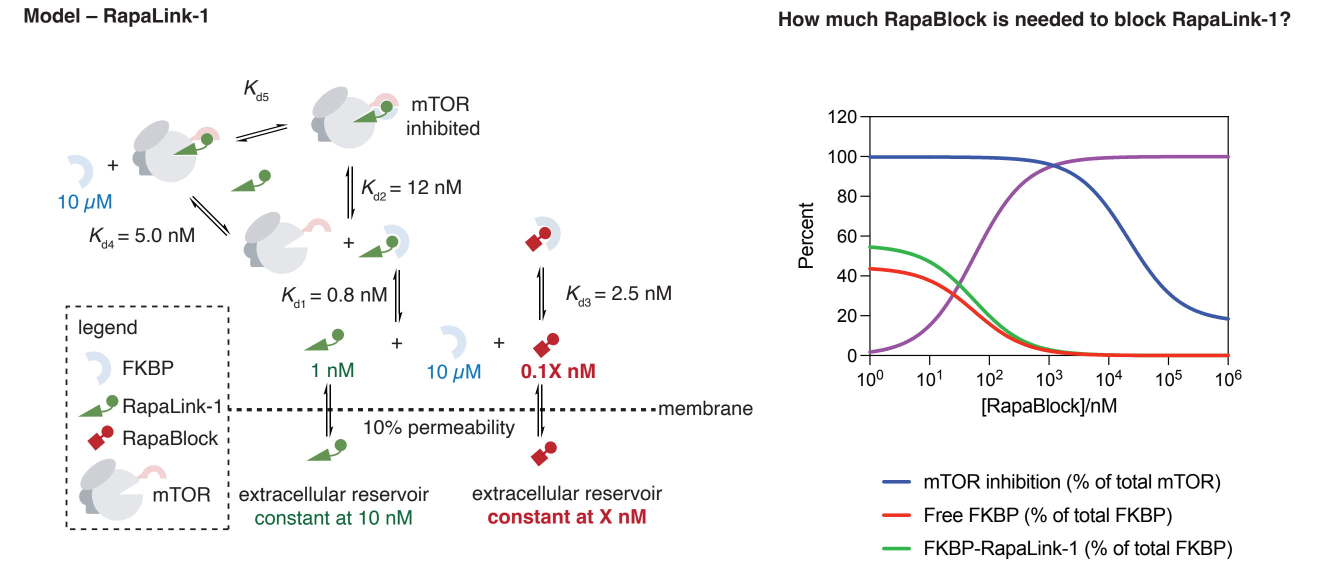

Assumption: extracelluar drugs constitute an infinite reagent reservoir
intracellular free drug concentration 10% that of extracellular because of permeability

It is also possible to model RapaBlock compounds with lower affinity to FKBP (varying K_{d3}):

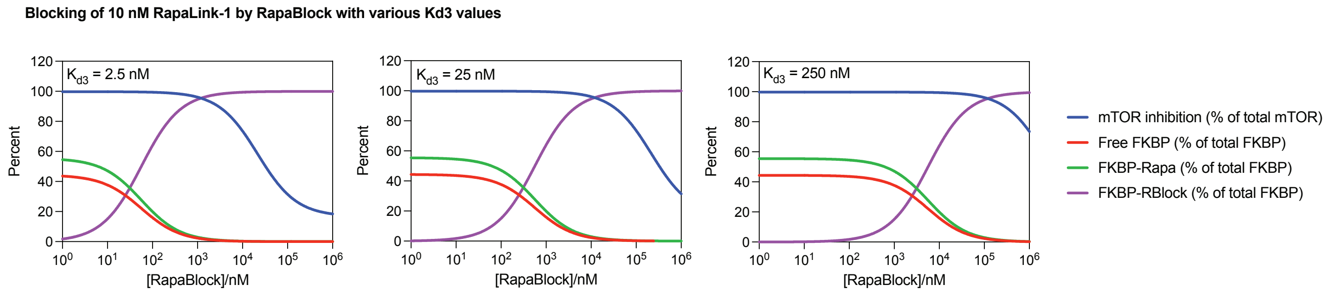

Here we observe that as we increase K_{d3} to 25 nM and 250 nM, RapaBlock becomes less effective at blocking RapaLink-1 ($EC_{50} \approx 200\ \mu M$ and $> 1\ mM$, respectively), consistent with our observation with the SLF-based ligands.

We note that the model has limitations:

• The binding constants used in the model may be subject to systematic and or non-systematic errors. In the figure for Rapamycin above, we used Kd2 and Kd4 values from Banaszynski et al. 2005, and Kd1 and Kd3 values measured in our lab in the same assay, as we believe this provides a fair comparison. For RapaLink-1, we used the same Kd2 value and an estimated Kd4 of 5 nM based on the ATP-competitive inhibitor portion of RapaLink-1.

• We made an approximation for the membrane permeability and efflux susceptibility of the compounds involved.

• We did not consider drug binding to proteins present in tissue culture medium/plasma (e.g. BSA/HSA).

• We assumed that there is an infinite extracellular pool of 10 nM Rapa. It may not be true as the FKBP sink effect could deplete extracellular Rapa.• The intracellular sink of immunophilins is complicated by the presence of mutliple FKBPs.There are 14 FKBPs which are known to bind Rapa and FK506 based immunophilins (Kozany et al. 2009, PMID: 19418507. DOI: 10.1002/cbic.200800806) and yet only a subset of these support Rapa mediated mTOR (FRB binding) inhibition.

Nevertheless, the model provides a reasonable estimation of the biochemical requirements for RapaBlock to block Rapamycin or RapaLink-1.

Supplementary Note 2. Chemical Synthesis

General Notes

General Experiment Procedure

All reactions were performed in oven-dried glassware fitted with rubber septa under a positive pressure of argon, unless otherwise noted. Air- and moisture-sensitive liquids were transferred via syringe. Solutions were concentrated by rotary evaporation at or below 40 °C. Analytical thin-layer chromatography (TLC) was performed using glass plates pre-coated with silica gel (0.25-mm, 60-Å pore size, 230−400 mesh, Merck KGA) impregnated with a fluorescent indicator (254 nm). TLC plates were visualized by exposure to ultraviolet light (UV), then were stained by submersion in a 10% solution of phosphomolybdic acid (PMA) in ethanol or an acidic ethanolic solution of *p*-anisaldehyde,[1] followed by brief heating on a hot plate. Flash column chromatography was performed with Teledyne ISCO CombiFlash EZ Prep chromatography system, employing pre-packed silica gel cartridges (Teledyne ISCO RediSep).

Solvents and Reagents

Anhydrous solvents were purchased from Acros Organics. Unless specified below, all chemical reagents were purchased from Sigma-Aldrich and AK Scientific. Commercial solvents and reagents were used as received. FK506 was purchased from LC Laboratories (Woburn, MA). SLF was purchased from Cayman Chemical and/or synthesized following the synthetic route reported by Holt el al. 3'-desamino-3'-hydroxy SLF was synthesized following the synthetic route reported by Holt et al. (Holt, D. A. et al. *J. Am. Chem. Soc.* **1993**, *115*, 9925–9938). Des(hydroxyethyl)dasatinib [N-(2-chloro-6-methylphenyl)-2-((2-methyl-6-(piperazin-1-yl)pyrimidin-4-yl)amino)thiazole-5-carboxamide] was purchased from 5A Chemicals. Lapatinib aldehyde [5-(4-((3-chloro-4-((3-fluorobenzyl)oxy)phenyl)amino)quinazolin-6-yl)furan-2-carbaldehyde] was purchased from AK Scientific. Desmethoxychloro erlotinib [6-(2-chloroethoxy)-N-(3-ethynylphenyl)-7-(2-methoxyethoxy)quinazolin-4-amine] was purchased from AstaTech. Desmethoxychloro gefitinib [N-(3-chloro-4-fluorophenyl)-6-(3-chloropropoxy)-7-methoxyquinazolin-4-amine] was purchased from AstaTech. Azido-PEG_8-Amine was purchased from BroadPharm, Inc. TAMRA 5-NHS ester was purchased from Click Chemistry Tools.

Instrumentation

Proton nuclear magnetic resonance (^{1}H NMR) spectra and carbon nuclear magnetic resonance (^{13}C NMR) spectra were recorded on Bruker AvanceIII HD 2-channel instrument (400 MHz/100 MHz) at 23 °C. Proton chemical shifts are expressed in parts per million (ppm, δ scale) and are referenced to residual protium in the NMR solvent

[1] This solution was prepared by sequential additions of concentrated sulfuric acid (5.0 mL), glacial acetic acid (1.5 mL) and *p*-anisaldehyde (3.7 mL) to absolute ethanol (135 mL) at 23 °C with efficient stirring.

($CHCl_3$: δ 7.26, D_2HCOD: δ 3.31). Carbon chemical shifts are expressed in parts per million (ppm, δ scale) and are referenced to the carbon resonance of the NMR solvent ($CDCl_3$: δ 77.0, CD_3OD: δ 49.0). Data are represented as follows: chemical shift, multiplicity (s = singlet, d = doublet, t = triplet, q = quartet, dd = doublet of doublets, dt = doublet of triplets, m = multiplet, br = broad, app = apparent), integration, and coupling constant (*J*) in Hertz (Hz). High-resolution mass spectra were obtained using a Waters Xevo G2-XS time-of-flight mass spectrometer. Unless otherwise specified, diastereomeric ratios of products are reported as (major diastereomer) : (sum of minor diastereomers).

Note on rotamers in ^1H and ^{13}C NMR data:

All of the FK506 analogs synthesized here exist as a mixture of two amide rotamers in $CDCl_3$ or CD_3OD (Mierke, D, F.; Schmieder, P.; Karuso, P.; Kessler, H. *Helv. Chim. Acta.* 1991, **74**, 1027–1047.). Due to extensive spectral overlap of the two, the coupling pattern of certain protons can be complicated even if they should display clear splitting patterns in theory. Sometimes, extensive spectral overlap prevents the identification of all peaks of the minor rotamer, and on occasion, of the major rotamer. In some cases, we could not accurately determine the proton counts by integration or the multiplicity of peaks due to such spectral overlaps. In this document, only ^1H NMR peaks of the major rotamer are reported in the best effort of resolving the peaks. ^{13}C NMR peaks of both rotamers are reported collectively.

Mini-workup

When a mini-workup (A/B) is indicated in the procedure, it was performed as follows: an aliquot (5 µL) of the reaction mixture was retrieved with a glass pipet and added to a plastic vial containing 0.2 mL organic solvent A and 0.2 mL aqueous solution B. The vial was shaken vigorously and allowed to stand until the two layers partitioned. The organic layer was then used for TLC or LC-MS analysis as specified in the procedure.

Monitoring Reaction Progress by LC-MS

When LC-MS analysis of the reaction mixture is indicated in the procedure, it was performed as follows. An aliquot (1 µL) of the reaction mixture (or the organic phase of a mini-workup mixture) was diluted with 100 µL 1:1 acetonitrile:water. 1 µL of the diluted solution was injected onto a Waters Acquity UPLC BEH C18 1.7 µm column and eluted with a linear gradient of 5–95% acetonitrile/water (+0.1% formic acid) over 3.0 min. Chromatograms were recorded with a UV detector set at 254 nm and a time-of-flight mass spectrometer (Waters Xevo G2-XS).

Numbering of Compounds

RapaBlock candidates, as shown in the main manuscript or extended data figures, are numbered based on the author's lab notebook pages in which these compounds were synthesized. Certain compounds may be referred to by more than one notebook page

number as they were prepared more than once. Synthetic intermediates that do not appear in the main manuscript or extended data figures are numbered in the order they appear in this Supplementary Information in the format of **Sx**, starting from **S1**.

Synthesis of RapaBlock

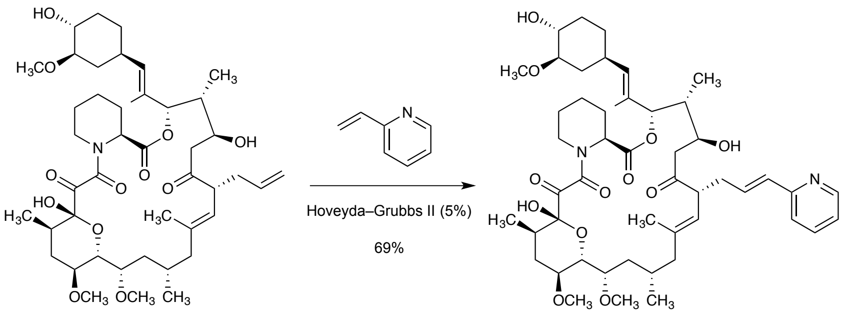

FK506 05-050

2-[(1E)-3-[(1R,9S,12S,13R,14S,17R,18E,21S,23S,24R,25S,27R)-1,14-dihydroxy-12-[(1E)-1-[(1R,3R,4R)-4-hydroxy-3-methoxycyclohexyl]prop-1-en-2-yl]-23,25-dimethoxy-13,19,21,27-tetramethyl-2,3,10,16-tetraoxo-11,28-dioxa-4-azatricyclo[22.3.1.0^{4,9}]octacos-18-en-17-yl]prop-1-en-1-yl]-1-methylpyridin-1-ium (**05-050**)

A 15-ml microwave vial was dried with gentle flame under vacuum. The vial was cooled to 23 °C, flushed with argon, then charged with FK506 (50 mg, 0.062 mmol), DCE (0.62 mL) and a magnetic stir bar. Argon was bubbled through the resulting solution via a 19-gauge needle for 1 min. 2-Vinylpyridine (6.7 µL, 0.062 mmol) was added via pipette, and Grubbs-Hoveyda 2nd Gen Catalyst (4.0 mg, 0.0062 mmol) was added in one portion as a solid. The mixture was stirred briefly (giving a bright green solution) before being loaded on a CEM DiscoverSP microwave reactor. Microwave reaction was performed at 100 °C for 30 min with 1 min pre-equilibration. After cooling to 23 °C, the reaction mixture was analyzed by TLC (100% ethyl acetate), which showed formation of an UV-active, more polar spot. The reaction mixture was directly loaded onto a 4-g RediSep (Teledyne ISCO) column. Elution with 100% ethyl acetate gave the product (**05-050**) as a yellow solid (38 mg, 69%).
3:2 mixture of rotamers.

^{1}H NMR (400 MHz, Chloroform-*d*) δ 8.55 – 8.47 (m, 1H), 7.66 – 7.56 (m, 1H), 7.24 (tt, *J* = 8.1, 1.1 Hz, 1H), 7.16 – 7.06 (m, 1H), 6.69 – 6.56 (m, 1H), 6.57 – 6.46 (m, 1H), 5.38 – 5.30 (m, 1H), 5.16 – 5.00 (m, 2H), 4.63 (d, *J* = 5.3 Hz, 1H), 4.44 (d, *J* = 14.1 Hz, 1H), 4.02 – 3.84 (m, 2H), 3.77 – 3.67 (m, 1H), 3.66 – 3.50 (m, 2H), 3.42 (s, 3H), 3.40 (s, 3H), 3.38 – 3.35 (m, 1H), 3.31 (s, 3H), 3.07 – 2.96 (m, 3H), 2.86 – 2.64 (m, 3H), 2.51 – 2.23 (m, 4H), 2.24 – 1.85 (m, 8H), 1.71 – 1.60 (m, 6H), 1.60 – 1.31 (m, 8H), 1.14 – 1.03 (m, 2H), 1.01 (d, *J* = 6.3 Hz, 3H), 0.94 (d, *J* = 6.7 Hz, 3H), 0.89 (d, *J* = 7.2 Hz, 3H). Number of protons: Expected: 72, Calculated from peak integration: 69.

^{13}C NMR (100 MHz, Chloroform-*d*) δ 212.69, 212.32, 196.06, 192.70, 168.90, 168.70, 165.78, 164.63, 155.65, 155.48, 149.32, 149.27, 140.05, 139.21, 136.43, 132.48, 132.34, 131.89, 131.84, 131.77, 131.66, 129.63, 129.55, 122.41, 122.26, 121.82, 121.76, 121.07, 121.04, 98.57, 97.04, 84.12, 77.83, 77.20, 76.47, 75.07, 73.59, 73.50, 73.47, 72.68, 72.16, 70.10, 68.96, 65.82, 57.51, 56.93, 56.61, 56.58, 56.54, 56.29, 56.09, 52.73, 52.67, 52.61, 48.52, 48.31, 44.09, 43.84, 43.24, 40.47, 39.98, 39.22, 35.32, 34.86, 34.82, 34.70, 34.57, 34.40, 34.12, 33.56, 32.76, 32.67, 32.52, 31.17, 30.58, 27.62, 26.17, 25.98, 24.50, 21.12, 20.83, 20.40, 19.37, 16.21, 15.97, 15.95, 15.82, 15.24, 14.22, 14.16, 9.75, 9.48.

HRMS (ESI): Calcd for $(C_{49}H_{72}N_2O_{12} + H)^+$: 881.5163, Found: 881.5207

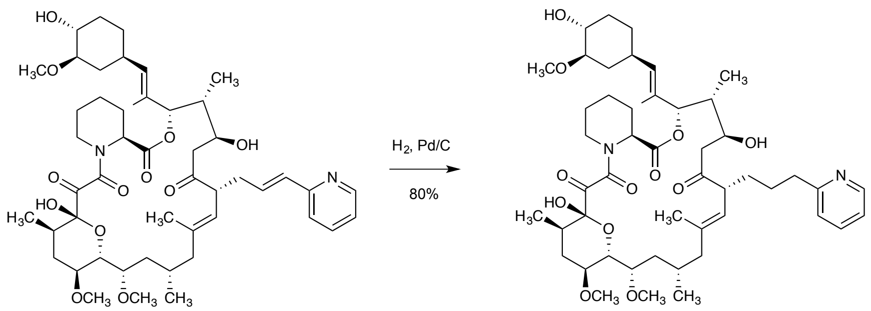

05-050 05-085

(1R,9S,12S,13R,14S,17R,18E,21S,23S,24R,25S,27R)-1,14-dihydroxy-12-[(1E)-1-[(1R,3R,4R)-4-hydroxy-3-methoxycyclohexyl]prop-1-en-2-yl]-23,25-dimethoxy-13,19,21,27-tetramethyl-17-[3-(pyridin-2-yl)propyl]-11,28-dioxa-4-azatricyclo[22.3.1.0^{4,9}]octacos-18-ene-2,3,10,16-tetrone (**05-085**)

A 20-mL vial was charged with **05-050** (20 mg, 0.020 mmol), Ethyl acetate (1.0 mL) and Palladium on carbon (10 wt%, 2.4 mg). The vial was briefly purged with argon, and then fitted with a rubber septum. Hydrogen was bubbled through the solution via a 19-gauge needle for 5 min, then the mixture was stirred under hydrogen atmosphere at 23 ºC. In a total of 3 h, LC-MS showed full conversion to the desired m/z. The reaction mixture was filtered through a pad of Celite, and the filter cake was rinsed with ethyl acetate (5 mL). The combined filtrate was concentrated to afford the product (**05-085**) as a pale-yellow foam (20 mg, 99%). 3:2 mixture of rotamers.

^1H NMR (400 MHz, Chloroform-d) δ 8.56 – 8.43 (m, 2H), 7.61 (tt, J = 7.6, 2.3 Hz, 2H), 7.21 – 7.07 (m, 4H), 5.37 (s, 1H), 5.28 – 5.15 (m, 1H), 5.15 – 4.96 (m, 2H), 4.62 (d, J = 5.0 Hz, 1H), 4.45 (d, J = 13.5 Hz, 1H), 4.00 – 3.86 (m, 1H), 3.74 (d, J = 9.8 Hz, 1H), 3.67 – 3.55 (m, 1H), 3.52 – 3.25 (m, 3H), 3.43 (s, 3H), 3.41 (s, 3H), 3.32 (s, 3H), 3.09 – 2.95 (m, 1H), 2.92 – 2.75 (m, 2H), 2.73 – 2.64 (m, 1H), 2.43 – 2.11 (m, 3H), 2.11 – 1.84 (m, 4H), 1.84 – 1.70 (m, 4H), 1.70 – 1.57 (m, 6H), 1.57 – 1.33 (m, 8H), 1.15 – 1.05 (m, 2H),

1.03 (d, J = 6.5 Hz, 3H), 0.96 (d, J = 6.1 Hz, 3H), 0.87 (d, J = 7.2 Hz, 3H). Number of protons: Expected: 74, Calculated from peak integration: 71.

^{13}C NMR (100 MHz, Chloroform-d) δ 213.96, 213.18, 196.35, 192.17, 168.95, 168.22, 166.01, 164.96, 161.79, 161.65, 149.16, 148.94, 139.64, 138.28, 136.48, 136.36, 132.46, 131.80, 129.57, 129.26, 123.24, 122.84, 122.71, 121.04, 98.59, 97.33, 84.13, 77.80, 77.20, 76.68, 76.50, 75.15, 73.68, 73.51, 72.64, 72.18, 70.25, 68.85, 57.54, 56.88, 56.66, 56.59, 56.53, 56.30, 56.06, 52.98, 52.66, 52.09, 48.23, 42.86, 40.41, 39.77, 39.14, 37.97, 37.53, 34.84, 34.72, 33.60, 32.84, 32.64, 31.18, 30.75, 30.61, 29.91, 27.44, 27.33, 27.20, 26.10, 25.94, 24.47, 21.18, 20.83, 20.55, 19.45, 16.35, 16.16, 15.97, 15.65, 14.27, 14.21, 9.74, 9.49.

HRMS (ESI): Calcd for $(C_{49}H_{74}N_2O_{12} + H)^+$: 883.5320, Found: 883.5332

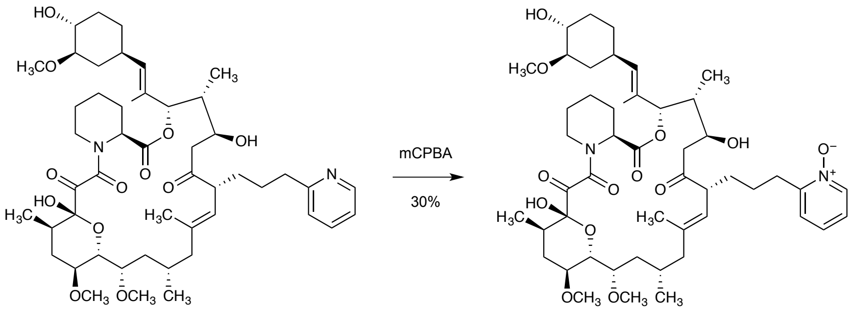

05-085

RapaBlock (05-086/06-041)

3-{3-[(1R,9S,12S,13R,14S,17R,18E,21S,23S,24R,25S,27R)-1,14-dihydroxy-12-[(1E)-1-[(1R,3R,4R)-4-hydroxy-3-methoxycyclohexyl]prop-1-en-2-yl]-23,25-dimethoxy-13,19,21,27-tetramethyl-2,3,10,16-tetraoxo-11,28-dioxa-4-azatricyclo[22.3.1.04,9]octacos-18-en-17-yl]propyl}pyridin-1-ium-1-olate (**RapaBlock, 05-086, 06-041**)

m-CPBA (6.6 mg, 0.025 mmol) was added as a 10% solution (w/v) in dichloromethane (66 µL) to a solution of **05-085** (22 mg, 0.025 mmol) in dichloromethane at 0 ºC. The reaction progress was monitored by LC-MS. In a total of 6 h, LC-MS showed full conversion to the desired m/z. The reaction mixture was directly concentrated under reduced pressure. The residue was diluted with 50% acetonitrile–water to a volume of 3.0 mL, and the solution was filtered through a 0.45 µM PTFE syringe filter. The filtrate was purified by reverse-phase HPLC (Waters XBridge C18 column 5 µm particle size 30 x 250 mm, 50–95% acetonitrile–water + 0.1% formic acid, 40 min, 20 mL/min) to afford the product as a white solid (6.6 mg, 30%). 3:2 mixture of rotamers.

^1H NMR (400 MHz, $CDCl_3$) δ 8.24 (d, *J* = 6.4 Hz, 1H), 7.27 – 7.20 (m, 2H), 7.19 – 7.12 (m, 1H), 5.34 (d, *J* = 2.3 Hz, 1H), 5.13 (d, *J* = 9.8 Hz, 1H), 5.10 – 5.04 (m, 1H), 5.00 (d, *J* = 8.3 Hz, 1H), 4.82 – 4.76 (m, 1H), 4.60 – 4.54 (m, 1H), 4.42 (d, *J* = 13.6 Hz, 1H), 4.01 – 3.90 (m, 1H), 3.87 (dd, *J* = 9.6, 2.5 Hz, 0H), 3.75 – 3.67 (m, 2H), 3.64 – 3.53 (m, 2H), 3.49 – 3.42 (m, 3H), 3.41 (s, 3H), 3.39 (s, 3H), 3.30 (s, 3H), 3.06 – 2.97 (m, 2H), 2.93 (app t, *J* = 7.7 Hz, 2H), 2.86 – 2.74 (m, 1H), 2.44 – 2.24 (m, 3H), 2.24 – 1.95 (m, 6H), 1.95 – 1.84 (m, 2H), 1.81 – 1.67 (m, 4H), 1.65 (s, 3H), 1.56 (s, 3H), 1.54 – 1.27 (m, 6H), 1.11 – 1.02 (m, 2H), 1.00 (d, *J* = 6.5 Hz, 3H), 0.93 (d, *J* = 5.8 Hz, 3H), 0.86 (d, *J* = 7.2 Hz, 3H). Number of protons: Expected: 74, Calculated from peak integration: 71.

^{13}C NMR (100 MHz, Chloroform-*d*) δ 213.54, 212.84, 196.34, 192.92, 168.99, 168.74, 165.82, 164.97, 152.29, 152.11, 139.85, 139.75, 139.70, 138.52, 132.56, 131.94, 129.57, 129.40, 126.31, 125.97, 125.66, 125.54, 123.55, 123.03, 122.82, 98.59, 97.27, 84.16, 77.76, 77.27, 76.47, 74.82, 73.70, 73.61, 73.54, 73.50, 72.69, 72.21, 70.30, 69.24, 65.85, 57.53, 56.93, 56.69, 56.63, 56.60, 56.57, 56.33, 56.14, 52.77, 52.68, 52.34, 48.37, 44.01, 43.88, 43.21, 40.56, 39.98, 39.17, 35.31, 34.91, 34.87, 34.77, 34.72, 33.60, 32.87, 32.61, 31.26, 30.65, 30.58, 30.32, 27.48, 26.24, 26.15, 26.04, 24.51, 23.77, 23.65, 21.20, 20.93, 20.53, 19.46, 16.26, 16.23, 16.00, 15.85, 15.28, 14.29, 14.27, 9.84, 9.60.

HRMS (ESI): Calcd for $(C_{49}H_{74}N_2O_{13} + H – H_2O)^+$: 881.5164, Found: 881.5150

Synthesis of Other RapaBlock Candidate Compounds

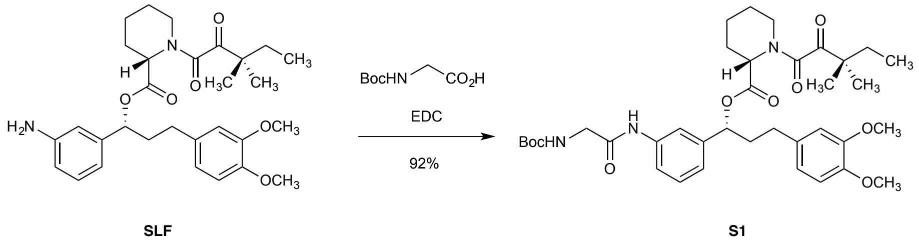

(R)-1-(3-(2-((tert-butoxycarbonyl)amino)acetamido)phenyl)-3-(3,4-dimethoxyphenyl)propyl (S)-1-(3,3-dimethyl-2-oxopentanoyl)piperidine-2-carboxylate (**S1**)

EDC (548 mg, 2.86 mmol) was added in one portion to a stirred solution of SLF (1.00 g, 1.91 mmol) and Boc-Gly-OH (500 mg, 2.86 mmol) in dichloromethane (9.5 mL) at 0 ºC. The resulting mixture was allowed to warm to 23 ºC over 30 min, then was kept stirred at 23 ºC. After a total of 5 h, TLC analysis (50% ethyl acetate–hexanes) showed that the reaction was complete. The reaction mixture was directly concentrated under reduced pressure, and the residue was purified by column chromatography (20–50% ethyl acetate–hexanes, 40-g CombiFlash column) to afford the product (**S1**) as a white foam (1.20 g, 92%).

The product exists as a 6:1 mixture of amide rotamers.

^{1}H NMR (400 MHz, Chloroform-*d*) δ 8.33 (s, 1H), 7.62 (d, *J* = 8.1 Hz, 1H), 7.51 – 7.45 (m, 1H), 7.30 (t, *J* = 7.9 Hz, 1H), 7.03 (d, *J* = 7.8 Hz, 1H), 6.77 (d, *J* = 8.7 Hz, 1H), 6.73 – 6.63 (m, 2H), 5.80 (dd, *J* = 7.7, 5.6 Hz, 1H), 5.34 (d, *J* = 5.5 Hz, 1H), 5.27 (s, 1H), 3.98 – 3.89 (m, 2H), 3.86 (s, 3H), 3.84 (s, 3H), 3.34 (d, *J* = 13.7 Hz, 1H), 3.07 (td, *J* = 12.9, 3.0 Hz, 1H), 2.64 – 2.49 (m, 2H), 2.35 (d, *J* = 13.6 Hz, 1H), 2.28 – 2.15 (m, 1H), 2.12 – 2.02 (m, 1H), 1.78 – 1.58 (m, 5H), 1.47 (s, 9H), 1.46 – 1.34 (m, 2H), 1.24 (s, 3H), 1.24 (s, 3H), 0.91 (t, *J* = 7.5 Hz, 3H). Number of protons: Expected: 51, Calculated from peak integration: 51.
HRMS (ESI): Calcd for $(C_{37}H_{51}N_3O_9 + H)^+$: 682.3704, Found: 682.3699.

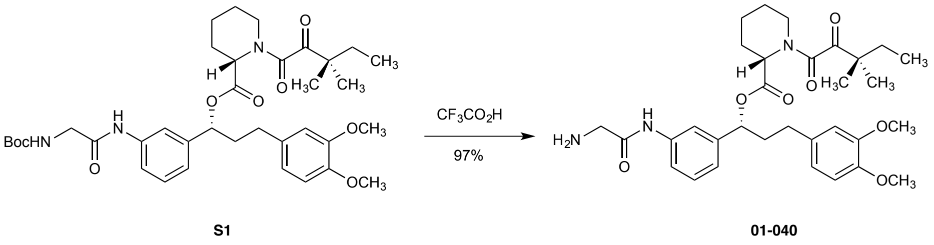

(*R*)-1-(3-(2-aminoacetamido)phenyl)-3-(3,4-dimethoxyphenyl)propyl (*S*)-1-(3,3-dimethyl-2-oxopentanoyl)piperidine-2-carboxylate (**01-040**)

A solution of **S1** (1.20 g, 1.76 mmol) in dichloromethane (8.4 mL) was cooled to 0 ºC, then trifluoroacetic acid (8.4 mL) was added dropwise. The resulting yellow solution was stirred at 0 ºC until TLC analysis (50% ethyl acetate–hexanes, mini-workup with ether/aqueous sodium bicarbonate solution) showed full consumption of the starting material. The reaction solution was concentrated in vacuo, and the residue was partitioned between dichloromethane (10 mL) and saturated sodium bicarbonate solution (10 mL). The layers were separated, and the aqueous layer was extracted with dichlomethane (2 x 10 mL). The combined organic layers were dried over sodium sulfate, and the dried solution was concentrated in vacuo to afford the product (**01-040**) as a white foam (997 mg, 97%).

6:1 mixture of rotamers.
^{1}H NMR (400 MHz, Chloroform-*d*) δ 7.30 – 7.22 (m, 1H), 7.00 – 6.88 (m, 2H), 6.88 – 6.80 (m, 2H), 6.80 – 6.74 (m, 1H), 6.70 – 6.65 (m, 2H), 5.77 (dd, *J* = 7.8, 5.9 Hz, 1H), 5.31 (d, *J* = 5.5 Hz, 1H), 4.53 (s, 2H), 3.86 (s, 3H), 3.85 (s, 3H), 3.36 (d, *J* = 13.0 Hz, 1H), 3.14 (td, *J* = 13.1, 3.2 Hz, 1H), 2.66 – 2.45 (m, 2H), 2.36 (d, *J* = 13.7 Hz, 1H), 2.31 – 2.15 (m, 1H), 2.04 (s, 1H), 1.82 – 1.58 (m, 5H), 1.42 – 1.29 (m, 2H), 1.23 (s, 3H), 1.21 (s, 3H), 0.89 (t, *J* = 7.5 Hz, 3H). Number of protons: Expected: 43, Calculated from peak integration: 41.

HRMS (ESI): Calcd for $(C_{32}H_{43}N_3O_7 + H)^+$: 582.3179, Found: 582.3176.

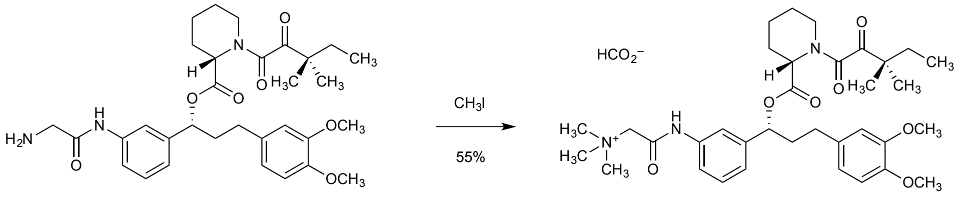

01-040

01-025

2-((3-((*R*)-3-(3,4-dimethoxyphenyl)-1-(((*S*)-1-(3,3-dimethyl-2-oxopentanoyl)piperidine-2-carbonyl)oxy)propyl)phenyl)amino)-*N*,*N*,*N*-trimethyl-2-oxoethan-1-aminium (**01-025)**

Sodium bicarbonate (36.5 mg, 0.430 mmol) and iodomethane (107 µL, 1.72mmol) were added to a solution of 01-040 (50 mg, 0.090 mmol) in THF (0.50 mL) at 23 ºC. The resulting suspension was stirred at 23 ºC, and the reaction progress was monitored by LC-MS. After 24 h, the bulk of the solvent was removed by rotary evaporation. The residue was diluted with 50% acetonitrile–water to a volume of 4.7 mL, and the solution was purified by reverse-phase HPLC (Waters XBridge C18 column 5 µm particle size 30 x 250 mm, 5–95% acetonitrile–water + 0.1% formic acid, 40 min, 20 mL/min) to afford the product (**01-025**) as a white solid (29.5 mg, 55%).

6:1 mixture of rotamers.
^{1}H NMR (400 MHz, Methanol-d_4) δ 8.35 (s, 1H), 7.81 – 7.64 (m, 1H), 7.51 – 7.43 (m, 1H), 7.43 – 7.33 (m, 1H), 7.18 (app t, *J* = 8.1 Hz, 1H), 6.92 – 6.85 (m, 1H), 6.85 – 6.79 (m, 1H), 6.79 – 6.70 (m, 1H), 5.83 – 5.70 (m, 1H), 5.29 – 5.22 (m, 1H), 4.31 (s, 2H), 3.83 (s, 3H), 3.82 (s, 3H), 3.41 (s, 10H), 3.30 – 3.19 (m, 1H), 2.75 – 2.55 (m, 2H), 2.37 (d, *J* = 13.6 Hz, 1H), 2.34 – 2.19 (m, 1H), 2.17 – 2.02 (m, 1H), 1.86 – 1.58 (m, 5H), 1.58 – 1.29 (m, 2H), 1.25 (s, 3H), 1.23 (s, 3H), 0.90 (t, *J* = 7.4 Hz, 3H). Number of protons: Expected: 51, Calculated from peak integration: 50.

HRMS (ESI): Calcd for $(C_{35}H_{50}N_3O_7)^+$: 624.3642, Found: 624.3636.

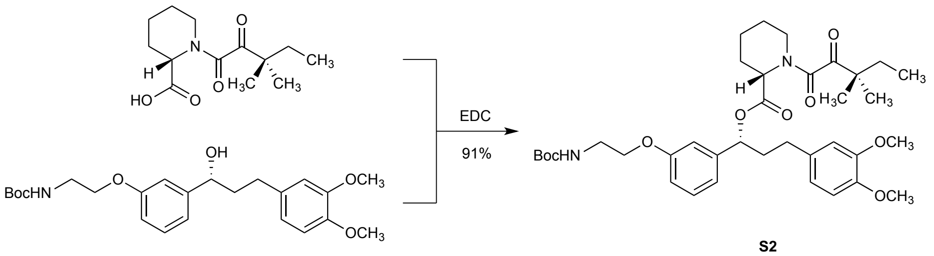

S2

(*R*)-1-(3-(2-((*tert*-butoxycarbonyl)amino)ethoxy)phenyl)-3-(3,4-dimethoxyphenyl)propyl (*S*)-1-(3,3-dimethyl-2-oxopentanoyl)piperidine-2-carboxylate (**S2**)

tert-butyl *N*-[2-[3-[(1*R*)-3-(3,4-dimethoxyphenyl)-1-hydroxy-propyl]phenoxy]ethyl]carbamate[2] (65 mg, 0.15 mmol) and (2*S*)-1-(3,3-dimethyl-2-oxo-pentanoyl)piperidine-2-carboxylic acid[3] (42.3 mg, 0.17 mmol) were dissolved in dry dichloromethane (3.0 mL). The resulting solution was cooled to 0 ºC, then EDC (43.5 mg, 0.23 mmol) was added in one portion as a solid. The ice bath was removed, and the reaction mixture was allowed to warmed to 23 ºC. In 4 h, TLC analysis (50% ethyl acetate–hexanes) showed that the reaction was complete. The reaction mixture was directly concentrated, and the residue was purified by column chromatography (4-g CombiFlash column, 10–50% ethyl acetate–hexanes) to afford the product (**S2**) as a colorless film (92 mg, 91%).

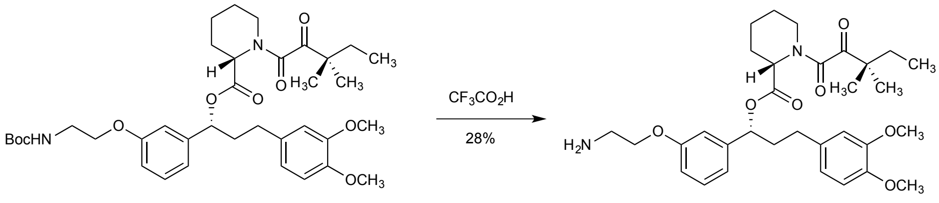

S2 01-038

(*R*)-1-(3-(2-aminoethoxy)phenyl)-3-(3,4-dimethoxyphenyl)propyl (*S*)-1-(3,3-dimethyl-2-oxopentanoyl)piperidine-2-carboxylate (**01-038**)

50% trifluoroacetic acid–dichloromethane (1 mL) was added to **S2** (95 mg, 0.14 mmol) in a 20-mL scintillation vial at 0 ºC. The resulting solution was warmed to 23 ºC over 5 min. TLC analysis (mini-workup with ether/sodium bicarbonate, 50% ethyl acetate–hexanes) showed complete consumption of the starting material. The product has an Rf of 0 in this solvent system. The reaction mixture was concentrated under reduced pressure. The residue was partitioned between dichloromethane (5 mL) and saturated sodium bicarbonate solution (5 mL). The layers were separated, and the aqueous layer

[2] Holt, D. A. et al. *J. Am. Chem. Soc.* **1993**, *115*, 9925–9938.
[3] Holt, D. A. et al. *J. Am. Chem. Soc.* **1993**, *115*, 9925–9938.

was extracted with dichloromethane (2 x 5 mL). The combined organic layers were dried over sodium sulfate, and the dried solution was concentrated. The residue was purified by column chromatography (3–5% methanol–dichloromethane + 0.3–0.5% saturated aqueous ammonium hydroxide solution, 4-g CombiFlash column) to afford the product (**01-038**) as a colorless film (23 mg, 28%).

6:1 mixture of rotamers.
^{1}H NMR (400 MHz, Chloroform-*d*) δ 7.29 – 7.22 (m, 1H), 6.95 – 6.88 (m, 2H), 6.85 (dd, *J* = 8.2, 2.2 Hz, 1H), 6.78 (d, *J* = 8.6 Hz, 1H), 6.72 – 6.65 (m, 2H), 5.77 (dd, *J* = 7.9, 5.8 Hz, 1H), 5.35 – 5.28 (m, 1H), 4.05 – 3.94 (m, 2H), 3.86 (s, 3H), 3.85 (s, 3H), 3.35 (d, *J* = 13.6 Hz, 1H), 3.20 – 3.06 (m, 3H), 2.67 – 2.43 (m, 2H), 2.42 – 2.32 (m, 1H), 2.31 – 2.17 (m, 1H), 2.13 – 1.97 (m, 1H), 1.76 – 1.57 (m, 5H), 1.57 – 1.27 (m, 2H), 1.22 (s, 3H), 1.20 (s, 3H), 0.88 (t, *J* = 7.5 Hz, 3H). Number of protons: Expected: 44, Calculated from peak integration: 42.

HRMS (ESI): Calcd for $(C_{32}H_{44}N_2O_7 + H)^+$: 569.3227, Found: 569.3239.

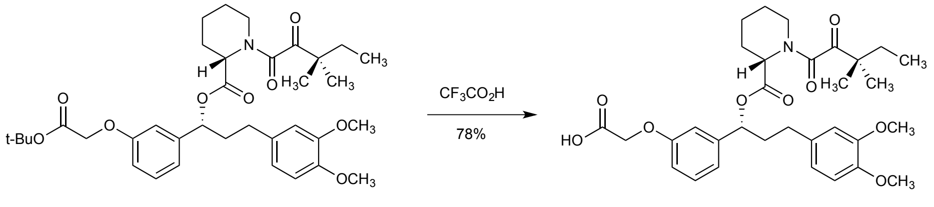

S3 **01-041**

2-(3-((*R*)-3-(3,4-dimethoxyphenyl)-1-(((*S*)-1-(3,3-dimethyl-2-oxopentanoyl)piperidine-2-carbonyl)oxy)propyl)phenoxy)acetic acid (**01-041**)

A solution of [(1R)-1-[3-(2-tert-butoxy-2-oxo-ethoxy)phenyl]-3-(3,4-dimethoxyphenyl)propyl] (2S)-1-(3,3-dimethyl-2-oxo-pentanoyl)piperidine-2-carboxylate[4] (453 mg, 0.71 mmol) in dichloromethane (1.42 mL) was cooled to 0 ºC, then trifluoroacetic acid (1.42 mL) was added dropwise. The resulting yellow solution was stirred at 0 ºC until TLC analysis (50% ethyl acetate–hexanes) showed full consumption of the starting material (~2 h). The reaction solution was concentrated in vacuo to afford the product as a brown liquid. The liquid solidified slowly upon standing under vacuum, giving rise to an off-white powder (322 mg, 78%).

6:1 mixture of rotamers.
^{1}H NMR (400 MHz, Chloroform-*d*) δ 7.32 – 7.23 (m, 1H), 6.93 (d, *J* = 7.7 Hz, 1H), 6.90 – 6.84 (m, 2H), 6.78 (d, *J* = 7.9 Hz, 1H), 6.68 (d, *J* = 7.7 Hz, 2H), 5.74 (dd, *J* = 8.3, 5.3 Hz, 1H), 5.30 (d, *J* = 7.8 Hz, 1H), 4.75 – 4.61 (m, 2H), 3.86 (s, 3H), 3.85 (s, 3H), 3.41 – 3.32 (m, 1H), 3.21 (td, *J* = 13.0, 3.1 Hz, 1H), 2.70 – 2.48 (m, 2H), 2.39 (d, *J* = 13.8 Hz, 1H), 2.31 – 2.17 (m, 1H), 2.13 – 2.03 (m, 1H), 1.86 – 1.58 (m, 5H), 1.58 – 1.31 (m, 2H), 1.20 (s, 3H), 1.18 (s, 3H), 0.87 (t, *J* = 7.4 Hz, 3H). Number of protons: Expected: 41, Calculated from peak integration: 40.

HRMS (ESI): Calcd for $(C_{32}H_{41}NO_9 + H)^+$: 584.2859, Found: 584.2852.

[4] Keenan, T. et al *Bioorg. Med. Chem.* **1998**, *6*, 1309–1335.

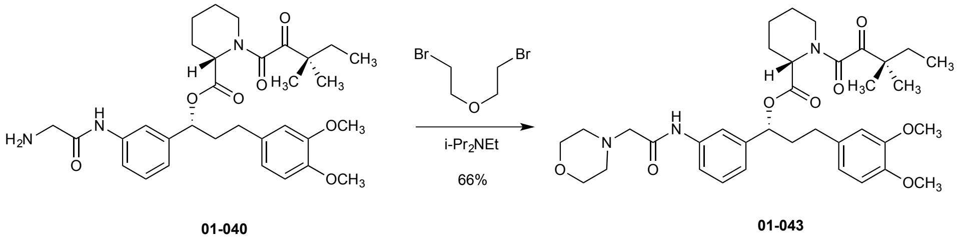

(*R*)-3-(3,4-dimethoxyphenyl)-1-(3-(2-morpholinoacetamido)phenyl)propyl (*S*)-1-(3,3-dimethyl-2-oxopentanoyl)piperidine-2-carboxylate (**01-043**)

A 1-dram vial was charged with 01-040 (50 mg, 0.090 mmol) and a stir bar. DMSO (0.2 mL) and *N*,*N*-diisopropylethylamine (45 µL, 0.26 mmol) were added sequentially. 1-bromo-2-(2-bromoethoxy)ethane (30 mg, 0.13 mmol) was added via syringe. The resulting mixture was stirred at 23 ºC and the reaction progress was monitored by TLC analysis (10% methanol–dichloromethane). After 5 h, the reaction mixture was warmed to 55 ºC. In a total of 22 h, TLC analysis showed full conversion. The reaction mixture was diluted with 50% acetonitrile–water to a volume of 4.7 mL, and the solution was purified by reverse-phase HPLC (Waters XBridge C18 column 5 µm particle size 30 x 250 mm, 5–95% acetonitrile–water + 0.1% formic acid, 40 min, 20 mL/min) to afford the product (**01-043**) as a white solid (37 mg, 66%).

6:1 mixture of rotamers.
^{1}H NMR (400 MHz, Methanol-d_4) δ 8.16 (s, 1H), 7.75 (t, *J* = 1.8 Hz, 1H), 7.53 – 7.46 (m, 1H), 7.35 (t, *J* = 7.9 Hz, 1H), 7.14 (d, *J* = 7.6 Hz, 1H), 6.87 (d, *J* = 8.2 Hz, 1H), 6.84 – 6.78 (m, 1H), 6.74 (dd, *J* = 8.1, 1.8 Hz, 1H), 5.75 (dd, *J* = 8.6, 4.9 Hz, 1H), 5.24 (d, *J* = 5.4 Hz, 1H), 3.83 (d, *J* = 1.1 Hz, 3H), 3.81 (s, 3H), 3.46 – 3.38 (m, 1H), 3.27 (td, *J* = 13.2, 3.1 Hz, 1H), 2.73 (s, 6H), 2.69 – 2.53 (m, 3H), 2.42 – 2.20 (m, 3H), 2.15 – 2.02 (m, 2H), 1.80 – 1.55 (m, 5H), 1.55 – 1.29 (m, 2H), 1.25 (s, 3H), 1.23 (s, 3H), 0.90 (t, *J* = 7.4 Hz, 3H). Number of protons: Expected: 49, Calculated from peak integration: 48.

HRMS (ESI): Calcd for $(C_{36}H_{49}N_3O_8 + H)^+$: 652.3598, Found: 652.3613.

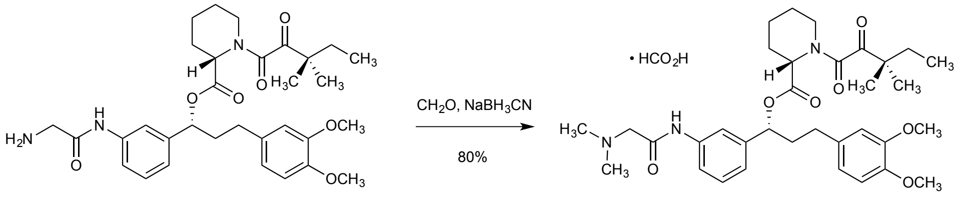

01-040 **01-044**

(R)-3-(3,4-dimethoxyphenyl)-1-(3-(2-(dimethylamino)acetamido)phenyl)propyl (S)-1-(3,3-dimethyl-2-oxopentanoyl)piperidine-2-carboxylate (**01-044**)

A 1-dram vial was charged with 01-040 (50 mg, 0.090 mmol), a stir bar, and 9:1 methanol:acetic acid (0.5 mL). Formaldehyde (37% aqueous solution, 24 µL, 0.86 mmol) was added via pipette. Sodium cyanoborohydride (11 mg, 0.17 mmol) was added in one portion, and the resulting solution was stirred at 23 ºC. In 3 h, TLC analysis (10% methanol–dichloromethane + 1% 30% aqueous ammonium hydroxide solution) showed full consumption of starting material and formation of a less polar product. The reaction mixture was diluted with 50% acetonitrile–water to a final volume of 5 mL, and the resulting solution was purified by reverse-phase HPLC (5–95% acetonitrile–water with 0.1% formic acid, 50 min, 20 mL/min) to afford the product (**01-044**) as a white foam (45 mg, 80%).

6:1 mixture of rotamers.
^{1}H NMR (400 MHz, Methanol-d_4) δ 8.32 (s, 1H), 7.75 (t, J = 1.8 Hz, 1H), 7.51 – 7.42 (m, 1H), 7.37 (t, J = 7.9 Hz, 1H), 7.17 (d, J = 7.6 Hz, 1H), 6.88 (d, J = 7.4 Hz, 1H), 6.81 (d, J = 1.9 Hz, 1H), 6.74 (dd, J = 8.2, 1.9 Hz, 1H), 5.74 (dd, J = 8.8, 5.0 Hz, 1H), 5.24 (d, J = 5.5 Hz, 1H), 3.95 (br s, 2H), 3.83 (s, 3H), 3.82 (s, 3H), 3.47 – 3.38 (m, 1H), 3.31 – 3.20 (m, 1H), 2.89 (s, 6H), 2.75 – 2.53 (m, 2H), 2.41 – 2.19 (m, 2H), 2.09 (dddd, J = 12.4, 9.1, 5.7, 3.7 Hz, 1H), 1.89 – 1.57 (m, 5H), 1.57 – 1.29 (m, 2H), 1.25 (s, 3H), 1.23 (s, 3H), 0.90 (t, J = 7.4 Hz, 3H). Number of protons: Expected: 49, Calculated from peak integration: 47.

HRMS (ESI): Calcd for $(C_{34}H_{47}N_3O_7 + H)^+$: 610.3492, Found: 610.3473.

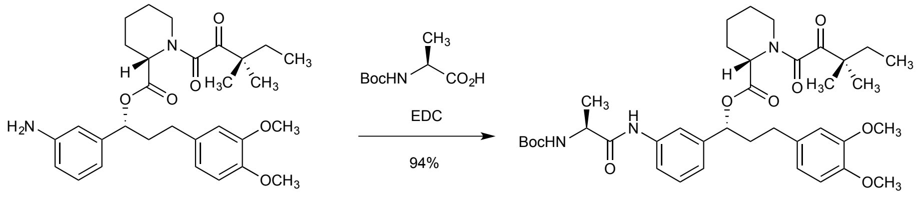

SLF S4

(*R*)-1-(3-((*S*)-2-((*tert*-butoxycarbonyl)amino)propanamido)phenyl)-3-(3,4-dimethoxyphenyl)propyl (*S*)-1-(3,3-dimethyl-2-oxopentanoyl)piperidine-2-carboxylate (**S4**)

EDC (55 mg, 0.29 mmol) was added in one portion to a stirred solution of SLF (100 mg, 0.190 mmol) and D-Boc-Alanine (54 mg, 0.29 mmol) in dichloromethane (9.5 mL) at 0 ºC. The resulting mixture was allowed to warm to 23 ºC over 30 min, then was kept stirred at 23 ºC. In 2 h, TLC analysis (50% ethyl acetate–hexanes) did not reveal any new spot but LC-MS analysis indicated that a new product with higher mass was being formed. In a total of 20 h, LC-MS analysis showed no detectable starting material. The reaction mixture was directly loaded onto a silica gel column and purified by column chromatography (20–50% ethyl acetate–hexanes) to afford the product (**S4**) as a colorless wax (125 mg, 94%).

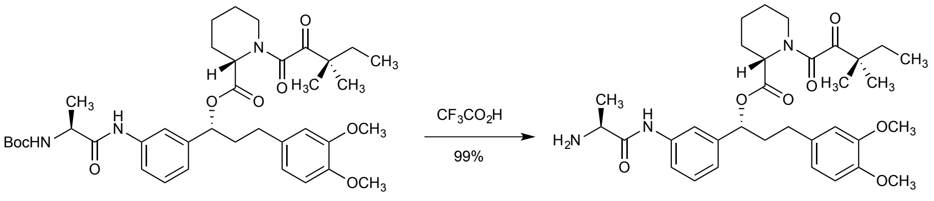

S4 01-059

(*R*)-1-(3-((*S*)-2-aminopropanamido)phenyl)-3-(3,4-dimethoxyphenyl)propyl (*S*)-1-(3,3-dimethyl-2-oxopentanoyl)piperidine-2-carboxylate (**01-059**)

Trifluoroacetic acid (0.5 mL) was added dropwise to a solution of **S4** (125 mg, 0.18 mmol) in dichloromethane (0.5 mL) at 23 ºC. The resulting clear solution was allowed to stand at 23 ºC for 1 h. The solution was concentrated in vacuo, and the residue was partitioned between 10 mL dichloromethane and 10 mL saturated sodium bicarbonate solution. The layers were separated, and the aqueous layer was extracted with dichloromethane (2 x 10 mL). The combined organic layers were dried over sodium sulfate, and the dried solution was concentrated to afford the product (**01-059**) as a white foam (108 mg, 99%).

6:1 mixture of rotamers.

^{1}H NMR (400 MHz, Chloroform-*d*) δ 9.52 (s, 1H), 7.71 – 7.44 (m, 2H), 7.30 (t, *J* = 7.9 Hz, 1H), 7.11 – 6.99 (m, 1H), 6.84 – 6.72 (m, 1H), 6.72 – 6.64 (m, 2H), 5.79 (dd, *J* = 8.0, 5.5 Hz, 1H), 5.38 – 5.30 (m, 1H), 3.86 (s, 3H), 3.85 (s, 3H), 3.70 – 3.56 (m, 1H), 3.36 (d, *J* =

13.1 Hz, 1H), 3.16 (t, *J* = 12.9 Hz, 1H), 2.69 – 2.48 (m, 2H), 2.37 (d, *J* = 13.6 Hz, 1H), 2.24 (ddd, *J* = 13.9, 10.4, 7.0 Hz, 1H), 2.14 – 2.02 (m, 1H), 1.81 – 1.51 (m, 5H), 1.52 – 1.35 (m, 5H), 1.23 (s, 3H), 1.22 (s, 3H), 0.89 (t, *J* = 7.5 Hz, 3H). Number of protons: Expected: 45, Calculated from peak integration: 43.

HRMS (ESI): Calcd for $(C_{33}H_{45}N_3O_7 + H)^+$: 596.3336, Found: 596.3351.

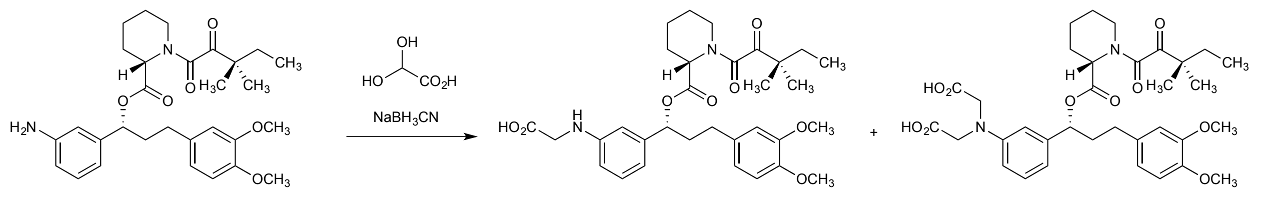

(3-((*R*)-3-(3,4-dimethoxyphenyl)-1-(((*S*)-1-(3,3-dimethyl-2-oxopentanoyl)piperidine-2-carbonyl)oxy)propyl)phenyl)glycine (**01-060A**)
2,2'-((3-((*R*)-3-(3,4-dimethoxyphenyl)-1-(((*S*)-1-(3,3-dimethyl-2-oxopentanoyl)piperidine-2-carbonyl)oxy)propyl)phenyl)azanediyl)diacetic acid (**01-060B**)

An oven-dried 1-dram vial was charged with SLF (50 mg, 0.10 mmol), glyoxylic acid (18 mg, 0.19 mmol) and a magnetic stir bar. 9:1 Methanol:acetic acid (0.5 mL) was added and the resulting solution was cooled to 0 ºC. Sodium cyanoborohydride (12 mg, 0.19 mmol) was added in one portion. The mixture was allowed to stir at 4 ºC and the progress was monitored by LC-MS. In 1 h, LC-MS indicated that the starting material had been fully consumed and two products had formed in roughly 1:1 ratio. To avoid further bis-alkylation, the reaction mixture was immediately diluted with 50% acetonitrile–water to a volume of 5 mL. The resulting solution was purified by reverse-phase HPLC (5–95% acetonitrile–water + 0.1% formic acid, 50 min) to afford the two products as two discrete peaks, both as white powders. 01-060B (26 mg, 43%) eluted faster; 01-060A (21 mg, 38%) eluted slower.

01-060A
6:1 mixture of rotamers.
^{1}H NMR (400 MHz, Methanol-*d*$_4$) δ 7.18 – 7.09 (m, 1H), 6.87 (d, *J* = 8.2 Hz, 1H), 6.85 – 6.76 (m, 1H), 6.76 – 6.70 (m, 1H), 6.68 (d, *J* = 7.9 Hz, 1H), 6.65 – 6.56 (m, 2H), 5.72 – 5.65 (m, 1H), 5.24 (d, *J* = 5.4 Hz, 1H), 3.93 – 3.87 (m, 2H), 3.83 (s, 3H), 3.81 (s, 3H), 3.45 – 3.38 (m, 1H), 3.19 (td, *J* = 13.1, 3.1 Hz, 1H), 2.69 – 2.52 (m, 2H), 2.35 (d, *J* = 13.6 Hz, 1H), 2.31 – 2.15 (m, 1H), 2.13 – 1.97 (m, 1H), 1.87 – 1.59 (m, 5H), 1.59 – 1.28 (m, 2H), 1.26 (s, 3H), 1.24 (s, 3H), 0.91 (t, *J* = 7.5 Hz, 3H). Number of protons: Expected: 42, Calculated from peak integration: 40.
HRMS (ESI): Calcd for $(C_{32}H_{42}N_2O_8 + H)^+$: 583.3019, Found: 583.3010.

01-060B
6:1 mixture of rotamers.
^{1}H NMR (400 MHz, Methanol-*d*$_4$) δ 7.23 (t, *J* = 7.9 Hz, 1H), 6.87 (d, *J* = 8.2 Hz, 1H), 6.85 – 6.69 (m, 3H), 6.65 – 6.56 (m, 2H), 5.71 (dd, *J* = 8.4, 5.3 Hz, 1H), 5.23 (d, *J* = 5.2 Hz, 1H), 4.26 – 4.19 (m, 4H), 3.83 (s, 3H), 3.82 (s, 3H), 3.42 (d, *J* = 13.5 Hz, 1H), 3.19 (td, *J* = 13.2, 3.0 Hz, 1H), 2.60 (h, *J* = 6.7, 6.2 Hz, 2H), 2.35 (d, *J* = 13.6 Hz, 1H), 2.30 – 2.17 (m, 1H), 2.14 – 2.01 (m, 1H), 1.72 (dtdd, *J* = 16.2, 12.6, 8.4, 4.7 Hz, 5H), 1.57 – 1.29 (m,

2H), 1.26 (s, 3H), 1.24 (s, 3H), 0.91 (t, *J* = 7.4 Hz, 3H). Number of protons: Expected: 42, Calculated from peak integration: 40.
HRMS (ESI): Calcd for $(C_{33}H_{44}N_2O_{10} + H)^+$: 641.3074, Found: 641.3093.

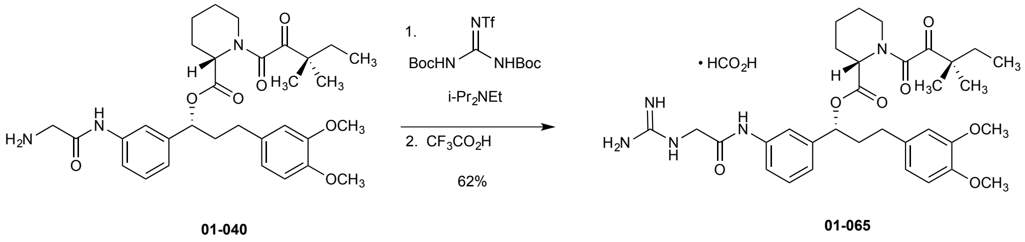

(*R*)-3-(3,4-dimethoxyphenyl)-1-(3-(2-guanidinoacetamido)phenyl)propyl (*S*)-1-(3,3-dimethyl-2-oxopentanoyl)piperidine-2-carboxylate (**01-065**)

An oven-dried 2-mL vial was charged with 01-040 (50 mg, 0.090 mmol), tert-butyl N-[N-tert-butoxycarbonyl-N'-(trifluoromethylsulfonyl)carbamimidoyl]carbamate (50 mg, 0.13 mmol), and a magnetic stir bar. dichloromethane (0.50 mL) and *N*,*N*-diisoproylethylamine (30 µL, 0.17 mmol) were added sequentially via syringe. The resulting mixture was allowed to stir at 23 ºC. In 4 h, LC-MS showed full consumption of the starting material and formation of the desired product mass (Boc protected). Trifluoroacetic acid (0.5 mL) was added to the reaction mixture via syringe. After stirring for another 1 h, LC-MS analysis showed full deprotection of the Boc groups. The reaction mixture was concentrated under reduced pressure. The residue was diluted with 50% acetonitrile–water to a volume of 4.7 mL, and the solution was purified by reverse-phase HPLC (Waters XBridge C18 column 5 µm particle size 30 x 250 mm, 5–95% acetonitrile–water + 0.1% formic acid, 40 min, 20 mL/min) to afford the product (**01-065**) as a white solid (33 mg, 62%).

6:1 mixture of rotamers.
^{1}H NMR (400 MHz, Methanol-d_4) δ 8.35 (s, 1H), 7.78 (t, *J* = 1.9 Hz, 1H), 7.44 (ddd, *J* = 8.1, 2.2, 1.1 Hz, 1H), 7.35 (t, *J* = 7.8 Hz, 1H), 7.18 – 7.11 (m, 1H), 6.87 (d, *J* = 8.2 Hz, 1H), 6.81 (d, *J* = 2.0 Hz, 1H), 6.74 (dd, *J* = 8.2, 2.0 Hz, 1H), 5.74 (dd, *J* = 8.8, 5.0 Hz, 1H), 5.26 – 5.21 (m, 1H), 4.11 (s, 2H), 3.83 (s, 3H), 3.82 (s, 3H), 3.42 (d, *J* = 13.4 Hz, 1H), 3.27 (dd, *J* = 13.2, 3.0 Hz, 1H), 2.74 – 2.54 (m, 2H), 2.36 (d, *J* = 14.1 Hz, 1H), 2.32 – 2.18 (m, 1H), 2.15 – 2.01 (m, 1H), 1.83 – 1.55 (m, 5H), 1.55 – 1.28 (m, 2H), 1.25 (s, 3H), 1.23 (s, 3H), 0.90 (t, *J* = 7.5 Hz, 3H). Number of protons: Expected: 47, Calculated from peak integration: 41.

HRMS (ESI): Calcd for $(C_{33}H_{45}N_5O_7 + H)^+$: 624.3397, Found: 624.3381.

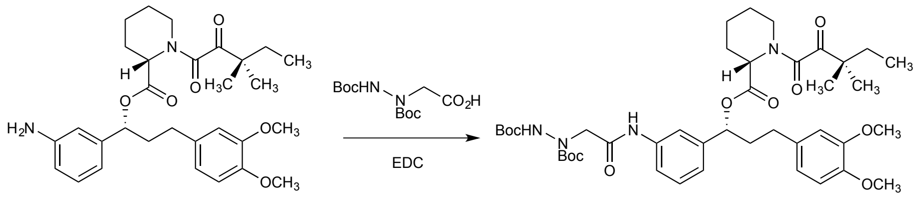

SLF

S5

di-*tert*-butyl 1-(2-((3-((*R*)-3-(3,4-dimethoxyphenyl)-1-(((*S*)-1-(3,3-dimethyl-2-oxopentanoyl)piperidine-2-carbonyl)oxy)propyl)phenyl)amino)-2-oxoethyl)hydrazine-1,2-dicarboxylate (**S5**)

EDC (27 mg, 0.14 mmol) was added in one portion to a stirred solution of SLF (50 mg, 0.10 mmol) and 2-[tert-butoxycarbonyl-(tert-butoxycarbonylamino)amino]acetic acid (42 mg, 0.14 mmol) in dichloromethane (0.19 mL) at 0 ºC. The resulting mixture was allowed to warm to 23 ºC over 30 min, then was kept stirred at 23 ºC. In 3 h, TLC analysis (50% ethyl acetate–hexanes) showed that the reaction was complete. The reaction mixture was directly concentrated under reduced pressure, and the residue was purified by column chromatography (20–50% ethyl acetate–hexanes, 40-g CombiFlash column) to afford the intermediate **S5** as a white foam.

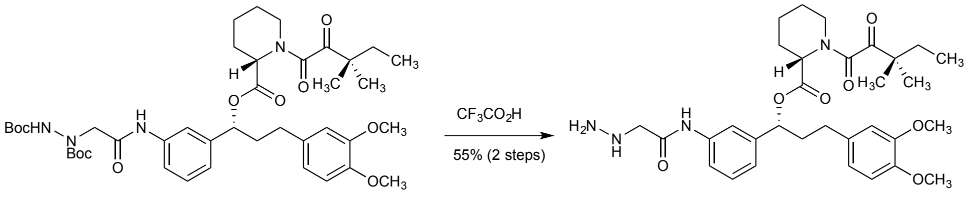

S5

01-070

(*R*)-3-(3,4-dimethoxyphenyl)-1-(3-(2-hydrazineylacetamido)phenyl)propyl (*S*)-1-(3,3-dimethyl-2-oxopentanoyl)piperidine-2-carboxylate (**01-079**)

The intermediate product was dissolved in dichloromethane (0.5 mL) was cooled to 0 ºC, then trifluoroacetic acid (0.5 mL) was added dropwise. The resulting yellow solution was stirred at 0 ºC until TLC analysis (50% ethyl acetate–hexanes, mini-workup with ether/sodium bicarbonate) showed full consumption of the starting material (~2 h). The reaction solution was concentrated in vacuo, and the residue was partitioned between dichloromethane (10 mL) and saturated sodium bicarbonate solution (10 mL). The layers were separated, and the aqueous layer was extracted with dichloromethane (2 x 10 mL). The combined organic layers were dried over sodium sulfate, and the dried solution was concentrated in vacuo to afford the product (**01-070**) as a white foam (29 mg, 55% over 2 steps).

6:1 mixture of rotamers.

^{1}H NMR (400 MHz, Chloroform-*d*) δ 7.65 – 7.46 (m, 2H), 7.33 (t, *J* = 7.9 Hz, 1H), 7.08 (d, *J* = 7.7 Hz, 1H), 6.80 (d, *J* = 8.7 Hz, 1H), 6.75 – 6.68 (m, 2H), 5.84 – 5.74 (m, 1H), 5.35 (d, *J* = 5.6 Hz, 1H), 3.93 (s, 2H), 3.88 (s, 3H), 3.87 (s, 3H), 3.38 (d, *J* = 13.2 Hz, 1H), 3.17 (t, *J* = 12.8 Hz, 1H), 2.72 – 2.47 (m, 2H), 2.38 (d, *J* = 13.3 Hz, 1H), 2.26 (dt, *J* = 16.2, 7.5 Hz, 1H), 2.16 – 2.03 (m, 1H), 1.83 – 1.49 (m, 5H), 1.49 – 1.33 (m, 2H), 1.26 (s, 3H), 1.24 (s, 3H), 0.92 (t, *J* = 7.4 Hz, 3H). Number of protons: Expected: 44, Calculated from peak integration: 40.

HRMS (ESI): Calcd for $(C_{32}H_{44}N_4O_7 + H)^+$: 597.3288, Found: 597.3302.

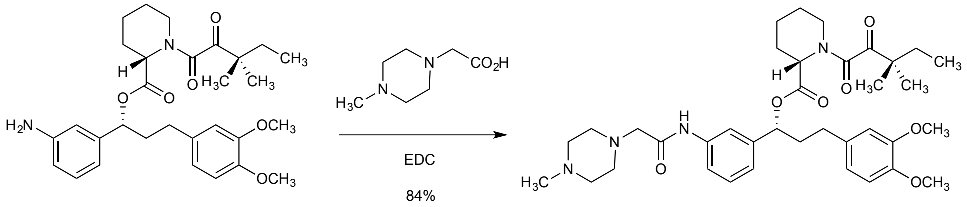

SLF 01-072

(R)-3-(3,4-dimethoxyphenyl)-1-(3-(2-(4-methylpiperazin-1-yl)acetamido)phenyl)propyl (S)-1-(3,3-dimethyl-2-oxopentanoyl)piperidine-2-carboxylate (**01-072**)

An oven-dried 1-dram vial was charged with 2-(4-methylpiperazin-1-yl)acetic acid (30 mg, 0.19 mmol), SLF (50 mg, 0.10 mmol), and a magnetic stir bar. dichloromethane (0.48 mL) was added via syringe, and the resulting solution was cooled to 0 ºC. 3-(ethyliminomethyleneamino)-*N*,*N*-dimethyl-propan-1-amine hydrochloride (37 mg, 0.19 mmol) was added in one portion to the cooled solution, and the resulting mixture was allowed to warm to 23 ºC over 30 min. In 2 h, LC-MS analysis showed full consumption of the starting material. The reaction mixture was directly loaded onto a silica gel column (4-g RediSep) and purified by column chromatography (2–10% methanol in dichloromethane with 0.2–1% saturated aqueous ammonium hydroxide solution) to afford the product (**01-072**) as a white solid (53 mg, 84%).

6:1 mixture of rotamers.
^{1}H NMR (400 MHz, $CDCl_3$) δ 9.13 (s, 1H), 7.58 (d, J = 1.8 Hz, 1H), 7.54 – 7.49 (m, 1H), 7.32 (t, J = 7.9 Hz, 1H), 7.09 (d, J = 7.9 Hz, 1H), 6.84 – 6.75 (m, 1H), 6.75 – 6.65 (m, 2H), 5.78 (dd, J = 8.2, 5.4 Hz, 1H), 5.32 (d, J = 5.4 Hz, 1H), 3.86 (s, 3H), 3.85 (s, 3H), 3.37 (d, J = 13.3 Hz, 1H), 3.20 (td, J = 13.1, 3.1 Hz, 1H), 3.14 (s, 2H), 2.71 – 2.44 (m, 10H), 2.41 – 2.36 (m, 1H), 2.34 (s, 3H), 2.27 (ddt, J = 12.6, 8.6, 4.5 Hz, 1H), 2.16 – 2.00 (m, 1H), 1.80 – 1.55 (m, 5H), 1.54 – 1.29 (m, 2H), 1.23 (s, 3H), 1.21 (s, 3H), 0.89 (t, J = 7.5 Hz, 3H). Number of protons: Expected: 52, Calculated from peak integration: 52.

HRMS (ESI): Calcd for $(C_{37}H_{52}N_4O_7 + H)^+$: 665.3914, Found: 665.3913.

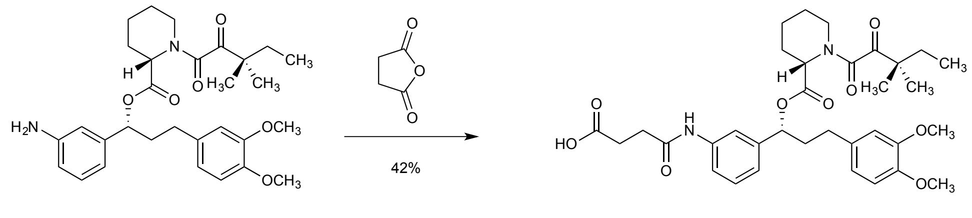

4-((3-((*R*)-3-(3,4-dimethoxyphenyl)-1-(((*S*)-1-(3,3-dimethyl-2-oxopentanoyl)piperidine-2-carbonyl)oxy)propyl)phenyl)amino)-4-oxobutanoic acid (**01-083**)

Succinic anhydride (14 mg, 0.14 mmol) was added to a solution of SLF (50 mg, 0.10 mmol) in DMF (0.48 mL) at 23 ºC. The resulting solution was stirred at 23 ºC for 16 h. The reaction mixture was diluted with 50% acetonitrile–water to a volume of 4.7 mL, and the solution was purified by reverse-phase HPLC (Waters XBridge C18 column 5 µm particle size 30 x 250 mm, 5–95% acetonitrile–water + 0.1% formic acid, 40 min, 20 mL/min) to afford the product (**01-083**) as a white solid (25 mg, 42%).

6:1 mixture of rotamers.
Spectroscopic data was in agreement with that reported by Winter et al. (Winter, G. E. *et al. Science* **2015**, *348*, 1376–81.)
HRMS (ESI): Calcd for $(C_{33}H_{44}N_2O_9 + H)^+$: 625.3125, Found: 625.3102.

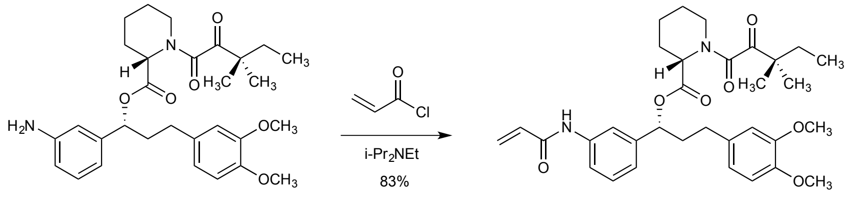

SLF 02-014

(R)-1-(3-acrylamidophenyl)-3-(3,4-dimethoxyphenyl)propyl (S)-1-(3,3-dimethyl-2-oxopentanoyl)piperidine-2-carboxylate (**02-014**)

Acryloyl chloride (12 µL, 0.010 mmol) was added via pipet to a solution of SLF (5 mg, 0.010 mmol) and *N*,*N*-diisopropylethylamine (17 µL, 0.010 mmol) in dichloromethane (0.10 mL) at 0 ºC. The resulting solution was stirred at 0 ºC for 30 min. At this point, LC-MS analysis showed full consumption of starting material and formation of a single peak corresponding to the desired mass. The reaction mixture was concentrated with a stream of dry air. The residue was diluted with 50% acetonitrile–water to a volume of 4.7 mL, and the solution was purified by reverse-phase HPLC (Waters XBridge C18 column 5 µm particle size 30 x 250 mm, 5–95% acetonitrile–water + 0.1% formic acid, 40 min, 20 mL/min) to afford the product (**02-014**) as a white solid (4.6 mg, 83%).

6:1 mixture of rotamers.
^{1}H NMR (400 MHz, Chloroform-*d*) δ 8.14 (s, 1H), 7.97 (d, *J* = 8.2 Hz, 1H), 7.47 (s, 1H), 7.35 (t, *J* = 7.9 Hz, 1H), 7.01 (d, *J* = 7.6 Hz, 1H), 6.79 (d, *J* = 7.9 Hz, 1H), 6.75 – 6.64 (m, 2H), 6.49 (dd, *J* = 16.9, 1.4 Hz, 1H), 6.35 (dd, *J* = 16.9, 10.1 Hz, 1H), 5.86 (dd, *J* = 7.3, 5.6 Hz, 1H), 5.80 (dd, *J* = 10.1, 1.4 Hz, 1H), 5.39 (d, *J* = 5.5 Hz, 1H), 3.88 (s, 3H), 3.87 (s, 3H), 3.32 (d, *J* = 13.6 Hz, 1H), 3.10 – 2.97 (m, 1H), 2.64 – 2.52 (m, 2H), 2.38 (d, *J* = 13.5 Hz, 1H), 2.34 – 2.17 (m, 1H), 2.17 – 2.00 (m, 1H), 1.87 – 1.61 (m, 5H), 1.55 – 1.38 (m, 2H), 1.29 (s, 3H), 1.27 (s, 3H), 0.94 (t, *J* = 7.5 Hz, 3H). Number of protons: Expected: 42, Calculated from peak integration: 42.
HRMS (ESI): Calcd for $(C_{33}H_{42}N_2O_7 + H)^+$: 579.3070, Found: 579.3085.

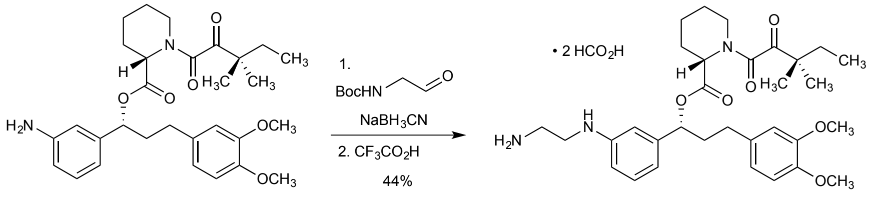

(R)-1-(3-((2-aminoethyl)amino)phenyl)-3-(3,4-dimethoxyphenyl)propyl (S)-1-(3,3-dimethyl-2-oxopentanoyl)piperidine-2-carboxylate (**02-032**)

tert-Butyl *N*-(2-oxoethyl)carbamate (17 mg, 0.10 mmol) was added to a solution of SLF (50 mg, 0.10 mmol) in 9:1 methanol:acetic acid (0.5 mL). The resulting solution was stirred at 23 ºC for 30 min, then sodium cyanoborohydride (9.0 mg, 0.14 mmol) was added in one portion. The resulting mixture was stirred at 23 ºC for another 30 min, at which point LC-MS analysis showed full conversion to the mono-alkylation product. The reaction mixture was concentrated, and the residue was dissolved in trifluoroacetic acid (0.5 mL). After 30 min, LC-MS analysis showed full deprotection of the Boc group. The reaction mixture was concentrated in vacuo. The residue was diluted with 50% acetonitrile–water to a volume of 4.7 mL, and the solution was purified by reverse-phase HPLC (Waters XBridge C18 column 5 µm particle size 30 x 250 mm, 5–95% acetonitrile–water + 0.1% formic acid, 40 min, 20 mL/min) to afford the product (**02-032**) as a white solid (24 mg, 44%).

6:1 mixture of rotamers.
^{1}H NMR (400 MHz, Methanol-d_4) δ 8.43 (s, 2H), 7.18 (t, J = 7.8 Hz, 1H), 6.88 (d, J = 8.2 Hz, 1H), 6.79 (d, J = 2.0 Hz, 1H), 6.78 – 6.70 (m, 2H), 6.69 – 6.60 (m, 2H), 5.69 (dd, J = 8.7, 5.0 Hz, 1H), 5.23 (d, J = 5.6 Hz, 1H), 3.83 (s, 3H), 3.82 (s, 3H), 3.48 – 3.37 (m, 4H), 3.24 (td, J = 13.1, 3.2 Hz, 1H), 3.15 (td, J = 6.0, 2.5 Hz, 2H), 2.72 – 2.47 (m, 2H), 2.37 (d, J = 13.6 Hz, 1H), 2.33 – 2.18 (m, 1H), 2.18 – 1.96 (m, 1H), 1.85 – 1.63 (m, 5H), 1.60 – 1.29 (m, 2H), 1.24 (s, 3H), 1.23 (s, 3H), 0.91 (t, J = 7.5 Hz, 3H). Number of protons: Expected: 49, Calculated from peak integration: 45.
HRMS (ESI): Calcd for $(C_{32}H_{45}N_3O_6 + H)^+$: 568.3386, Found: 568.3440.

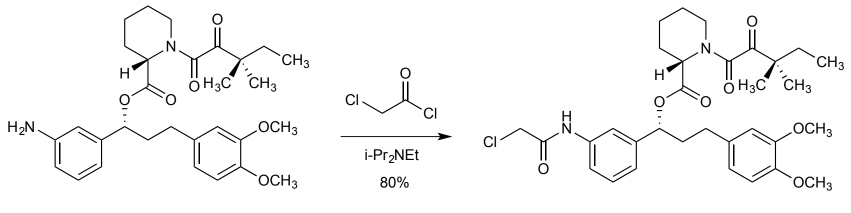

SLF 02-033

(*R*)-1-(3-(2-chloroacetamido)phenyl)-3-(3,4-dimethoxyphenyl)propyl (*S*)-1-(3,3-dimethyl-2-oxopentanoyl)piperidine-2-carboxylate (**02-033**)

2-chloroacetyl chloride (7.6 µL, 0.10 mmol) was added to a solution of SLF (50 mg, 0.10 mmol) and *N*,*N*-diisopropylethylamine (50 µL, 0.29 mmol) in dichloromethane (0.20 mL) at 0 ºC. In 40 min, TLC (50% ethyl acetate–hexanes) showed full consumption of the starting material and formation of a just slightly less polar spot. LC-MS analysis confirmed the identity of the product. The reaction mixture was partitioned between dichloromethane (1 mL) and saturated sodium bicarbonate solution (1 mL). The aqueous layer was extracted with dichloromethane (2 x 1 mL). The combined organic layers were dried over sodium sulfate, and the dried solution was concentrated. The residue was purified by column chromatography (20–50% ethyl acetate–hexanes, 15 min, 4-g CombiFlash column) to afford the product (**02-033**) as a white solid (46 mg, 80%).

6:1 mixture of rotamers.
^{1}H NMR (400 MHz, Methanol-d_4) δ 7.76 (t, *J* = 1.9 Hz, 1H), 7.46 (ddd, *J* = 8.2, 2.2, 1.1 Hz, 1H), 7.34 (t, *J* = 7.9 Hz, 1H), 7.14 (dt, *J* = 7.7, 1.3 Hz, 1H), 6.86 (d, *J* = 8.2 Hz, 1H), 6.80 (d, *J* = 2.0 Hz, 1H), 6.73 (dd, *J* = 8.2, 2.0 Hz, 1H), 5.75 (dd, *J* = 8.8, 5.1 Hz, 1H), 5.24 (d, *J* = 5.2 Hz, 1H), 4.19 (s, 2H), 3.82 (s, 3H), 3.81 (s, 3H), 3.46 – 3.39 (m, 1H), 3.26 (td, *J* = 13.1, 3.0 Hz, 1H), 2.63 (tdd, *J* = 15.4, 8.6, 5.2 Hz, 2H), 2.46 – 2.19 (m, 2H), 2.16 – 2.04 (m, 1H), 1.81 – 1.57 (m, 5H), 1.55 – 1.28 (m, 2H), 1.25 (s, 3H), 1.23 (s, 3H), 0.90 (t, *J* = 7.5 Hz, 3H). Number of protons: Expected: 41, Calculated from peak integration: 40.
HRMS (ESI): Calcd for $(C_{32}H_{41}ClN_2O_7 + H)^+$: 601.2680, Found: 601.2688.

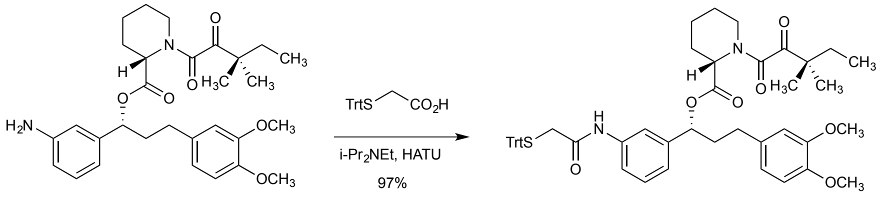

SLF **S6**

(*R*)-3-(3,4-dimethoxyphenyl)-1-(3-(2-(tritylthio)acetamido)phenyl)propyl (*S*)-1-(3,3-dimethyl-2-oxopentanoyl)piperidine-2-carboxylate (**S6**)

N,*N*-diisopropylamine (50 µL, 0.29 mmol) and HATU (54 mg, 0.14 mmol) were added sequentially to a stirred solution of SLF (50 mg, 0.10 mmol) and S-trityl thioglycolic acid (38 mg, 0.11 mmol) in a mixed solvent of dichloromethane (0.19 mL) and DMF (0.20 mL). The resulting solution was stirred at 23 ºC. In 2 h, LC-MS analysis showed full conversion to the desired product. The reaction mixture was partitioned between ether (10 mL) and water (10 mL). The aqueous layer was extracted with ether (2 x 10 mL). The combined organic layers were washed with water (2 x 10 mL), then with brine (10 mL). The washed solution was dried over magnesium sulfate, and the dried solution was concentrated. The residue was purified by column chromatography (20–100% ethyl acetate–hexanes) to afford the intermediate product (**S6**) as a white solid (78 mg, 97%).

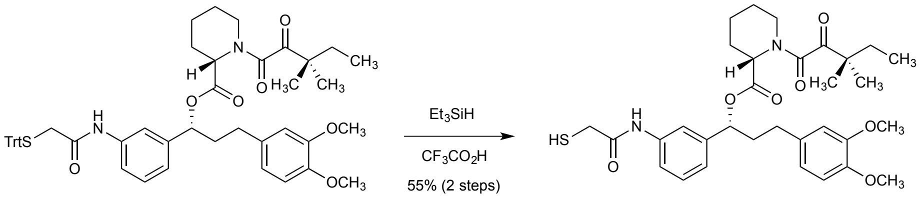

S6 **02-055**

(*R*)-3-(3,4-dimethoxyphenyl)-1-(3-(2-mercaptoacetamido)phenyl)propyl (*S*)-1-(3,3-dimethyl-2-oxopentanoyl)piperidine-2-carboxylate (**02-055**)

Triethylsilane (0.15 mL, 0.93 mmol) and trifluoroacetic acid (40 µL, 0.46 mmol) were sequentially added to a stirred solution of the intermediate (78 mg, 0.090 mmol) in dichloromethane (0.19 mL) at 23 ºC. The resulting yellow solution was stirred at 23 ºC for 1 h, at which point the yellow color had completely faded. The reaction mixture was concentrated, and the residue was purified by column chromatography (50–100% ethyl acetate–hexanes) to afford the product (**02-055**) as a white solid (49 mg, 86%).

6:1 mixture of rotamers.
^{1}H NMR (400 MHz, Chloroform-*d*) δ 8.72 (s, 1H), 7.67 (ddd, *J* = 8.1, 2.2, 1.0 Hz, 1H), 7.49 (d, *J* = 2.0 Hz, 1H), 7.31 (t, *J* = 7.9 Hz, 1H), 7.04 (dt, *J* = 7.7, 1.2 Hz, 1H), 6.76 (d, *J* = 8.7

Hz, 1H), 6.71 – 6.63 (m, 2H), 5.80 (dd, *J* = 7.7, 5.6 Hz, 1H), 5.37 – 5.31 (m, 1H), 3.85 (s, 3H), 3.83 (s, 3H), 3.39 (d, *J* = 9.0 Hz, 2H), 3.36 – 3.29 (m, 1H), 3.15 – 3.01 (m, 1H), 2.64 – 2.48 (m, 2H), 2.48 – 2.31 (m, 2H), 2.30 – 2.17 (m, 1H), 2.09 (t, *J* = 9.0 Hz, 1H), 1.78 – 1.57 (m, 5H), 1.51 – 1.33 (m, 2H), 1.23 (s, 6H), 0.89 (t, *J* = 7.5 Hz, 3H). Number of protons: Expected: 52, Calculated from peak integration: 52.

HRMS (ESI): Calcd for $(C_{32}H_{42}N_2O_7S + H)^+$: 599.2791, Found: 599.2778.

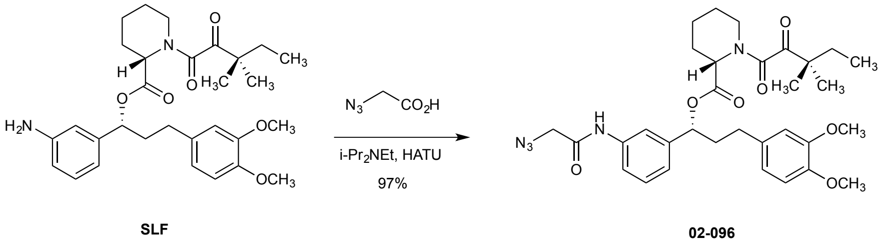

(*R*)-1-(3-(2-azidoacetamido)phenyl)-3-(3,4-dimethoxyphenyl)propyl (*S*)-1-(3,3-dimethyl-2-oxopentanoyl)piperidine-2-carboxylate (**02-096**)

N,*N*-diisopropylamine (50 µL, 0.29 mmol) and HATU (54 mg, 0.14 mmol) were added sequentially to a stirred solution of SLF (50 mg, 0.10 mmol), 2-azidoacetic acid (8.6 µL, 0.11 mmol) in 9:1 dichloromethane:DMF (0.19 mL). In 2 h, LC-MS analysis showed full conversion to the desired product. The reaction mixture was partitioned between ether (10 mL) and water (10 mL). The aqueous layer was extracted with ether (2 x 10 mL). The combined organic layers were washed with water (2 x 10 mL), then with brine (10 mL). The washed solution was dried over magnesium sulfate, and the dried solution was concentrated. The residue was purified by column chromatography (20–100% ethyl acetate–hexanes) to afford the product (**02-096**) as a white solid (56 mg, 97%).

6:1 mixture of rotamers.
^{1}H NMR (400 MHz, Chloroform-*d*) δ 8.29 (s, 1H), 7.71 (ddd, *J* = 8.1, 2.3, 1.0 Hz, 1H), 7.50 (t, *J* = 1.9 Hz, 1H), 7.34 (t, *J* = 7.9 Hz, 1H), 7.07 (d, *J* = 7.5 Hz, 1H), 6.82 – 6.74 (m, 1H), 6.74 – 6.57 (m, 2H), 5.81 (dd, *J* = 7.7, 5.6 Hz, 1H), 5.34 (d, *J* = 5.5 Hz, 1H), 4.17 – 4.10 (m, 2H), 3.86 (s, 3H), 3.85 (s, 3H), 3.33 (d, *J* = 13.7 Hz, 1H), 3.15 – 3.01 (m, 1H), 2.66 – 2.41 (m, 2H), 2.36 (d, *J* = 13.6 Hz, 1H), 2.31 – 2.16 (m, 1H), 2.11 – 1.99 (m, 1H), 1.78 – 1.59 (m, 5H), 1.51 – 1.33 (m, 2H), 1.24 (s, 6H), 0.91 (t, *J* = 7.5 Hz, 3H). Number of protons: Expected: 41, Calculated from peak integration: 41.
HRMS (ESI): Calcd for $(C_{32}H_{41}N_5O_7 + H)^+$: 608.3084, Found: 608.3110.

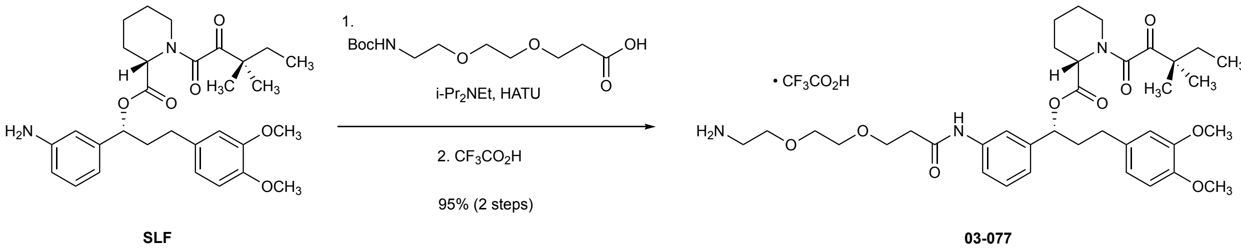

(*R*)-1-(3-(3-(2-(2-aminoethoxy)ethoxy)propanamido)phenyl)-3-(3,4-dimethoxyphenyl)propyl (*S*)-1-(3,3-dimethyl-2-oxopentanoyl)piperidine-2-carboxylate (**03-077**)

N,*N*-diisopropylethylamine (16.6 µL, 0.10 mmol) and HATU (72 mg, 0.19 mmol) were added sequentially to a solution of SLF (50 mg, 0.10 mmol) and 3-[2-[2-(tert-butoxycarbonylamino)ethoxy]ethoxy]propanoic acid (40 mg, 0.14mmol) in 9:1 dichloromethane:DMF (0.2 mL). The resulting mixture was stirred at 23 ºC for 12 h, at which point LC-MS analysis showed full conversion to the desired product. The reaction mixture was partitioned between ethyl acetate (1 mL) and water (1 mL). The aqueous layer was extracted with ethyl acetate (3 x 1 mL). The combined organic layers were dried over sodium sulfate, then was concentrated under reduced pressure. The residue was purified by column chromatography (50–100% ethyl acetate–hexanes, 4-g CombiFlash column) to afford the intermediate product as a yellow oil.

The intermediate product (70 mg, 0.090 mmol) was dissolved in 1:1 dichloromethane:trifluoroacetic acid (1.0 mL). The resulting solution was allowed to stand at 23 ºC for 1 h, then was concentrated under reduced pressure to afford the product as a yellow foam (71 mg, 95%).

6:1 mixture of rotamers.
^{1}H NMR (400 MHz, MeOD) δ 7.70 (t, *J* = 1.9 Hz, 1H), 7.52 – 7.44 (m, 1H), 7.34 (t, *J* = 7.9 Hz, 1H), 7.14 (d, *J* = 8.0 Hz, 1H), 6.88 (d, *J* = 8.1 Hz, 1H), 6.81 (d, *J* = 2.1 Hz, 1H), 6.75 (dd, *J* = 8.2, 2.0 Hz, 1H), 5.73 (dd, *J* = 8.8, 5.0 Hz, 1H), 5.24 (d, *J* = 5.6 Hz, 1H), 3.86 (t, *J* = 6.5 Hz, 2H), 3.84 (s, 3H), 3.82 (s, 3H), 3.71 – 3.64 (m, 8H), 3.42 (d, *J* = 13.9 Hz, 1H), 3.26 (dd, *J* = 13.4, 3.3 Hz, 1H), 3.09 – 2.99 (m, 2H), 2.70 – 2.54 (m, 3H), 2.37 (d, *J* = 14.3 Hz, 1H), 2.34 – 2.22 (m, 1H), 2.15 – 2.03 (m, 1H), 1.84 – 1.59 (m, 5H), 1.59 – 1.29 (m, 2H), 1.25 (s, 3H), 1.24 (s, 3H), 0.90 (t, *J* = 7.5 Hz, 3H). Number of protons: Expected: 54, Calculated from peak integration: 51.
HRMS (ESI): Calcd for $(C_{37}H_{53}N_3O_9 + H)^+$: 684.3860, Found: 684.3851.

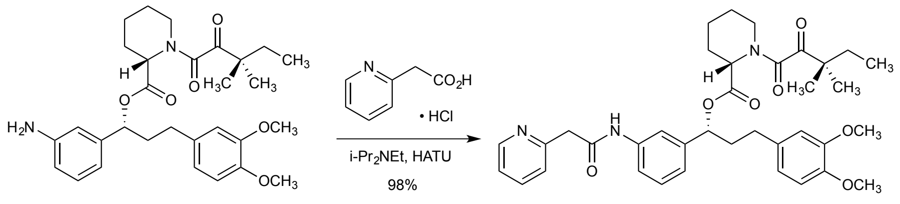

SLF **03-083**

(*R*)-3-(3,4-dimethoxyphenyl)-1-(3-(2-(pyridin-2-yl)acetamido)phenyl)propyl (*S*)-1-(3,3-dimethyl-2-oxopentanoyl)piperidine-2-carboxylate (**03-083**)

N,*N*-Diisopropylethylamine (16.6 µL, 0.10 mmol) and HATU (72 mg, 0.19 mmol) were added sequentially to a solution of SLF (50 mg, 0.10 mmol) and 2-(2-pyridyl)acetic acid hydrochloride (25 mg, 0.14 mmol) in 9:1 dichloromethane:DMF (0.2 mL). The resulting mixture was stirred at 23 ºC for 12 h, at which point LC-MS analysis showed full conversion to the desired product. The reaction mixture was partitioned between ethyl acetate (1 mL) and water (1 mL). The aqueous layer was extracted with ethyl acetate (3 x 1 mL). The combined organic layers were dried over sodium sulfate, then was concentrated under reduced pressure. The residue was purified by column chromatography (50–100% ethyl acetate–hexanes, 4-g CombiFlash column) to afford the product as a white solid (60 mg, 98%).

6:1 mixture of rotamers.
^{1}H NMR (400 MHz, Chloroform-*d*) δ 9.91 (s, 1H), 8.62 (dd, *J* = 5.3, 1.7 Hz, 1H), 7.93 (t, *J* = 7.6 Hz, 1H), 7.61 (s, 1H), 7.54 (t, *J* = 7.5 Hz, 2H), 7.44 (t, *J* = 6.5 Hz, 1H), 7.34 – 7.23 (m, 1H), 7.05 (d, *J* = 7.7 Hz, 1H), 6.82 – 6.73 (m, 1H), 6.74 – 6.63 (m, 2H), 5.76 (dd, *J* = 8.1, 5.4 Hz, 1H), 5.32 (d, *J* = 5.4 Hz, 1H), 4.05 (s, 2H), 3.85 (s, 3H), 3.85 (s, 3H), 3.37 (d, *J* = 13.3 Hz, 1H), 3.18 (td, *J* = 13.0, 3.1 Hz, 1H), 2.69 – 2.46 (m, 2H), 2.36 (d, *J* = 13.5 Hz, 1H), 2.33 – 2.19 (m, 1H), 2.19 – 1.99 (m, 1H), 1.79 – 1.57 (m, 5H), 1.57 – 1.29 (m, 2H), 1.23 (s, 3H), 1.21 (s, 3H), 0.88 (t, *J* = 7.4 Hz, 3H). Number of protons: Expected: 45, Calculated from peak integration: 45.
HRMS (ESI): Calcd for $(C_{37}H_{45}N_3O_7 + H)^+$: 644.3336, Found: 644.3350.

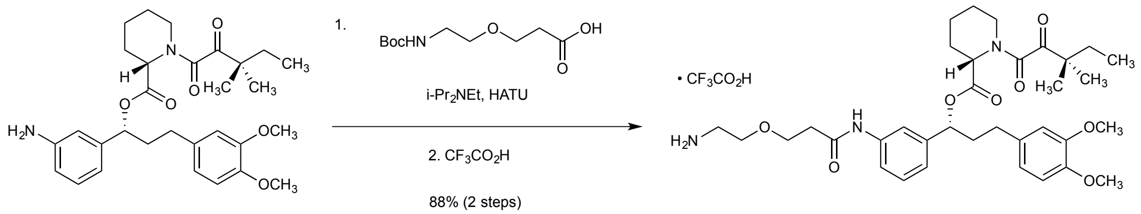

03-084

(R)-1-(3-(3-(2-aminoethoxy)propanamido)phenyl)-3-(3,4-dimethoxyphenyl)propyl (S)-1-(3,3-dimethyl-2-oxopentanoyl)piperidine-2-carboxylate (**03-084**)

N,*N*-diisopropylethylamine (16.6 µL, 0.10 mmol) and HATU (72 mg, 0.19 mmol) were added sequentially to a solution of SLF (50 mg, 0.10 mmol) and 3-[2-(*tert*-butoxycarbonylamino)ethoxy]propanoic acid (33 mg, 0.14 mmol) in 9:1 dichloromethane:DMF (0.2 mL). The resulting mixture was stirred at 23 ºC for 12 h, at which point LC-MS analysis showed full conversion to the desired product. The reaction mixture was partitioned between ethyl acetate (1 mL) and water (1 mL). The aqueous layer was extracted with ethyl acetate (3 x 1 mL). The combined organic layers were dried over sodium sulfate, then was concentrated under reduced pressure. The residue was purified by column chromatography (50–100% ethyl acetate–hexanes, 4-g CombiFlash column) to afford the product as a yellow oil. The oil was dissolved in 1:1 dichloromethane:trifluoroacetic acid (1.0 mL). The resulting colorless solution was allowed to stand at 23 °C for 1 h, then was concentrated in vacuo to afford the product (**03-084**) as a white foam (62 mg, 88%).

6:1 mixture of rotamers.

^{1}H NMR (400 MHz, Acetonitrile-d_3) δ 8.85 (s, 1H), 7.68 (s, 1H), 7.51 (d, J = 8.1 Hz, 1H), 7.35 (t, J = 7.9 Hz, 1H), 7.11 (d, J = 7.6 Hz, 1H), 6.86 (d, J = 8.2 Hz, 1H), 6.80 (d, J = 2.0 Hz, 1H), 6.74 (dd, J = 8.1, 2.0 Hz, 1H), 5.70 (dd, J = 8.7, 4.8 Hz, 1H), 5.24 (d, J = 5.6 Hz, 1H), 3.80 (s, 3H), 3.79 (s, 3H), 3.72 (dd, J = 6.2, 4.1 Hz, 2H), 3.38 (d, J = 14.0 Hz, 1H), 3.25 (dd, J = 12.9, 3.1 Hz, 1H), 3.17 (s, 4H), 2.72 – 2.51 (m, 4H), 2.37 (d, J = 13.9 Hz, 1H), 2.32 – 2.17 (m, 1H), 2.17 – 2.04 (m, 1H), 1.84 – 1.56 (m, 5H), 1.55 – 1.33 (m, 2H), 1.21 (s, 3H), 1.20 (s, 3H), 0.87 (t, J = 7.5 Hz, 3H). Number of protons: Expected: 50, Calculated from peak integration: 47.

HRMS (ESI): Calcd for $(C_{35}H_{49}N_3O_8 + H)^+$: 640.3598, Found: 640.3611.

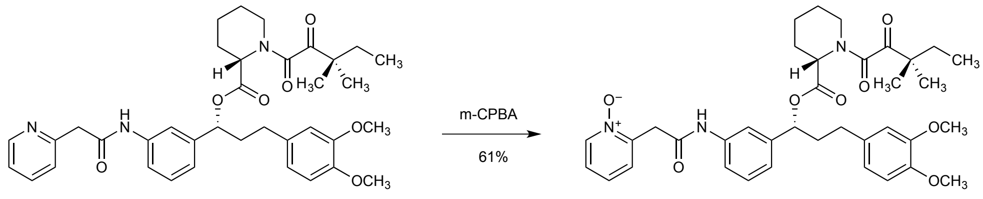

03-083 **03-087**

2-(2-((3-((*R*)-3-(3,4-dimethoxyphenyl)-1-(((*S*)-1-(3,3-dimethyl-2-oxopentanoyl)piperidine-2-carbonyl)oxy)propyl)phenyl)amino)-2-oxoethyl)pyridine 1-oxide (**03-087**)

3-chlorobenzenecarboperoxoic acid (5.4 mg, 0.020 mmol) was added to an ice-cold solution of 03-083 (10 mg, 0.02 0mmol) in dichloromethane (0.16 mL). The resulting pale yellow solution was stirred at 0 °C for 1 h. LC-MS analysis showed complete conversion to the desired m/z. Triethylamine (10 µL) was added to consume the excess *m*CPBA. After warming to 23 °C, the solution was concentrated under reduced pressure. The residue was diluted with 50% acetonitrile–water to a volume of 4.7 mL, and the solution was purified by reverse-phase HPLC (Waters XBridge C18 column 5 µm particle size 30 x 250 mm, 5–95% acetonitrile–water + 0.1% formic acid, 40 min, 20 mL/min) to afford the product (**03-087**) as a white solid (6.3 mg, 61%).

6:1 mixture of rotamers.
^{1}H NMR (400 MHz, Chloroform-*d*) δ 10.91 (s, 1H), 8.46 – 8.37 (m, 1H), 7.60 – 7.49 (m, 3H), 7.45 (t, *J* = 7.7 Hz, 1H), 7.36 (t, *J* = 7.0 Hz, 1H), 7.33 – 7.24 (m, 1H), 7.09 – 7.00 (m, 1H), 6.80 (d, *J* = 8.8 Hz, 1H), 6.74 – 6.67 (m, 2H), 5.77 (dd, *J* = 8.1, 5.6 Hz, 1H), 5.33 (d, *J* = 5.4 Hz, 1H), 4.12 (s, 2H), 3.88 (s, 3H), 3.87 (s, 3H), 3.40 (d, *J* = 13.4 Hz, 1H), 3.22 (td, *J* = 13.0, 3.1 Hz, 1H), 2.68 – 2.48 (m, 2H), 2.38 (d, *J* = 13.7 Hz, 1H), 2.33 – 2.17 (m, 1H), 2.14 – 2.02 (m, 1H), 1.84 – 1.58 (m, 5H), 1.58 – 1.30 (m, 2H), 1.26 (s, 3H), 1.23 (s, 3H), 0.90 (t, *J* = 7.4 Hz, 3H). Number of protons: Expected: 45, Calculated from peak integration: 45.
HRMS (ESI): Calcd for $(C_{37}H_{45}N_3O_8 + H)^+$: 660.3285, Found: 660.3268.

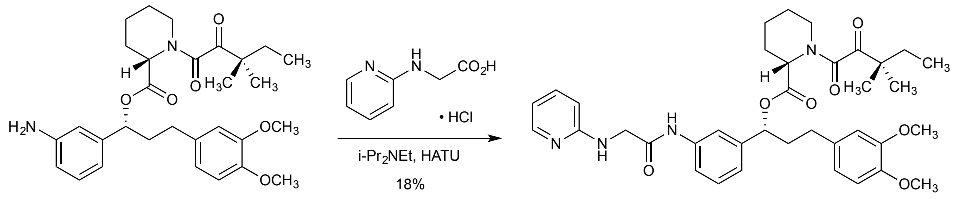

SLF

03-091

(R)-3-(3,4-dimethoxyphenyl)-1-(3-(2-(pyridin-2-ylamino)acetamido)phenyl)propyl (S)-1-(3,3-dimethyl-2-oxopentanoyl)piperidine-2-carboxylate (**03-091**)

HATU (72 mg, 0.19 mmol) was added to a mixture of SLF (50 mg, 0.10 mmol), 2-(2-pyridylamino)acetic acid hydrochloride (27 mg, 0.14 mmol), *N*,*N*-diisopropylethylamine (34 µL, 0.20 mmol) in 9:1 dichloromethane:DMF (0.2 mL). The resulting mixture was stirred at 23 ºC for 12 h, at which point LC-MS analysis showed ~50% conversion to the desired product. Additional 2-(2-pyridylamino)acetic acid hydrochloride (27 mg, 0.14 mmol) and HATU (72 mg, 0.19 mmol) were added, and the mixture was stirred for another 1 h. LC-MS analysis showed no further progress. The reaction mixture was partitioned between ethyl acetate (1 mL) and water (1 mL). The aqueous layer was extracted with ethyl acetate (3 x 1 mL). The combined organic layers were dried over sodium sulfate, then was concentrated under reduced pressure. The residue was purified by column chromatography (50–100% ethyl acetate–hexanes, 4-g CombiFlash column) to afford the product (**03-091**) as a yellow solid (11.2 mg, 18%).

6:1 mixture of rotamers.
^{1}H NMR (400 MHz, Chloroform-*d*) δ 7.92 (s, 1H), 7.83 (s, 1H), 7.65 (d, J = 8.3 Hz, 1H), 7.59 (s, 1H), 7.36 – 7.28 (m, 1H), 7.20 – 6.99 (m, 3H), 6.87 (t, J = 6.4 Hz, 1H), 6.85 – 6.75 (m, 1H), 6.75 – 6.64 (m, 2H), 5.81 – 5.74 (m, 1H), 5.33 (d, J = 5.2 Hz, 1H), 4.54 – 4.35 (m, 2H), 3.86 (s, 3H), 3.85 (s, 3H), 3.37 (d, J = 13.7 Hz, 1H), 3.26 – 2.99 (m, 2H), 2.66 – 2.46 (m, 2H), 2.37 (d, J = 13.4 Hz, 1H), 2.33 – 2.18 (m, 1H), 2.14 – 2.02 (m, 1H), 1.80 – 1.64 (m, 5H), 1.51 – 1.32 (m, 2H), 1.23 (s, 3H), 1.21 (s, 3H), 0.88 (t, J = 7.4 Hz, 3H). Number of protons: Expected: 46, Calculated from peak integration: 46.
HRMS (ESI): Calcd for $(C_{37}H_{46}N_4O_7 + H)^+$: 659.3444, Found: 659.3436

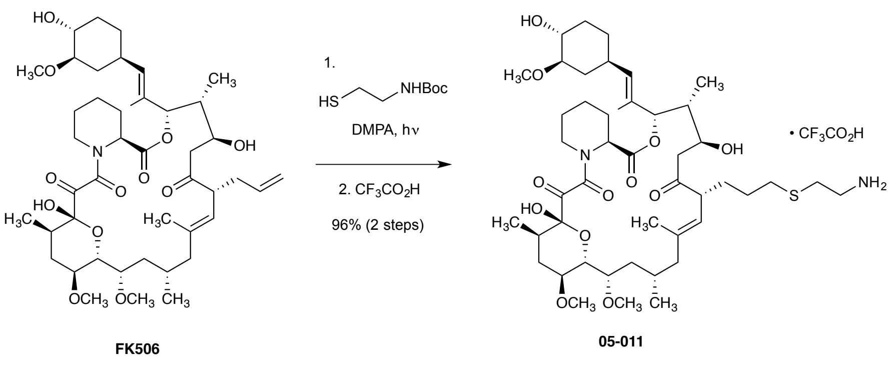

(1R,9S,12S,13R,14S,17R,18E,21S,23S,24R,25S,27R)-17-{3-[(2-aminoethyl)sulfanyl]propyl}-1,14-dihydroxy-12-[(1E)-1-[(1R,3R,4R)-4-hydroxy-3-methoxycyclohexyl]prop-1-en-2-yl]-23,25-dimethoxy-13,19,21,27-tetramethyl-11,28-dioxa-4-azatricyclo[22.3.1.0^{4,9}]octacos-18-ene-2,3,10,16-tetrone (**05-011**)

Tert-butyl *N*-(2-sulfanylethyl)carbamate (23 mg, 0.13 mmol) and dimethoxyphenylacetophenone (DMPA, 3.2 mg, 0.010 mmol) were added sequentially to a solution of FK506 (100 mg, 0.120 mmol) in dichloromethane (1.2 mL) at 23 ºC. After all reactants had dissolved, the vial was placed above a hand-held UV-light operating at 365 nm wavelength (the light was placed upside-down so that the contents of vial were directly irradiated)[5]. The irradiation was maintained for 15 min, at which point TLC analysis (100% ethyl acetate) showed full consumption of FK506 and formation of a slightly more polar product. The reaction solution was directly loaded onto a 12-g silica gel column. Purification by column chromatography (50–100% ethyl acetate–hexanes, 12-g RediSep(R) Rf column, Teledyne ISCO, Lincoln, NE) afforded the intermediate product as a white foam (117 mg, 96%).
Trifluoroacetic acid (0.5 mL) was added dropwise to a solution of the intermediate product (117 mg, 0.120 mmol) in dichloromethane (0.5 mL) at 0 ºC. The resulting mixture was stirred at 0 ºC for 1 h, at which point LC-MS analysis showed full deprotection of the Boc group. The reaction solution was concentrated under reduced pressure and the product was dried azeotropically by rotary evaporation of its suspension in toluene to afford the product as an off-white powder (119 mg, 100%).

NMR: 3:2 mixture of rotamers.
^{1}H NMR (400 MHz, Methanol-d_4) δ 5.29 – 5.07 (m, 2H), 4.95 (d, *J* = 11.7 Hz, 1H), 4.64 (s, 1H), 4.37 (d, *J* = 13.4 Hz, 1H), 4.13 – 3.93 (m, 2H), 3.78 – 3.70 (m, 2H), 3.69 – 3.54 (m, 2H), 3.44 (s, 3H), 3.42 (s, 3H), 3.41 – 3.39 (m, 3H), 3.36 (s, 3H), 3.19 – 3.09 (m, 2H), 3.09 – 2.96 (m, 2H), 2.84 – 2.75 (m, 2H), 2.60 (t, *J* = 6.9 Hz, 2H), 2.43 – 2.26 (m, 4H), 2.27 – 2.10 (m, 4H), 2.10 – 1.89 (m, 4H), 1.84 – 1.73 (m, 6H), 1.73 – 1.60 (m, 6H), 1.61

[5] Guo, Z. F.; Zhang, R.; Liang, F. S. *RSC Adv.*,2014, *4*, 11400.

– 1.29 (m, 8H), 1.04 – 0.98 (m, 2H), 0.98 – 0.87 (m, 9H). Number of protons: Expected: 77, Calculated from peak integration: 74.
HRMS (ESI): Calcd for $(C_{46}H_{76}N_2O_{12}S + H)^+$: 881.5197, Found: 881.5207

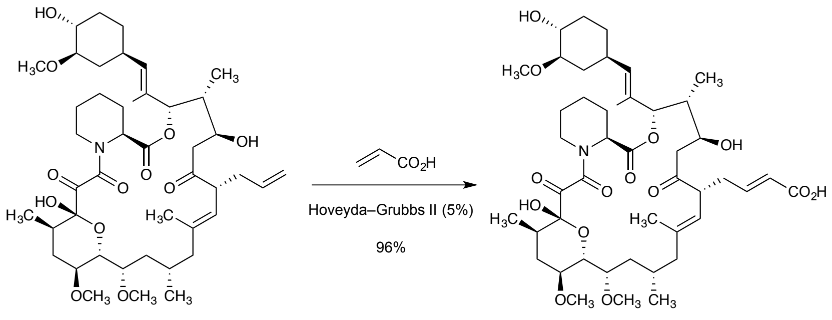

FK506 **05-012**

A flame-dried 10-mL microwave vial was flushed with dry argon, and then was charged with FK506 (100 mg, 0.120 mmol), DCE (1.20 mL), and a magnetic stir bar. Acrylic acid (170 mg, 2.49 mmol) and Grubbs Catalyst 2nd Gen (5.3 mg, 0.010 mmol) were added sequentially. The vial was flushed with argon again and sealed with a rubber cap. The reaction mixture was heated at 85 ºC for 1 h in a CEM Discover SP microwave reactor. After cooling to 23 ºC, TLC analysis (100% ethyl acetate) of the reaction mixture showed full disappearance of the starting material and formation of a highly polar new spot. The reaction solution was concentrated in vacuo. The residue was purified by column chromatography (20–100% ethyl acetate–hexanes, 12-g RediSep(R) Rf column, Teledyne ISCO, Lincoln, NE) to afford the product as a yellow powder (101 mg, 96%).

NMR: 3:2 mixture of rotamers.
^{1}H NMR (400 MHz, Chloroform-*d*) δ 7.01 – 6.88 (m, 1H), 5.86 (d, *J* = 15.5 Hz, 1H), 5.34 (s, 1H), 5.11 (app t, *J* = 8.8 Hz, 1H), 5.03 (app d, *J* = 8.4 Hz, 1H), 4.68 (d, *J* = 5.2 Hz, 1H), 4.46 (d, *J* = 13.9 Hz, 1H), 3.96 – 3.84 (m, 1H), 3.72 (d, *J* = 9.3 Hz, 1H), 3.68 – 3.54 (m, 1H), 3.55 – 3.41 (m, 3H), 3.43 (s, 3H), 3.41 (s, 3H), 3.32 (s, 3H), 3.08 – 2.96 (m, 3H), 2.86 – 2.78 (m, 1H), 2.74 – 2.62 (m, 1H), 2.49 – 2.26 (m, 3H), 2.24 – 2.08 (m, 3H), 2.06 – 1.98 (m, 2H), 1.98 – 1.86 (m, 2H), 1.86 – 1.71 (m, 4H), 1.71 – 1.60 (m, 6H), 1.59 – 1.32 (m, 8H), 1.14 – 1.05 (m, 2H), 1.03 (d, *J* = 6.3 Hz, 3H), 0.96 (d, *J* = 6.4 Hz, 3H), 0.90 (d, *J* = 7.1 Hz, 3H). Number of protons: Expected: 69, Calculated from peak integration: 66.

HRMS (ESI): Calcd for $(C_{45}H_{69}NO_{14} - H)^-$: 846.4640, Found: 846.4601

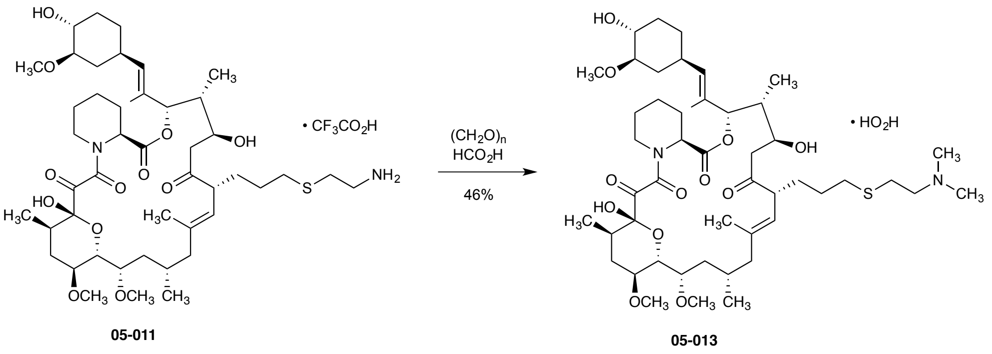

(1R,9S,12S,13R,14S,17R,18E,21S,23S,24R,25S,27R)-17-(3-{[2-(dimethylamino)ethyl]sulfanyl}propyl)-1,14-dihydroxy-12-[(1E)-1-[(1R,3R,4R)-4-hydroxy-3-methoxycyclohexyl]prop-1-en-2-yl]-23,25-dimethoxy-13,19,21,27-tetramethyl-11,28-dioxa-4-azatricyclo[22.3.1.0^{4,9}]octacos-18-ene-2,3,10,16-tetrone (**05-013**)

Paraformaldehyde (3.0 mg, 0.10 mmol) and formic acid (4.6 mg, 0.10 mmol) were added sequentially to a solution of 05-011 (10 mg, 0.010mmol) in chloroform (0.2 mL). The suspension was heated to 70 ºC for 1 h. In 1 h, LC-MS analysis showed formation of two peaks, both of which had the desired mass. The reaction mixture was cooled to 23 ºC. The reaction mixture was partitioned between saturated aqueous sodium bicarbonate solution (1 mL) and dichloromethane (1 mL). The layers were separated, and the aqueous layer was extracted with dichloromethane (2 x 1 mL). The combined organic layers were dried over sodium sulfate. The dried solution was filtered, and the filtrate was concentrated. The residue was diluted with 50% acetonitrile–water to a volume of 4.7 mL, and the solution was filtered through a 0.45 µM PTFE syringe filter. The filtrate was purified by reverse-phase HPLC (Waters XBridge C18 column 5 µm particle size 30 x 250 mm, 5–95% acetonitrile–water + 0.1% formic acid, 40 min, 20 mL/min) to afford the product as a white solid (4.2 mg, 46%).

3:2 mixture of rotamers.
^{1}H NMR (400 MHz, CD_3OD) δ 8.34 (s, 1H), 5.31 – 5.09 (m, 2H), 4.97 (d, *J* = 9.9 Hz, 1H), 4.65 (s, 1H), 4.37 (d, *J* = 13.5 Hz, 1H), 4.12 – 3.89 (m, 2H), 3.80 – 3.70 (m, 1H), 3.70 – 3.59 (m, 2H), 3.59 – 3.47 (m, 2H), 3.44 (s, 3H), 3.42 (s, 3H), 3.40 – 3.37 (m, 3H), 3.36 (s, 3H), 3.09 – 2.98 (m, 2H), 2.90 (s, 6H), 2.89 – 2.84 (m, 2H), 2.68 – 2.60 (m, 2H), 2.42 – 2.27 (m, 3H), 2.27 – 2.13 (m, 3H), 2.13 – 1.76 (m, 6H), 1.76 – 1.67 (m, 6H), 1.68 – 1.29 (m, 8H), 1.16 – 1.03 (m, 2H), 1.01 – 0.87 (m, 9H). Number of protons: Expected: 82, Calculated from peak integration: 74.

HRMS (ESI): Calcd for $(C_{48}H_{80}N_2O_{12}S + H)^+$: 909.5510, Found: 909.5502

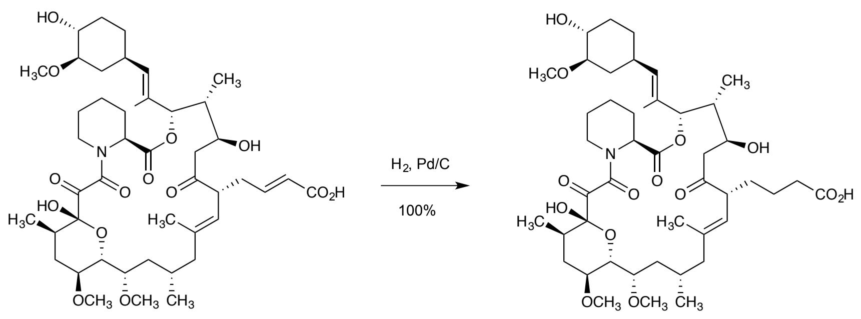

05-012 05-020

4-[(1R,9S,12S,13R,14S,17R,18E,21S,23S,24R,25S,27R)-1,14-dihydroxy-12-[(1E)-1-[(1R,3R,4R)-4-hydroxy-3-methoxycyclohexyl]prop-1-en-2-yl]-23,25-dimethoxy-13,19,21,27-tetramethyl-2,3,10,16-tetraoxo-11,28-dioxa-4-azatricyclo[22.3.1.$0^{4,9}$]octacos-18-en-17-yl]butanoic acid (**05-020**)

10 wt% Palladium on carbon (13 mg, 0.010 mmol) was added to a solution of 05-012 (50 mg, 0.060 mmol) in methanol (5 mL) at 23 ºC under an atmosphere of argon. The reaction flask was evacuated until effervescence, then flushed with hydrogen gas. The process was repeated three times. The resulting suspension was stirred at 23 ºC for 16 h under an atmosphere of hydrogen. The reaction flask was purged with argon, and the reaction suspension was filtered through a pad of Celite. The filter cake was rinsed with ethyl acetate (20 mL). The combined filtrate was concentrated in vacuo, and the residue was purified by column chromatography (0–10% methanol–dichloromethane + 0.1% acetic acid) to afford the product as a white solid (50 mg, 100%).

3:2 mixture of rotamers.
^{1}H NMR (400 MHz, Chloroform-*d*) δ 5.35 – 5.31 (m, 1H), 5.13 – 5.07 (m, 1H), 5.03 (d, *J* = 10.3 Hz, 1H), 4.61 (d, *J* = 5.4 Hz, 1H), 4.43 (d, *J* = 13.8 Hz, 1H), 4.01 – 3.89 (m, 1H), 3.70 (d, *J* = 9.6 Hz, 1H), 3.64 – 3.52 (m, 1H), 3.42 (s, 3H), 3.40 (s, 3H), 3.43 – 3.36 (m, 3H), 3.31 (s, 3H), 3.10 – 2.93 (m, 3H), 2.79 (dd, *J* = 15.9, 3.0 Hz, 1H), 2.45 – 2.25 (m, 5H), 2.26 – 2.10 (m, 3H), 2.09 – 1.88 (m, 6H), 1.89 – 1.71 (m, 6H), 1.60 (s, 6H), 1.60 – 1.35 (m, 8H), 1.13 – 1.03 (m, 2H), 1.00 (d, *J* = 6.3 Hz, 3H), 0.94 (d, *J* = 6.4 Hz, 3H), 0.88 (d, *J* = 7.1 Hz, 3H). Number of protons: Expected: 71, Calculated from peak integration: 69.
HRMS (ESI): Calcd for $(C_{45}H_{71}NO_{14} – H)^-$: 848.4796, Found: 848.4809

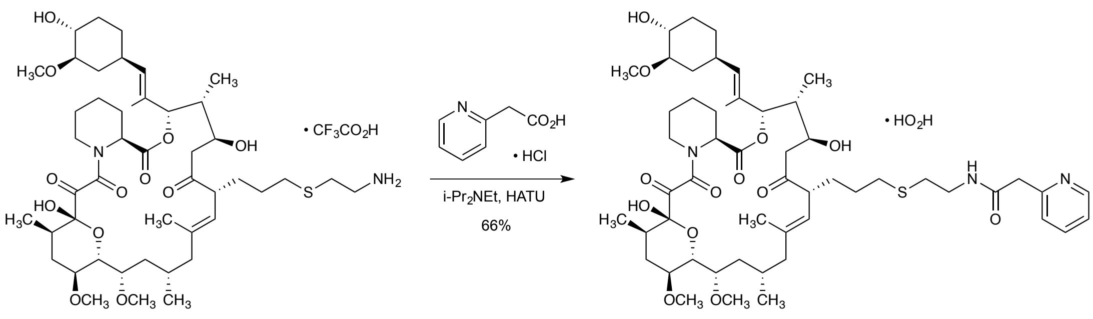

05-011 05-026

N-[2-({3-[(1R,9S,12S,13R,14S,17R,18E,21S,23S,24R,25S,27R)-1,14-dihydroxy-12-[(1E)-1-[(1R,3R,4R)-4-hydroxy-3-methoxycyclohexyl]prop-1-en-2-yl]-23,25-dimethoxy-13,19,21,27-tetramethyl-2,3,10,16-tetraoxo-11,28-dioxa-4-azatricyclo[22.3.1.0^{4,9}]octacos-18-en-17-yl]propyl}sulfanyl)ethyl]-2-(pyridin-2-yl)acetamide (**05-026**)

An oven-dried one-dram vial was charged with 05-011 (15 mg, 0.020 mmol), 2-(2-pyridyl)acetic acid hydrochloride (4.4 mg, 0.030 mmol), DMF (0.17 mL), N,N-Diisopropylethylamine (15 µL, 0.090 mmol), and a magnetic stir bar. HATU (7.7 mg, 0.020 mmol) was added as a solid at 23°C, and the resulting mixture was stirred at 23 °C for 30 min. At this point, LC-MS analysis showed full consumption of the starting amine and formation of a single peak corresponding to the desired mass. The residue was diluted with 50% acetonitrile–water to a volume of 5.0 mL, and the solution was filtered through a 0.45 µM PTFE syringe filter. The filtrate was purified by reverse-phase HPLC (Waters XBridge C18 column 5 µm particle size 30 x 250 mm, 5–95% acetonitrile–water + 0.1% formic acid, 40 min, 20 mL/min) to afford the product as a white solid (11.2 mg, 66%).

3:2 mixture of rotamers.
^{1}H NMR (400 MHz, Methanol-*d*$_4$) δ 8.54 – 8.45 (m, 1H), 8.13 (br s, 1H), 7.88 – 7.74 (m, 2H), 7.44 (d, *J* = 7.9 Hz, 1H), 7.37 – 7.31 (m, 1H), 5.29 – 5.08 (m, 2H), 4.93 (s, 1H), 4.63 (s, 1H), 4.36 (d, *J* = 13.4 Hz, 1H), 4.12 – 3.92 (m, 2H), 3.79 – 3.71 (m, 2H), 3.70 – 3.45 (m, 4H), 3.43 (s, 3H), 3.42 (s, 3H), 3.41 – 3.38 (m, 3H), 3.36 (s, 3H), 3.09 – 2.98 (m, 1H), 2.85 (dd, *J* = 14.4, 6.2 Hz, 1H), 2.64 (t, *J* = 7.0 Hz, 2H), 2.54 (t, *J* = 6.4 Hz, 2H), 2.44 – 2.27 (m, 4H), 2.27 – 2.08 (m, 4H), 2.08 – 1.74 (m, 6H), 1.74 – 1.64 (m, 6H), 1.64 – 1.33 (m, 8H), 1.18 – 1.01 (m, 2H), 0.99 – 0.86 (m, 9H). Number of protons: Expected: 83, Calculated from peak integration: 76.
HRMS (ESI): Calcd for $(C_{53}H_{81}N_3O_{13}S + H)^+$: 1000.5568, Found: 1000.5567

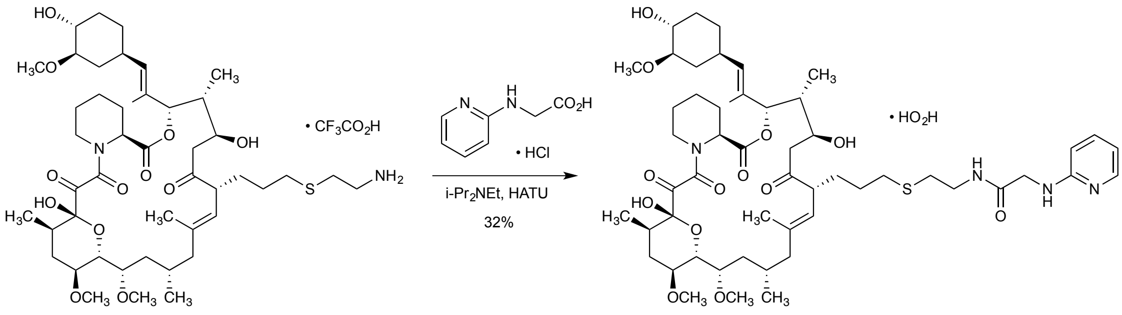

05-011 **05-028**

N-[2-({3-[(1R,9S,12S,13R,14S,17R,18E,21S,23S,24R,25S,27R)-1,14-dihydroxy-12-[(1E)-1-[(1R,3R,4R)-4-hydroxy-3-methoxycyclohexyl]prop-1-en-2-yl]-23,25-dimethoxy-13,19,21,27-tetramethyl-2,3,10,16-tetraoxo-11,28-dioxa-4-azatricyclo[22.3.1.0^{4,9}]octacos-18-en-17-yl]propyl}sulfanyl)ethyl]-2-[(pyridin-2-yl)amino]acetamide (**05-028**)

An oven-dried one-dram vial was charged with 05-011 (15 mg, 0.020 mmol), 2-(2-pyridylamino)acetic acid hydrochloride (4.8 mg, 0.030 mmol), DMF (0.17 mL), N,N-Diisopropylethylamine (15 µL, 0.090 mmol), and a magnetic stir bar. HATU (7.8 mg, 0.020 mmol) was added as a solid at 23°C, and the resulting mixture was stirred at 23 °C for 30 min. At this point, LC-MS analysis showed full consumption of the starting amine and formation of a single peak corresponding to the desired mass. The residue was diluted with 50% acetonitrile–water to a volume of 5.0 mL, and the solution was filtered through a 0.45 µM PTFE syringe filter. The filtrate was purified by reverse-phase HPLC (Waters XBridge C18 column 5 µm particle size 30 x 250 mm, 5–95% acetonitrile–water + 0.1% formic acid, 40 min, 20 mL/min) to afford the product as a white solid (5.5 mg, 32%).

3:2 mixture of rotamers.
HRMS (ESI): Calcd for $(C_{53}H_{82}N_4O_{13}S + H)^+$: 1015.5677, Found: 1015.5701

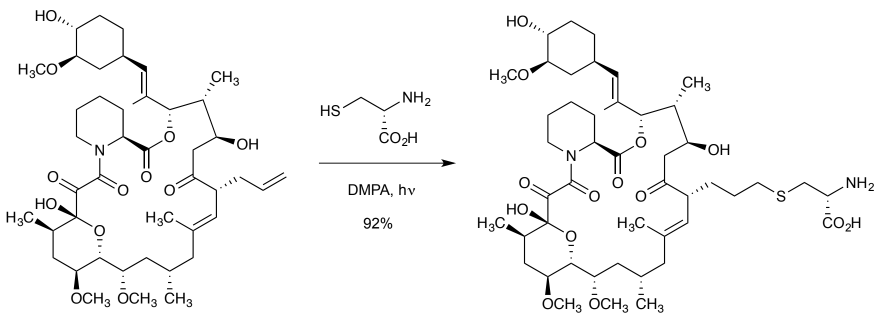

FK506 **05-037**

(2R)-2-amino-3-({3-[(1R,9S,12S,13R,14S,17R,18E,21S,23S,24R,25S,27R)-1,14-dihydroxy-12-[(1E)-1-[(1R,3R,4R)-4-hydroxy-3-methoxycyclohexyl]prop-1-en-2-yl]-23,25-dimethoxy-13,19,21,27-tetramethyl-2,3,10,16-tetraoxo-11,28-dioxa-4-azatricyclo[22.3.1.0^{4,9}]octacos-18-en-17-yl]propyl}sulfanyl)propanoic acid (**05-037**)

L-cysteine (16 mg, 0.13 mmol) and DMPA (3.2 mg, 0.012 mmol) were added sequentially to a solution of FK506 (100 mg, 0.12 mmol) in 1:1 Methanol (0.25 mL): Water (0.25 mL) at 23 ºC. After all reactants had dissolved, the vial was placed above a hand-held UV-light operating at 365 nm wavelength (the light was placed upside-down so that the contents of vial were directly irradiated). The irradiation was maintained for 15 min, at which point TLC analysis showed full disappearance of the starting material. The reaction solution was directly diluted with 50% acetonitrile–water to a volume of 4.7 mL, and the solution was filtered through a 0.45 μM PTFE syringe filter. The filtrate was purified by reverse-phase HPLC (Waters XBridge C18 column 5 μm particle size 30 x 250 mm, 5–95% acetonitrile–water + 0.1% formic acid, 40 min, 20 mL/min) to afford the product as a white solid (105 mg, 92%).

3:2 mixture of rotamers.

^{1}H NMR (400 MHz, Methanol-d_4) δ 5.31 – 5.10 (m, 2H), 4.94 (s, 1H), 4.63 (d, J = 7.9 Hz, 1H), 4.37 (d, J = 13.2 Hz, 1H), 4.10 – 3.94 (m, 1H), 3.78 – 3.69 (m, 1H), 3.67 – 3.45 (m, 3H), 3.43 (s, 3H), 3.42 (s, 3H), 3.41 – 3.39 (m, 3H), 3.36 (s, 3H), 3.15 (ddd, J = 14.7, 3.8, 1.2 Hz, 1H), 3.11 – 2.97 (m, 1H), 2.97 – 2.71 (m, 4H), 2.62 (t, J = 7.1 Hz, 1H), 2.43 – 2.28 (m, 4H), 2.28 – 2.11 (m, 4H), 2.09 – 1.75 (m, 6H), 1.75 – 1.68 (m, 6H), 1.68 – 1.28 (m, 8H), 1.21 – 1.00 (m, 2H), 1.00 – 0.86 (m, 9H). Number of protons: Expected: 76, Calculated from peak integration: 68.
HRMS (ESI): Calcd for $(C_{47}H_{76}N_2O_{14}S + H)^+$: 925.5095, Found: 925.5092.

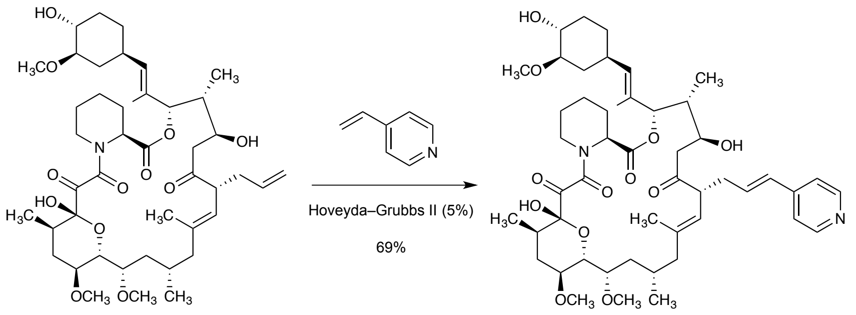

FK506 **05-051**

(1R,9S,12S,13R,14S,17R,18E,21S,23S,24R,25S,27R)-1,14-dihydroxy-12-[(1E)-1-[(1R,3R,4R)-4-hydroxy-3-methoxycyclohexyl]prop-1-en-2-yl]-23,25-dimethoxy-13,19,21,27-tetramethyl-17-[(2E)-3-(pyridin-4-yl)prop-2-en-1-yl]-11,28-dioxa-4-azatricyclo[22.3.1.$0^{4,9}$]octacos-18-ene-2,3,10,16-tetrone (**05-051**)

A 15-ml microwave vial was dried with gentle flame under vacuum. The vial was cooled to 23 °C, flushed with argon, then charged with FK506 (50 mg, 0.060 mmol), DCE (0.62 mL) and a magnetic stir bar. Argon was bubbled through the resulting solution via a 19-gauge needle for 1 min. 4-Vinylpyridine (6.7 µL, 0.060 mmol) was added via pipette, and Grubbs-Hoveyda 2nd Gen Catalyst (4.0 mg, 0.0062 mmol) was added in one portion as a solid. The mixture was stirred briefly (giving a bright green solution) before being loaded on a CEM DiscoverSP microwave reactor. Microwave reaction was performed at 100 °C for 30 min with 1 min pre-equilibration. After cooling to 23 °C, the reaction mixture was analyzed by TLC (100% ethyl acetate), which showed formation of an UV-active, more polar spot. The reaction mixture was directly loaded onto a 4-g RediSep (Teledyne ISCO) column. Elution with 100% ethyl acetate gave the product as a yellow solid (38 mg, 69%).

3:2 mixture of rotamers.
^{1}H NMR (400 MHz, Chloroform-*d*) δ 8.56 – 8.49 (m, 2H), 8.21 (br s, 1H), 7.27 – 7.18 (m, 2H), 6.49 – 6.32 (m, 2H), 5.35 (s, 1H), 5.17 – 5.05 (m, 2H), 4.67 (d, *J* = 5.5 Hz, 1H), 4.46 (d, *J* = 13.7 Hz, 1H), 4.04 – 3.86 (m, 1H), 3.72 (d, *J* = 9.6 Hz, 1H), 3.61 (d, *J* = 10.6 Hz, 1H), 3.56 – 3.45 (m, 1H), 3.43 (s, 3H), 3.41 (s, 3H), 3.39 – 3.36 (m, 2H), 3.32 (s, 3H), 3.08 – 2.98 (m, 3H), 2.84 (dd, *J* = 16.3, 2.6 Hz, 1H), 2.80 – 2.60 (m, 2H), 2.55 – 2.25 (m, 4H), 2.26 – 1.88 (m, 6H), 1.88 – 1.71 (m, 4H), 1.71 – 1.61 (m, 6H), 1.61 – 1.26 (m, 8H), 1.14 – 1.04 (m, 2H), 1.01 (d, *J* = 6.3 Hz, 3H), 0.96 (d, *J* = 6.4 Hz, 3H), 0.90 (d, *J* = 7.2 Hz, 3H). Number of protons: Expected: 72, Calculated from peak integration: 69.
HRMS (ESI): Calcd for $(C_{49}H_{72}N_2O_{12} + H)^+$: 881.5163, Found: 881.5207

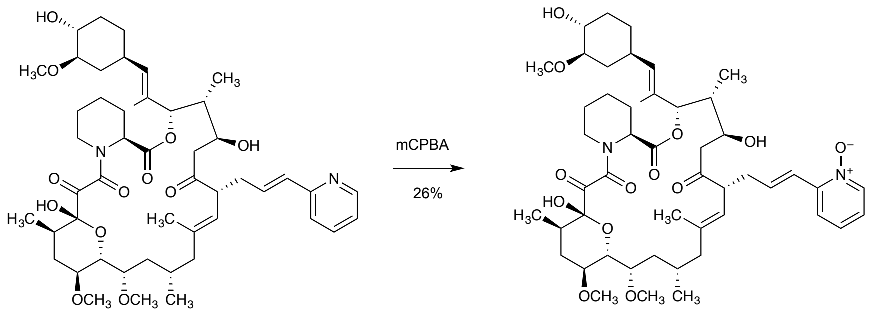

05-050 05-060

2-[(1E)-3-[(1R,9S,12S,13R,14S,17R,18E,21S,23S,24R,25S,27R)-1,14-dihydroxy-12-[(1E)-1-[(1R,3R,4R)-4-hydroxy-3-methoxycyclohexyl]prop-1-en-2-yl]-23,25-dimethoxy-13,19,21,27-tetramethyl-2,3,10,16-tetraoxo-11,28-dioxa-4-azatricyclo[22.3.1.$0^{4,9}$]octacos-18-en-17-yl]prop-1-en-1-yl]-1-methylpyridin-1-ium (**05-060**)

*m*CPBA (3.4 mg, 0.010 mmol) was added as a 10 wt% solution in dichloromethane (34 μL) to a solution of 05-050 (10 mg, 0.010 mmol) in dichloromethane (0.11 mL) at 0 ºC. In 4 h, most of the starting material had been consumed judged by TLC analysis (100% ethyl acetate). The reaction mixture was partitioned between dichloromethane (5 mL) and saturated sodium bicarbonate solution (5 mL). The layers were separated, and the aqueous layer was extracted with dichloromethane (2 x 5 mL). The combined organic layers were dried over sodium sulfate, and the dried solution was filtered and concentrated. Purification by column chromatography (0-10% methanol-dichloromethane) afforded a white solid. The material was further again by reverse-phase HPLC (5–95% acetonitrile-water + 0.1% formic acid over 40 min). The product-containing fraction were pooled and concentrated *in vacuo* to afford the product as a while solid (2.6 mg, 26%).

3:2 mixture of rotamers.
^{1}H NMR (400 MHz, Chloroform-*d*) δ 8.14 – 8.08 (m, 2H), 7.22 – 7.10 (m, 2H), 6.38 – 6.17 (m, 2H), 5.34 (s, 1H), 5.14 – 5.03 (m, 2H), 4.65 (d, *J* = 6.0 Hz, 1H), 4.44 (d, *J* = 13.4 Hz, 1H), 4.04 – 3.84 (m, 2H), 3.69 (d, *J* = 9.6 Hz, 1H), 3.68 – 3.52 (m, 2H), 3.41 (s, 3H), 3.39 (s, 3H), 3.38 – 3.34 (m, 3H), 3.30 (s, 3H), 3.08 – 2.95 (m, 3H), 2.81 (d, *J* = 16.1 Hz, 1H), 2.75 – 2.58 (m, 2H), 2.52 – 2.21 (m, 4H), 2.21 – 1.72 (m, 6H), 1.72 – 1.52 (m, 6H), 1.53 – 1.13 (m, 8H), 1.12 – 1.02 (m, 2H), 0.99 (d, *J* = 6.3 Hz, 3H), 0.94 (d, *J* = 6.4 Hz, 3H), 0.87 (d, *J* = 7.2 Hz, 3H). Number of protons: Expected: 72, Calculated from peak integration: 69.
HRMS (ESI): Calcd for $(C_{49}H_{72}N_2O_{13} + H)^+$: 897.5112, Found: 897.5134

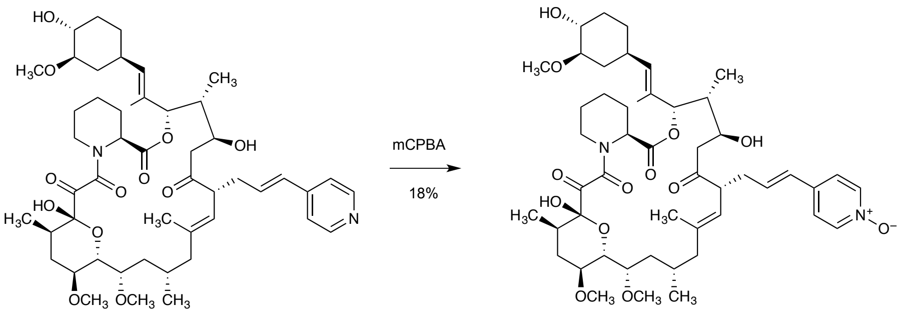

05-051 **05-061**

4-[(1E)-3-[(1R,9S,12S,13R,14S,17R,18E,21S,23S,24R,25S,27R)-1,14-dihydroxy-12-[(1E)-1-[(1R,3R,4R)-4-hydroxy-3-methoxycyclohexyl]prop-1-en-2-yl]-23,25-dimethoxy-13,19,21,27-tetramethyl-2,3,10,16-tetraoxo-11,28-dioxa-4-azatricyclo[22.3.1.$0^{4,9}$]octacos-18-en-17-yl]prop-1-en-1-yl]-1-methylpyridin-1-ium (**05-061**)

*m*CPBA (3.4 mg, 0.010 mmol) was added as a 10 wt% solution in dichloromethane (34 uL) to a solution of 05-051 (10 mg, 0.010 mmol) in dichloromethane (0.11 mL) at 0 ºC. In 4 h, most of the starting material had been consumed judged by TLC analysis (100% ethyl acetate. The reaction mixture was partitioned between dichloromethane (5 mL) and saturated sodium bicarbonate solution (5 mL). The layers were separated, and the aqueous layer was extracted with dichloromethane (2 x 5 mL). The combined organic layers were dried over sodium sulfate, and the dried solution was filtered and concentrated. Purification by column chromatography (0-10% methanol-dichloromethane) afforded a white solid. The material was further purified by reverse-phase HPLC (5–95% acetonitrile-water + 0.1% formic acid over 40 min). The product-containing fraction were pooled and concentrated *in vacuo* to afford the product as a while solid (1.8 mg, 18%).

3:2 mixture of rotamers.
^{1}H NMR (400 MHz, Chloroform-*d*) δ 8.19 (d, *J* = 5.8 Hz, 1H), 7.47 – 7.37 (m, 1H), 7.26 – 7.08 (m, 2H), 7.08 – 6.93 (m, 1H), 6.54 (dd, *J* = 15.7, 7.8 Hz, 1H), 5.29 (s, 1H), 5.17 – 5.07 (m, 2H), 4.55 – 4.27 (m, 2H), 4.27 – 4.13 (m, 1H), 3.80 – 3.49 (m, 3H), 3.41 (s, 3H), 3.38 (s, 3H), 3.37 – 3.32 (m, 3H), 3.29 (s, 3H), 3.10 – 2.93 (m, 3H), 2.84 (d, *J* = 16.9 Hz, 1H), 2.75 – 2.55 (m, 2H), 2.50 – 2.23 (m, 4H), 2.25 – 1.79 (m, 6H), 1.75 – 1.64 (m, 6H), 1.54 – 1.27 (m, 8H), 1.07 – 0.98 (m, 2H), 0.96 (d, *J* = 7.1 Hz, 3H), 0.92 (d, *J* = 6.9 Hz, 3H), 0.89 (d, *J* = 4.6 Hz, 3H). Number of protons: Expected: 72, Calculated from peak integration: 68.

HRMS (ESI): Calcd for $(C_{49}H_{72}N_2O_{13} + H)^+$: 897.5112, Found: 897.5134

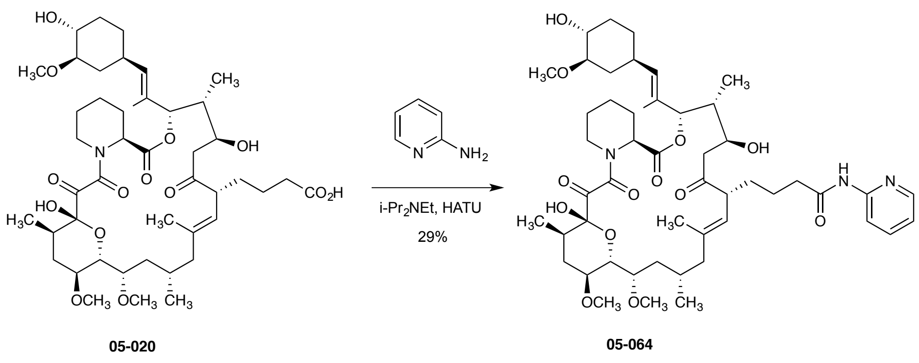

4-[(1R,9S,12S,13R,14S,17R,18E,21S,23S,24R,25S,27R)-1,14-dihydroxy-12-[(1E)-1-[(1R,3R,4R)-4-hydroxy-3-methoxycyclohexyl]prop-1-en-2-yl]-23,25-dimethoxy-13,19,21,27-tetramethyl-2,3,10,16-tetraoxo-11,28-dioxa-4-azatricyclo[22.3.1.0^{4,9}]octacos-18-en-17-yl]-N-(pyridin-2-yl)butanamide (**05-064**)

N,*N*-Diisopropylethylamine (12.3 µL, 0.071 mmol) and HATU (11 mg, 0.028 mmol) were added sequentially to a stirred solution of 05-020 (20 mg, 0.024 mmol) and 2-aminopyridine (2.7 mg, 0.028 mmol) in DMF (0.24 mL) at 23 °C. In 24 h, LC-MS analysis showed full consumption of the staring acid. The reaction mixture was diluted with 50% acetonitrile–water to a volume of 4.5 mL, and the solution was filtered through a 0.45 µM PTFE syringe filter. The filtrate was purified by reverse-phase HPLC (Waters XBridge C18 column 5 µm particle size 30 x 250 mm, 5–95% acetonitrile–water + 0.1% formic acid, 40 min, 20 mL/min) to afford the product as a white solid (6.4 mg, 29%).

3:2 mixture of rotamers.

^{1}H NMR (400 MHz, Chloroform-*d*) δ 9.18 (br s, 1H), 8.29 (d, *J* = 8.6 Hz, 1H), 8.24 – 8.16 (m, 1H), 7.79 – 7.72 (m, 1H), 7.09 – 7.04 (m, 1H), 5.35 (d, *J* = 2.5 Hz, 1H), 5.14 – 4.98 (m, 2H), 4.65 (d, *J* = 5.4 Hz, 1H), 4.45 (d, *J* = 13.8 Hz, 1H), 4.03 – 3.84 (m, 2H), 3.81 – 3.68 (m, 1H), 3.68 – 3.55 (m, 2H), 3.43 (s, 3H), 3.41 (s, 3H), 3.41 – 3.36 (m, 3H), 3.32 (s, 3H), 3.13 – 2.93 (m, 2H), 2.81 (dd, *J* = 16.2, 2.2 Hz, 1H), 2.51 – 2.25 (m, 6H), 2.25 – 1.72 (m, 8H), 1.71 – 1.58 (m, 6H), 1.57 – 1.19 (m, 8H), 1.18 – 1.05 (m, 2H), 1.02 (d, *J* = 6.2 Hz, 3H), 0.96 (d, *J* = 6.4 Hz, 3H), 0.89 (d, *J* = 7.2 Hz, 3H). Number of protons: Expected: 75, Calculated from peak integration: 69.

HRMS (ESI): Calcd for $(C_{50}H_{75}N_3O_{13} + H)^+$: 926.5378, Found: 926.5380

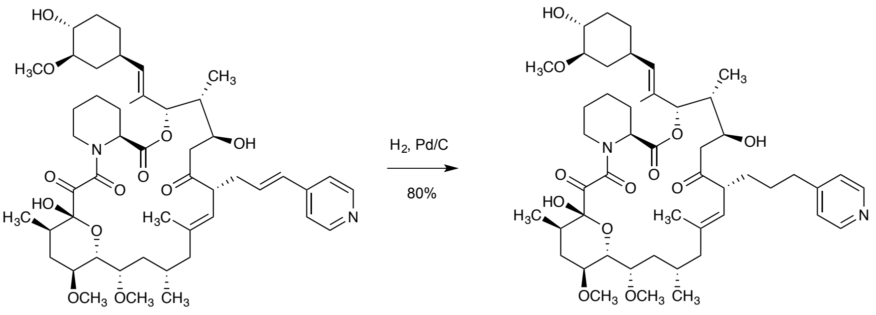

05-051 **05-084 (06-039)**

(1R,9S,12S,13R,14S,17R,18E,21S,23S,24R,25S,27R)-1,14-dihydroxy-12-[(1E)-1-[(1R,3R,4R)-4-hydroxy-3-methoxycyclohexyl]prop-1-en-2-yl]-23,25-dimethoxy-13,19,21,27-tetramethyl-17-[3-(pyridin-4-yl)propyl]-11,28-dioxa-4-azatricyclo[22.3.1.0^{4,9}]octacos-18-ene-2,3,10,16-tetrone (**05-084/06-039)**

A 20-mL vial was charged with 05-051 (20 mg, 0.020 mmol), Ethyl acetate (1.0 mL) and Palladium on carbon (10 wt%, 2.4 mg). The vial was briefly purged with argon, and then fitted with a rubber septum. Hydrogen was bubbled through the solution via a 19-gauge needle for 5 min, then the mixture was stirred under hydrogen atmosphere at 23 ºC. In a total of 3 h, LC-MS showed full conversion to the desired product. The reaction flask was evacuated and refilled with argon. This cycle was repeated for a total of three times. Afterwards, the reaction mixture was filtered through a pad of Celite under a blanket of argon, and the filter cake was rinsed with ethyl acetate (5 mL). CAUTION: palladium on carbon is extremely flammable after a hydrogenation reaction; care must be taken not to allow the filter cake to dry. The combined filtrate was concentrated to afford the product (**05-084**) as a pale-yellow foam (16 mg, 80%).

3:2 mixture of rotamers.
^{1}H NMR (400 MHz, Chloroform-*d*) δ 8.53 – 8.47 (m, 2H), 7.13 – 7.07 (m, 2H), 5.34 (d, *J* = 2.7 Hz, 1H), 5.13 – 4.97 (m, 2H), 4.63 (d, *J* = 5.4 Hz, 1H), 4.45 (d, *J* = 13.7 Hz, 2H), 4.00 – 3.86 (m, 2H), 3.78 – 3.66 (m, 1H), 3.66 – 3.55 (m, 1H), 3.55 – 3.45 (m, 1H), 3.43 (s, 3H), 3.41 (s, 3H), 3.40 – 3.38 (m, 2H), 3.32 (s, 3H), 3.10 – 2.98 (m, 2H), 2.78 (dd, *J* = 15.9, 2.9 Hz, 1H), 2.62 (app t, *J* = 7.7 Hz, 2H), 2.40 – 2.24 (m, 3H), 2.24 – 2.13 (m, 3H), 2.06 (s, 6H), 1.86 – 1.71 (m, 4H), 1.71 – 1.57 (m, 6H), 1.57 – 1.33 (m, 8H), 1.15 – 1.05 (m, 2H), 1.02 (d, *J* = 6.3 Hz, 3H), 0.95 (d, *J* = 6.4 Hz, 3H), 0.87 (d, *J* = 7.1 Hz, 3H). Number of protons: Expected: 74, Calculated from peak integration: 71.
HRMS (ESI): Calcd for $(C_{49}H_{74}N_2O_{12} + H)^+$: 883.5320, Found: 883.5332

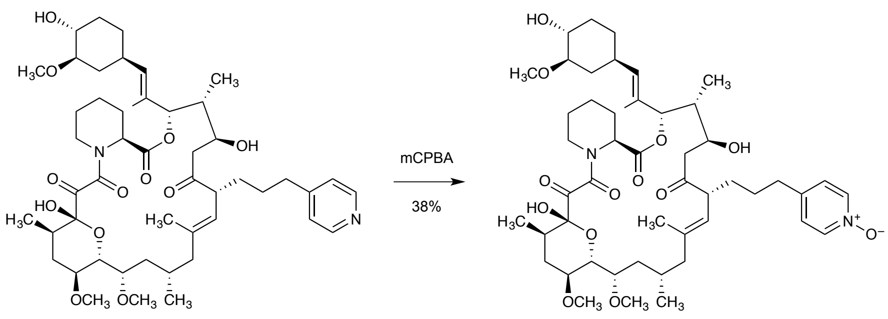

05-084 **05-086**

(1R,9S,12S,13R,14S,17R,18E,21S,23S,24R,25S,27R)-1,14-dihydroxy-12-[(1E)-1-[(1R,3R,4R)-4-hydroxy-3-methoxycyclohexyl]prop-1-en-2-yl]-23,25-dimethoxy-13,19,21,27-tetramethyl-17-[3-(pyridin-2-yl)propyl]-11,28-dioxa-4-azatricyclo[22.3.1.$0^{4,9}$]octacos-18-ene-2,3,10,16-tetrone (**05-086**)

m-CPBA (4.8 mg, 0.018 mmol) was added as a 10 wt% solution in dichloromethane (48 µL) to a solution of 05-084 (16 mg, 0.018 mmol) in dichloromethane at 0 ºC. The reaction progress was monitored by LC-MS. In a total of 6 h, LC-MS showed full conversion to the desired m/z. The reaction mixture was directly concentrated under reduced pressure. The residue was diluted with 50% acetonitrile–water to a volume of 3.0 mL, and the solution was filtered through a 0.45 µM PTFE syringe filter. The filtrate was purified by reverse-phase HPLC (Waters XBridge C18 column 5 µm particle size 30 x 250 mm, 50–95% acetonitrile–water + 0.1% formic acid, 40 min, 20 mL/min) to afford the product as a white solid (6.2 mg, 38%).

3:2 mixture of rotamers.
^{1}H NMR (400 MHz, Chloroform-*d*) δ 8.24 – 8.14 (m, 2H), 7.13 (t, *J* = 6.8 Hz, 2H), 5.35 (s, 1H), 5.13 – 4.96 (m, 3H), 4.65 (d, *J* = 5.4 Hz, 1H), 4.45 (d, *J* = 13.6 Hz, 1H), 3.95 – 3.84 (m, 1H), 3.74 – 3.67 (m, 1H), 3.66 – 3.54 (m, 1H), 3.43 (s, 3H), 3.41 (s, 3H), 3.32 (s, 3H), 3.45 – 3.27 (m, 3H), 3.11 – 2.97 (m, 3H), 2.79 (dd, *J* = 16.2, 2.7 Hz, 1H), 2.67 – 2.59 (m, 2H), 2.31 (d, *J* = 9.4 Hz, 2H), 2.25 – 2.10 (m, 3H), 2.10 – 1.96 (m, 2H), 1.96 – 1.73 (m, 6H), 1.72 – 1.57 (m, 6H), 1.57 – 1.19 (m, 8H), 1.15 – 1.05 (m, 2H), 1.03 (d, *J* = 6.3 Hz, 3H), 0.96 (d, *J* = 6.3 Hz, 4H), 0.88 (d, *J* = 7.2 Hz, 3H). Number of protons: Expected: 74, Calculated from peak integration: 71.
HRMS (ESI): Calcd for $(C_{49}H_{74}N_2O_{13} + H - H_2O)^+$: 881.5164, Found: 881.5146

Synthesis of FK-GNE7915

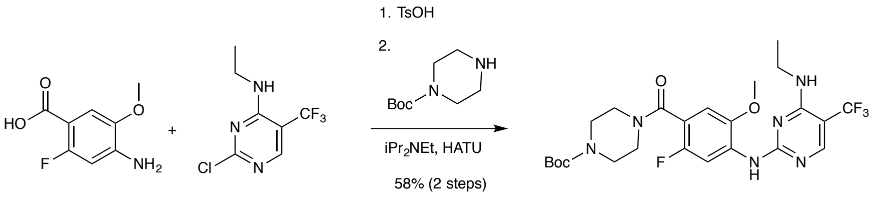

tert-butyl 4-(4-((4-(ethylamino)-5-(trifluoromethyl)pyrimidin-2-yl)amino)-2-fluoro-5-methoxybenzoyl)piperazine-1-carboxylate (**S7**)

A 20-mL vial was charged with 4-amino-2-fluoro-5-methoxy-benzoic acid (100 mg, 0.54 mmol), 2-chloro-N-ethyl-5-(trifluoromethyl)pyrimidin-4-amine (146 mg, 0.650 mmol), p-toluenesulfonic acid monohydrate (51 mg, 0.27 mmol) and 1,4-dioxane (8.1 mL). The mixture was heated to 100 ºC with constant stirring. Despite heating not all the solids dissolved. After 2 h, LC-MS analysis showed full conversion to the desired product. The reaction mixture was then cooled to room temperature. The insoluble solids were collected by filtration, washed with 1,4-dioxane (100 mL) and ethyl (50 mL), and air-dried for 12 h to afford the product as a white solid. The crude material was used in the next step without further purification.

N-Boc piperazine (199 mg, 1.07 mmol), dichloromethane (2.67 mL), and N,N-diisopropylamine (93 µL, 0.53 mmol) were added to the crude product from the last reaction. The resulting suspension was cooled to 0 ºC, and HATU (308 mg, 0.802 mmol) was added in one portion. The mixture was stirred at 0 ºC for 15 min before warming to 23 ºC and stirring for another 45 min. The reaction mixture was partitioned between saturated aqueous sodium bicarbonate solution (5 mL) and dichloromethane (5 mL). The layers were separated, and the aqueous layer was extracted with dichloromethane (2 x 5 mL). The combined organic layers were dried over sodium sulfate. The dried solution was filtered, and the filtrate was concentrated. The residue was purified by column chromatography (20–50% ethyl acetate–hexanes, 4-g RediSep(R) Rf column, Teledyne ISCO, Lincoln, NE) to afford the product (**S7**) as a white powder (151 mg, 58% over 2 steps).

^{1}H NMR (400 MHz, Chloroform-*d*) δ 8.41 (br s, 1H), 8.18 (s, 1H), 6.91 (d, *J* = 6.0 Hz, 1H), 3.92 (s, 3H), 3.76 (s, 2H), 3.68 – 3.58 (m, 2H), 3.58 – 3.47 (m, 2H), 3.49 – 3.35 (m, 4H), 1.48 (s, 9H), 1.34 (t, *J* = 7.2 Hz, 3H). Number of protons: Expected: 30, Calculated from peak integration: 28.

HRMS (ESI): Calcd for $(C_{24}H_{30}F_4N_6O_4 + H)^+$: 543.2343, Found: 543.2389.

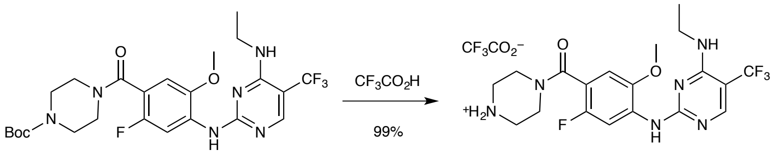

S7 S8

4-(4-((4-(ethylamino)-5-(trifluoromethyl)pyrimidin-2-yl)amino)-2-fluoro-5-methoxybenzoyl)piperazin-1-ium trifluoroacetate (**S8**)

Trifluoroacetic acid (0.50 mL) was added dropwise to a solution of **S7** (151 mg, 0.28 mmol) in dichloromethane (0.50 mL) at 23 ºC and the resulting solution was allowed to stand at 23 ºC for 1 h. The reaction mixture was concentrated in vacuo to afford the product as a white solid. To assist removal of residual trifluoroacetic acid, the solids were triturated with ether (10 mL), and the supernatant was removed. The resulting solids were dried under vacuum over night to afford the product (**S8**) as a white powder (153 mg, 99%).

^{1}H NMR (400 MHz, Methanol-d_4) δ 8.38 (d, J = 11.9 Hz, 1H), 8.29 (d, J = 1.1 Hz, 1H), 7.14 (d, J = 6.1 Hz, 1H), 4.08 – 4.01 (m, 2H), 4.00 (s, 3H), 3.78 – 3.69 (m, 2H), 3.66 (q, J = 7.2 Hz, 2H), 3.41 – 3.25 (m, 4H), 1.32 (t, J = 7.1 Hz, 3H). Number of protons: Expected: 22, Calculated from peak integration: 19.

HRMS (ESI): Calcd for $(C_{19}H_{21}F_4N_6O_2 + H)^+$: 443.1813, Found: 443.1786

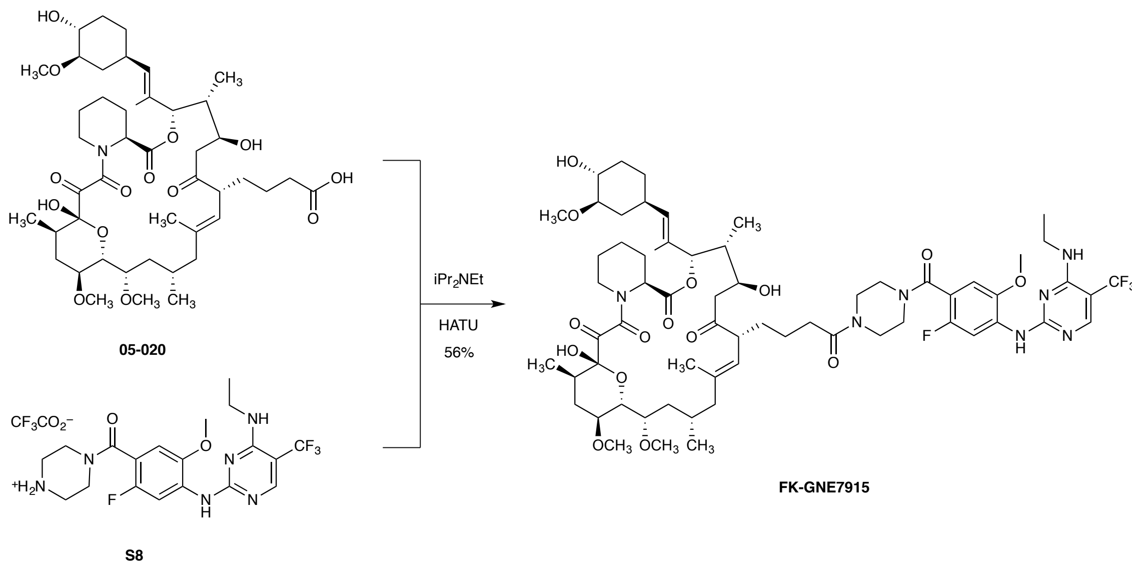

(1R,9S,12S,13R,14S,17R,18E,21S,23S,24R,25S,27R)-17-{4-[4-(4-{[4-(ethylamino)-5-(trifluoromethyl)pyrimidin-2-yl]amino}-2-fluoro-5-methoxybenzoyl)piperazin-1-yl]-4-oxobutyl}-1,14-dihydroxy-12-[(1E)-1-[(1R,3R,4R)-4-hydroxy-3-methoxycyclohexyl]prop-1-en-2-yl]-23,25-dimethoxy-13,19,21,27-tetramethyl-11,28-dioxa-4-azatricyclo[22.3.1.$0^{4,9}$]octacos-18-ene-2,3,10,16-tetrone (**FK-GNE7915**)

An oven-dried 1-dram vial was charged with **05-020** (20 mg, 0.024 mmol), **S8** (13 mg, 0.023 mmol), DMF (0.12 mL) and a magnetic stir bar. *N*,*N*-Diisopropylethylamine (12 µL, 0.071 mmol) was added and the mixture was stirred until all reactants had dissolved. HATU (11 mg, 0.030 mmol) was added as a 10% (w/v) solution in DMF (110 µL), and the reaction progress was monitored by LC-MS. In 30 min, LC-MS analysis showed that the FK506-acid starting material had been fully consumed and a new product with desired m/z had formed. The reaction mixture was partitioned between saturated aqueous sodium bicarbonate solution (5 mL) and dichloromethane (5 mL). The layers were separated, and the aqueous layer was extracted with dichloromethane (2 x 5 mL). The combined organic layers were dried over sodium sulfate. The dried solution was filtered, and the filtrate was concentrated. The residue was purified by column chromatography (0–10% methanol–dichloromethane, 4-g RediSep(R) Rf column, Teledyne ISCO, Lincoln, NE) to afford the product (**FK-GNE7915**) as a white powder (16.7 mg, 56%).

3:2 mixture of rotamers.
^{1}H NMR (400 MHz, Chloroform-*d*) δ 8.45 (d, *J* = 12.4 Hz, 1H), 8.20 (s, 1H), 7.87 (s, 1H), 6.91 (s, 1H), 5.34 (s, 1H), 5.26 – 5.16 (m, 1H), 5.16 – 4.92 (m, 2H), 4.61 (d, *J* = 5.4 Hz, 1H), 4.42 (d, *J* = 13.4 Hz, 1H), 3.92 (s, 3H), 3.85 – 3.65 (m, 5H), 3.65 – 3.51 (m, 5H), 3.51 – 3.43 (m, 3H), 3.40 (s, 3H), 3.38 (s, 3H), 3.43 – 3.31 (m, 3H), 3.29 (s, 3H), 3.24 – 3.19 (m, 1H), 3.19 – 3.12 (m, 1H), 3.05 – 2.96 (m, 2H), 2.79 (dd, *J* = 15.9, 2.4 Hz, 1H), 2.67

(br s, 1H), 2.43 – 2.22 (m, 5H), 2.22 – 1.94 (m, 5H), 1.94 – 1.69 (m, 6H), 1.69 – 1.60 (m, 6H), 1.60 – 1.40 (m, 8H), 1.33 (t, J = 7.2 Hz, 3H), 1.10 – 1.02 (m, 2H), 0.99 (d, J = 6.3 Hz, 3H), 0.93 (d, J = 5.9 Hz, 3H), 0.85 (d, J = 7.6 Hz, 3H). Number of protons: Expected: 91, Calculated from peak integration: 88.

HRMS (ESI): Calcd for $(C_{64}H_{91}F_4N_7O_{15} + H)^+$: 1274.6587, Found: 1274.6560.

Synthesis of FK-Dasatinib

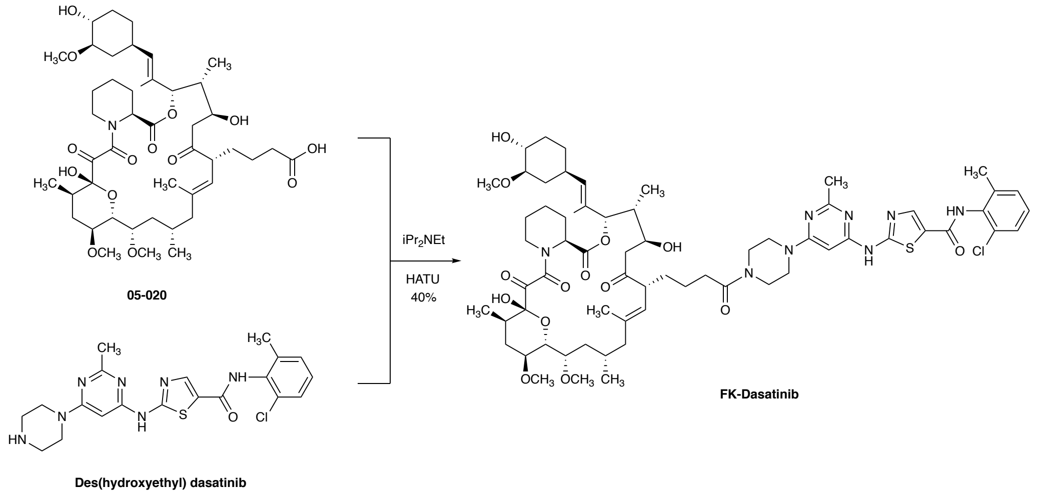

N-(2-chloro-6-methylphenyl)-2-{[6-(4-{4-[(1R,9S,12S,13R,14S,17R,18E,21S,23S,24R,25S,27R)-1,14-dihydroxy-12-[(1E)-1-[(1R,3R,4R)-4-hydroxy-3-methoxycyclohexyl]prop-1-en-2-yl]-23,25-dimethoxy-13,19,21,27-tetramethyl-2,3,10,16-tetraoxo-11,28-dioxa-4-azatricyclo[22.3.1.0^{4,9}]octacos-18-en-17-yl]butanoyl}piperazin-1-yl)-2-methylpyrimidin-4-yl]amino}-1,3-thiazole-5-carboxamide (**FK-Dasatinib**)

An oven-dried 1-dram vial was charged with **05-020** (10 mg, 0.012 mmol), des(hydroxyethyl)dasatinib (5.8 mg, 0.012 mmol), DMF (0.20 mL) and a magnetic stir bar. The solution was cooled to 0 ºC, then *N*,*N*-diisopropylethylamine (6.2 µL, 0.035 mmol) and HATU (5.4 mg, 0.010 mmol) were added sequentially. The resulting mixture was stirred at 0 ºC and the reaction progress was monitored by LC-MS. In 30 min, LC-MS analysis indicated that the starting material had been fully consumed. The reaction mixture was diluted with 50% acetonitrile–water to a volume of 5.0 mL, and the solution was filtered through a 0.45 µM PTFE syringe filter. The filtrate was purified by reverse-phase HPLC (Waters XBridge C18 column 5 µm particle size 30 x 250 mm, 5–95% acetonitrile–water + 0.1% formic acid, 40 min, 20 mL/min) to afford the product as a white solid (5.9 mg, 40%).

3:2 mixture of rotamers.
^{1}H NMR (400 MHz, Chloroform-*d*) δ 8.19 (s, 1H), 7.99 (br s, 1H), 7.42 – 7.31 (m, 2H), 7.26 – 7.10 (m, 2H), 5.89 (s, 1H), 5.39 (s, 1H), 5.16 – 5.06 (m, 1H), 4.66 (d, *J* = 5.4 Hz, 1H), 4.48 (d, *J* = 13.8 Hz, 1H), 3.99 – 3.94 (m, 1H), 3.81 – 3.68 (m, 5H), 3.68 – 3.53 (m, 5H), 3.44 (s, 3H), 3.41 (s, 3H), 3.46 – 3.33 (m, 3H), 3.32 (s, 3H), 3.10 – 2.98 (m, 3H), 2.79 (d, *J* = 14.7 Hz, 1H), 2.55 (s, 3H), 2.43 – 2.38 (m, 1H), 2.37 (s, 3H), 2.35 – 2.25 (m, 2H), 2.24 – 1.98 (m, 5H), 1.97 – 1.72 (m, 6H), 1.71 – 1.62 (m, 6H), 1.62 – 1.32 (m, 8H),

1.12 – 1.04 (m, 2H), 1.02 (d, *J* = 6.2 Hz, 3H), 0.95 (d, *J* = 5.6 Hz, 3H), 0.89 (d, *J* = 7.2 Hz, 3H). Number of protons: Expected: 91, Calculated from peak integration: 83.
HRMS (ESI): Calcd for $(C_{65}H_{91}ClN_8O_{14}S + H)^+$: 1275.6142, Found: 1275.6085.

Synthesis of FK-Lapatinib

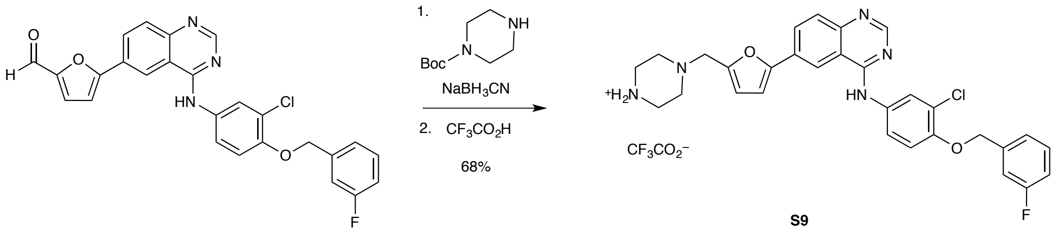

4-((5-(4-((3-chloro-4-((3-fluorobenzyl)oxy)phenyl)amino)quinazolin-6-yl)furan-2-yl)methyl)piperazin-1-ium trifluoroacetate (**S9**)

A suspension of 5-[4-[3-chloro-4-[(3-fluorophenyl)methoxy]anilino]quinazolin-6-yl]furan-2-carbaldehyde (100 mg, 0.211 mmol) in 9:1 methanol (1.8 mL):acetic acid (0.2 mL) was sonicated until a fine suspension was formed. 1-Boc-Piperazine (79 mg, 0.42 mmol) was added and the resulting mixture was stirred at 23 ºC for 30 min. Sodium cyanoborohydride (20 mg, 0.32 mmol) was added in a single portion at 23 ºC. The precipitates dissolved over time to a point with a few speckles left in 1 h. At this point TLC analysis (100% ethyl acetate) showed full consumption of the aldehyde starting material. The reaction mixture was concentrated under reduced pressure. The reaction mixture was partitioned between saturated aqueous sodium bicarbonate solution (5 mL) and dichloromethane (5 mL). The layers were separated, and the aqueous layer was extracted with dichloromethane (2 x 5 mL). The combined organic layers were dried over sodium sulfate. The dried solution was filtered, and the filtrate was concentrated. The residue was purified by column chromatography (20–100% ethyl acetate–hexanes) to afford the product as a yellow powder.

The yellow powder was resuspended in dichloromethane (2.0 mL), and trifluoroacetic acid (2.0 mL) was added dropwise, giving rise to a bright yellow solution. After standing at 23 ºC for 1 h, the solution was concentrated under reduced pressure to afford the product as a yellow powder (93 mg, 68%).

HRMS (ESI): Calcd for $(C_{30}H_{27}ClFN_5O_2 + H)^+$: 544.1910, Found: 544.1882.

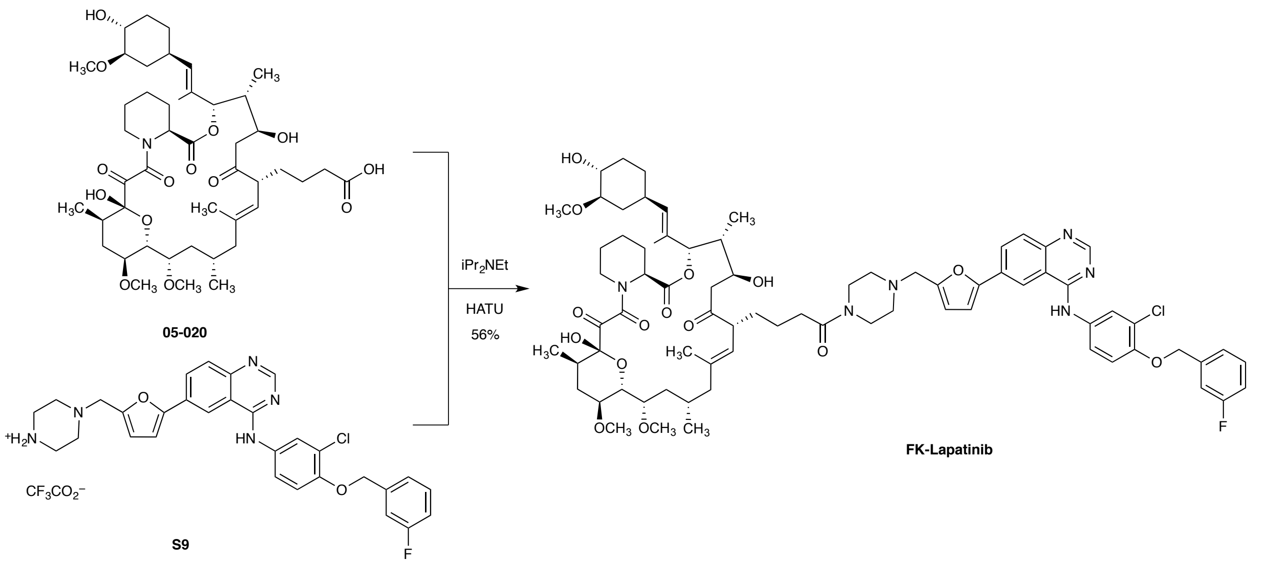

(1R,9S,12S,13R,14S,17R,18E,21S,23S,24R,25S,27R)-17-{4-[4-({5-[4-({3-chloro-4-[(3-fluorophenyl)methoxy]phenyl}amino)quinazolin-6-yl]furan-2-yl}methyl)piperazin-1-yl]-4-oxobutyl}-1,14-dihydroxy-12-[(1E)-1-[(1R,3R,4R)-4-hydroxy-3-methoxycyclohexyl]prop-1-en-2-yl]-23,25-dimethoxy-13,19,21,27-tetramethyl-11,28-dioxa-4-azatricyclo[22.3.1.0^{4,9}]octacos-18-ene-2,3,10,16-tetrone (**FK-Lapatinib**)

N,*N*-Diisopropylethylamine (12.3 µL, 0.071 mmol) and HATU (9.8 mg, 0.026 mmol) were added sequentially to a stirred solution of **05-020** (20 mg, 0.024 mmol) and **S9** (17 mg, 0.026 mmol) in 9:1 dichloromethane (0.9 mL):DMF (0.1 mL). The resulting yellow solution was stirred at 23 ºC for 1 h. At this point, LC-MS analysis showed full consumption of the acid starting material and formation of a new specie with the desired m/z. The reaction mixture was concentrated under reduced pressure to remove dichloromethane. The residue was diluted with 50% acetonitrile–water to a volume of 4.1 mL, and the solution was filtered through a 0.45 µM PTFE syringe filter. The filtrate was purified by reverse-phase HPLC (Waters XBridge C18 column 5 µm particle size 30 x 250 mm, 5–95% acetonitrile–water + 0.1% formic acid, 40 min, 20 mL/min) to afford the product as a yellow solid (18.7 mg, 56%).

3:2 mixture of rotamers.
^{1}H NMR (400 MHz, $CDCl_3$) δ 8.71 (s, 1H), 8.16 (s, 1H), 8.07 – 7.94 (m, 2H), 7.90 (d, *J* = 8.7 Hz, 1H), 7.72 (d, *J* = 9.2 Hz, 1H), 7.43 – 7.34 (m, 1H), 7.27 – 7.19 (m, 3H), 7.09 – 6.97 (m, 2H), 6.76 (d, *J* = 3.2 Hz, 1H), 6.55 (s, 1H), 5.42 (s, 1H), 5.18 (d, 3H), 5.12 – 5.04 (m, 2H), 4.70 (d, *J* = 4.7 Hz, 1H), 4.43 (d, *J* = 13.4 Hz, 1H), 4.07 – 3.92 (m, 2H), 3.92 – 3.80 (m, 1H), 3.80 – 3.66 (m, 2H), 3.61 (d, *J* = 10.1 Hz, 1H), 3.52 (s, 2H), 3.43 (s, 3H), 3.41 (s, 3H), 3.45 – 3.36 (m, 3H), 3.32 (s, 3H), 3.09 – 2.95 (m, 4H), 2.95 – 2.69 (m, 4H), 2.44 – 2.22 (m, 3H), 2.22 – 2.09 (m, 3H), 1.81 (d, *J* = 42.6 Hz, 8H), 1.69 – 1.60 (m, 6H),

1.60 – 1.32 (m, 8H), 1.11 – 1.04 (m, 2H), 1.02 (d, *J* = 6.3 Hz, 3H), 0.96 (d, *J* = 6.3 Hz, 3H), 0.86 (d, *J* = 7.3 Hz, 3H). Number of protons: Expected: 96, Calculated from peak integration: 90.

HRMS (ESI): Calcd for $(C_{75}H_{96}ClFN_6O_{15} + 2H)^{2+}$: 688.3381, Found: 688.3373

Synthesis of FK-Prostetin

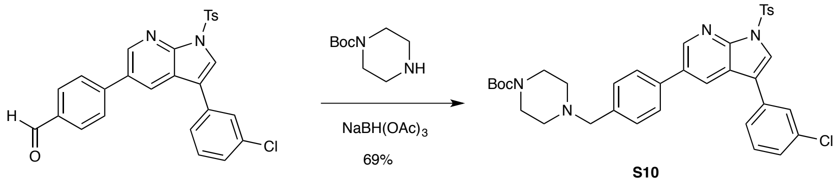

S10

tert-butyl 4-(4-(3-(3-chlorophenyl)-1-tosyl-1*H*-pyrrolo[2,3-*b*]pyridin-5-yl)benzyl)piperazine-1-carboxylate (**S10**)

N-Boc-Piperazine (229 mg, 1.23 mmol) and sodium triacetoxyborohydride (196 mg, 0.924 mmol) were added sequentially to a stirred solution of 4-[3-(3-chlorophenyl)-1-(p-tolylsulfonyl)pyrrolo[2,3-b]pyridin-5-yl]benzaldehyde[6] (300 mg, 0.6161 mmol) in DCM (3.0803 mL) at 23 ºC. The resulting mixture was stirred at 23 ºC and the reaction progress was monitored by LC-MS. In 18 h, LC-MS analysis showed full consumption of the aldehyde starting material. The reaction mixture was partitioned between saturated aqueous sodium bicarbonate solution (5 mL) and dichloromethane (5 mL). The layers were separated, and the aqueous layer was extracted with dichloromethane (2 x 5 mL). The combined organic layers were dried over sodium sulfate. The dried solution was filtered, and the filtrate was concentrated. The residue was purified by column chromatography (20–50% ethyl acetate–hexanes, 4-g RediSep(R) Rf column, Teledyne ISCO, Lincoln, NE) to afford the product as a yellow powder (279 mg, 69%).

^{1}H NMR (400 MHz, $CDCl_3$) δ 8.72 (d, *J* = 2.1 Hz, 1H), 8.23 – 8.13 (m, 3H), 7.94 (s, 1H), 7.62 (t, *J* = 1.8 Hz, 1H), 7.59 – 7.49 (m, 3H), 7.47 – 7.42 (m, 2H), 7.43 – 7.30 (m, 4H), 3.58 (s, 2H), 3.55 – 3.43 (m, 4H), 2.50 – 2.34 (m, 7H), 1.48 (s, 9H). Number of protons: Expected: 37, Calculated from peak integration: 37.

HRMS (ESI): Calcd for $(C_{36}H_{37}ClN_4O_4S + H)^+$: 657.2302, Found: 657.2278.

[6] Bos, P. H. *et al.* Development of MAP4 Kinase Inhibitors as Motor Neuron-Protecting Agents. *Cell Chem. Biol.* **26,** 1703-1715.e37 (2019).

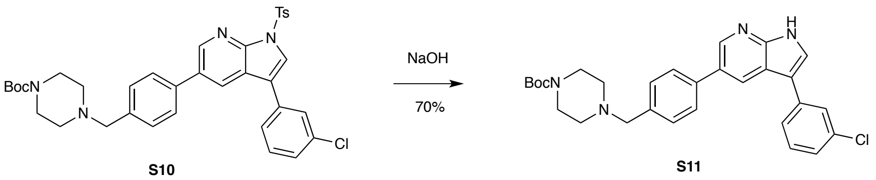

tert-butyl 4-(4-(3-(3-chlorophenyl)-1*H*-pyrrolo[2,3-*b*]pyridin-5-yl)benzyl)piperazine-1-carboxylate (**S11**)

S10 (279 mg, 0.43 mmol) was dissolved in a 1:1:1 mixture of acetone (2 mL):methanol (2 mL): 2 M aqueous NaOH (2 mL). The mixture was heated to 65 ºC. In 1 h, LC-MS analysis showed full deprotection of the tosyl group. The reaction mixture was partitioned between ethyl acetate (10 mL) and 1 N NaOH (10 mL). The aqueous layer was extracted with ethyl acetate (2 x 10 mL). The combined organic layers were dried over sodium sulfate, and the dried solution was concentrated. The residue was purified by column chromatography (20–50% ethyl acetate–hexanes, 4-g RediSep(R) Rf column, Teledyne ISCO, Lincoln, NE) to afford the product as a yellow powder (150 mg, 70%).

^{1}H NMR (400 MHz, $CDCl_3$) δ 9.43 (s, 1H), 8.64 (d, *J* = 2.1 Hz, 1H), 8.38 (d, *J* = 2.0 Hz, 1H), 7.70 – 7.55 (m, 5H), 7.50 – 7.37 (m, 3H), 7.32 (ddd, *J* = 8.0, 2.1, 1.1 Hz, 1H), 3.61 (s, 2H), 3.48 (t, *J* = 5.1 Hz, 4H), 2.46 (t, *J* = 5.1 Hz, 4H), 1.49 (s, 9H). Number of protons: Expected: 31, Calculated from peak integration: 31.
HRMS (ESI): Calcd for $(C_{29}H_{31}ClN_4O_2 + H)^+$: 503.2214, Found: 503.2233.

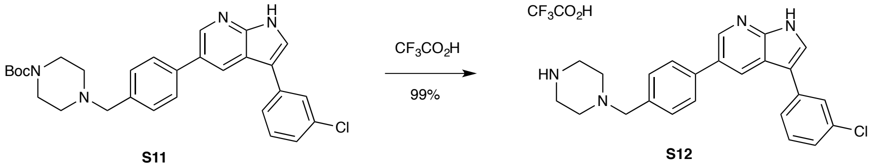

3-(3-chlorophenyl)-5-(4-(piperazin-1-ylmethyl)phenyl)-1*H*-pyrrolo[2,3-*b*]pyridine (**S12**)

S11 (150 mg, 0.299 mmol) was dissolved in 50% trifluoroacetic acid–dichloromethane (2.0 mL) and the resulting solution was allowed to stand at 23 ºC for 1 h. At this point, LC-MS analysis showed full consumption of the starting material and formation of the desired product. The reaction mixture was concentrated under reduced pressure and the residue was triturated with ether and dried under vacuum to afford the product as a yellow solid (151 mg, 99%).

^{1}H NMR (400 MHz, MeOD) δ 8.58 (d, *J* = 2.0 Hz, 1H), 8.54 (d, *J* = 2.1 Hz, 1H), 7.85 (s, 1H), 7.83 – 7.77 (m, 2H), 7.74 (t, *J* = 1.9 Hz, 1H), 7.69 (dt, *J* = 7.6, 1.3 Hz, 1H), 7.66 – 7.57 (m, 2H), 7.48 (t, *J* = 7.9 Hz, 1H), 7.33 (ddd, *J* = 8.0, 2.1, 1.0 Hz, 1H), 4.09 (s, 2H),

3.45 – 3.38 (m, 4H), 3.18 – 3.12 (m, 4H). Number of protons: Expected: 24, Calculated from peak integration: 21.
HRMS (ESI): Calcd for $(C_{24}H_{23}ClN_4 + H)^+$: 403.1689, Found: 403.1698.

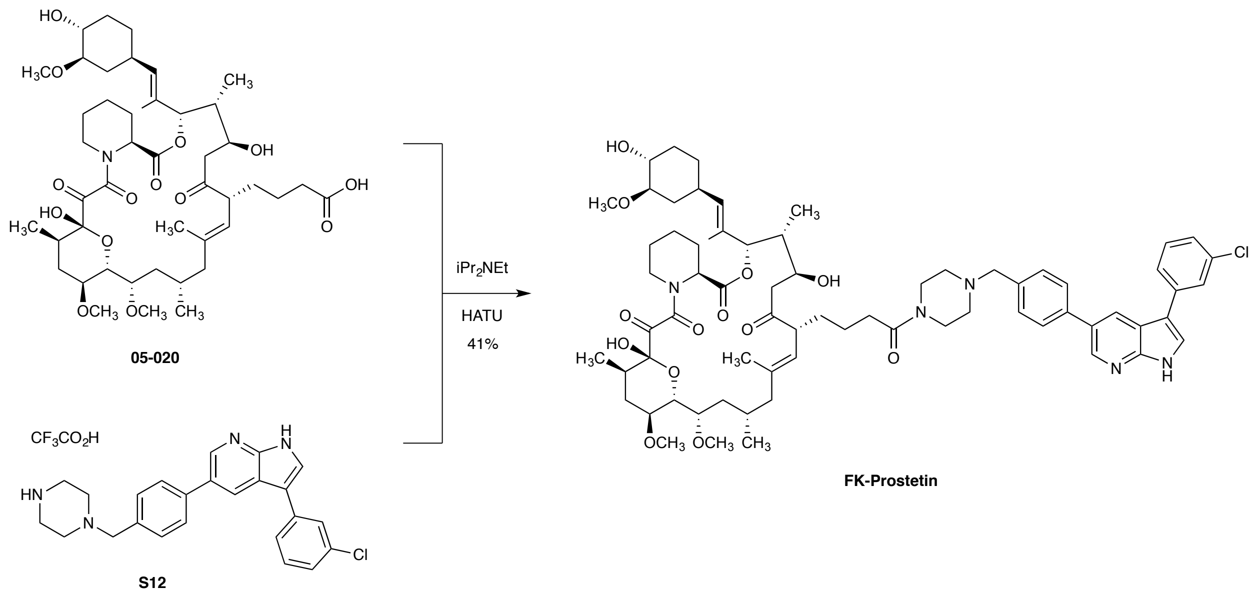

(1R,9S,12S,13R,14S,17R,18E,21S,23S,24R,25S,27R)-17-{4-[4-({4-[3-(3-chlorophenyl)-1H-pyrrolo[2,3-b]pyridin-5-yl]phenyl}methyl)piperazin-1-yl]-4-oxobutyl}-1,14-dihydroxy-12-[(1E)-1-[(1R,3R,4R)-4-hydroxy-3-methoxycyclohexyl]prop-1-en-2-yl]-23,25-dimethoxy-13,19,21,27-tetramethyl-11,28-dioxa-4-azatricyclo[22.3.1.0^{4,9}]octacos-18-ene-2,3,10,16-tetrone (**FK-Prostetin**)

N,*N*-Diisopropylethylamine (6.2 µL, 0.035 mmol) and HATU (4.5 mg, 0.012 mmol) were added sequentially to a stirred solution of **S12** (6.1 mg, 0.012 mmol) and **05-020** (10 mg, 0.012 mmol) in DMF (0.2 mL) at 23 ºC. The resulting mixture quickly turned yellow, and LC-MS analysis at 15 min showed full consumption of the FK506 acid starting material. The reaction mixture was diluted with 50% acetonitrile–water to a volume of 3.0 mL, and the solution was filtered through a 0.45 µM PTFE syringe filter. The filtrate was purified by reverse-phase HPLC (Waters XBridge C18 column 5 µm particle size 30 x 250 mm, 5–95% acetonitrile–water + 0.1% formic acid, 40 min, 20 mL/min) to afford the product as a white solid (5.9 mg, 41%).

HRMS (ESI): Calcd for $(C_{69}H_{92}ClN_5O_{13} + 2H)^{2+}$: 617.8268, Found: 617.8257.

Synthesis of RapaTAMRA

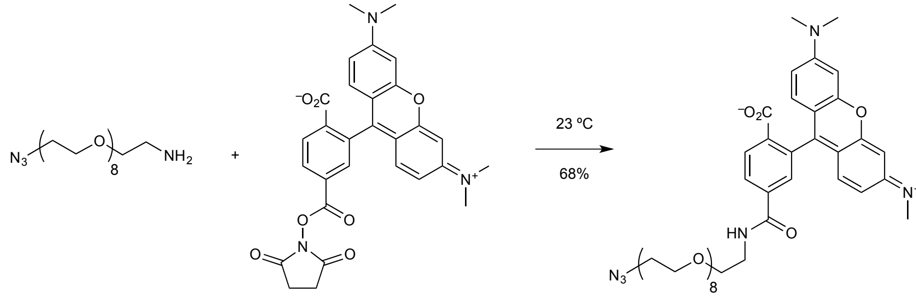

TAMRA-PEG8-azide

4-((26-azido-3,6,9,12,15,18,21,24-octaoxahexacosyl)carbamoyl)-2-(6-(dimethylamino)-3-(dimethyliminio)-3H-xanthen-9-yl)benzoate (**TAMRA-PEG8-azide**)

Azido-PEG8-Amine (43.6 mg, 0.100 mmol) was added to a solution of TAMRA 5-NHS ester (50 mg, 0.095 mmol) in DMF (0.47 mL) at 23 ºC. The mixture was stirred at 23 ºC for 30 min. The reaction mixture was concentrated to dryness under vacuum, and the residue was purified by column chromatography (0–20% methanol–dichloromethane, 4-g RediSep(R) Rf column, Teledyne ISCO, Lincoln, NE) to afford the product as a dark red solid (55 mg, 68%).

1H NMR (400 MHz, CDCl3) δ 8.40 (dd, J = 1.7, 0.8 Hz, 1H), 8.21 (dd, J = 8.0, 1.6 Hz, 1H), 7.23 (s, 1H), 6.60 (d, J = 8.9 Hz, 2H), 6.51 (d, J = 2.6 Hz, 2H), 6.41 (dd, J = 8.9, 2.6 Hz, 2H), 3.77 – 3.60 (m, 34H), 3.40 (t, J = 5.1 Hz, 2H), 3.01 (s, 12H). Number of protons: Expected: 58, Calculated from peak integration: 57.
HRMS (ESI): Calcd for $(C_{43}H_{58}N_6O_{12} + H)^+$: 851.4191, Found: 851.4188

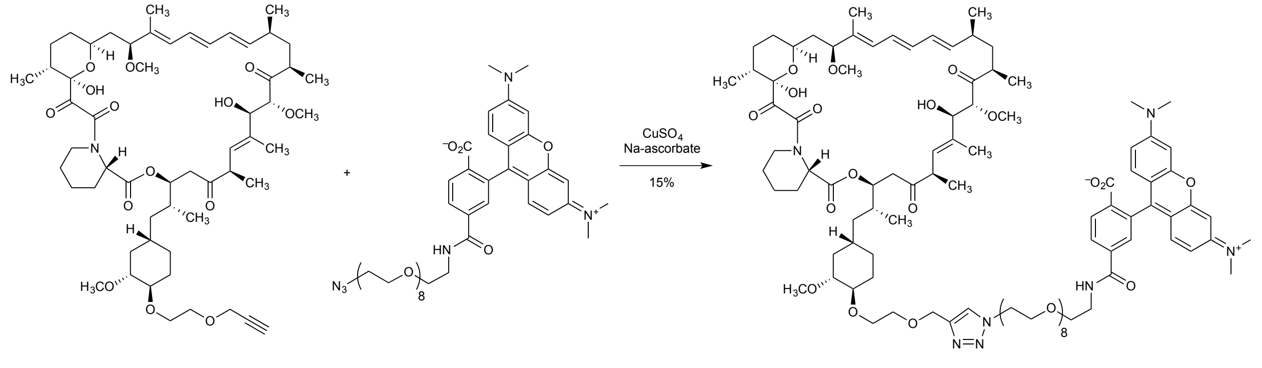

TAMRA-PEG8-azide

Rapa-TAMRA

4-[(26-{4-[(2-{[(1R,2R,4S)-4-[(2R)-2-[(1R,9S,12S,15R,16E,18R,19R,21R,23S,24E,26E,28E,30S,32S,35R)-1,18-dihydroxy-19,30-dimethoxy-15,17,21,23,29,35-hexamethyl-2,3,10,14,20-pentaoxo-11,36-dioxa-4-

azatricyclo[30.3.1.04,9]hexatriaconta-16,24,26,28-tetraen-12-yl]propyl]-2-methoxycyclohexyl]oxy}ethoxy)methyl]-1H-1,2,3-triazol-1-yl}-3,6,9,12,15,18,21,24-octaoxahexacosan-1-yl)carbamoyl]-2-[6-(dimethylamino)-3-(dimethyliminiumyl)-3H-xanthen-9-yl]benzoate (**RapaTAMRA**)

A one-dram vial was charged with *O40*-propargyloxyethyl-Rapamycin (20 mg, 0.020 mmol) (Rodrik-Outmezguine V.S., Okaniwa, M. *et al. Nature* **2016**, *534*, 272-276), TAMRA-PEG$_8$-azide (17.1 mg, 0.020 mmol), methanol (0.40 mL) and a magnetic stir bar. Freshly prepared aqueous solutions of copper(II) sulfate (0.10 M, 0.20 mL, 0.020 mmol) and sodium ascorbate (0.10 M, 0.40 mL, 0.040 mmol) were added sequentially to the reaction mixture. The reaction progress was monitored by LC-MS. In about 5 min, a brown precipitate appeared, which slowly dissolved as the reaction progressed. In 15 min, LC-MS analysis showed full consumption of the starting material and formation of a single species. 0.5 M aqueous Na$_2$-EDTA solution (100 μL) was added and the mixture was stirred for 10 min at 23 ºC. The resulting green mixture was diluted with 50% acetonitrile–water, and the solution was filtered through a 0.45 μM PTFE syringe filter. The filtrate was purified by reverse-phase HPLC (Waters XBridge C18 column 5 μm particle size 30 x 250 mm, 5–95% acetonitrile–water + 0.1% formic acid, 40 min, 20 mL/min) to afford the product as a purple solid (5.7 mg, 15.4%).

^{1}H NMR (400 MHz, $CDCl_3$, 4:1 mixture of rotamers, major rotamer is reported) δ 8.39 (s, 1H), 8.21 (d, *J* = 7.9 Hz, 1H), 7.74 (s, 1H), 7.21 (s, 1H), 6.59 (d, *J* = 8.8 Hz, 2H), 6.51 (d, *J* = 2.6 Hz, 2H), 6.41 (dd, *J* = 8.9, 2.6 Hz, 2H), 6.39 – 6.32 (m, 1H), 6.16 (dd, *J* = 14.8, 10.4 Hz, 1H), 5.98 (d, *J* = 10.3 Hz, 1H), 5.57 (dd, *J* = 15.2, 8.7 Hz, 1H), 5.44 (d, *J* = 10.0 Hz, 1H), 5.31 (d, *J* = 5.6 Hz, 1H), 5.22 – 5.16 (m, 1H), 4.84 – 4.80 (m, 1H), 4.71 (s, 2H), 4.54 (t, *J* = 5.1 Hz, 2H), 4.20 (d, *J* = 6.1 Hz, 1H), 3.88 (t, *J* = 5.2 Hz, 2H), 3.81 – 3.75 (m, 1H), 3.76 – 3.58 (m, 39H), 3.46 (s, 3H), 3.44 – 3.39 (m, 2H), 3.36 (s, 3H), 3.16 (s, 3H), 3.13 – 3.04 (m, 2H), 3.01 (s, 12H), 2.80 – 2.70 (m, 2H), 2.65 – 2.54 (m, 1H), 2.40 – 2.29 (m, 2H), 2.07 – 1.79 (m, 7H), 1.77 (s, 3H), 1.74 – 1.69 (m, 2H), 1.68 (s, 3H), 1.65 – 1.46 (m, 7H), 1.43 – 1.18 (m, 6H), 1.12 (d, *J* = 6.7 Hz, 3H), 1.07 (d, *J* = 6.6 Hz, 3H), 1.01 (d, *J* = 6.5 Hz, 3H), 0.97 (d, *J* = 6.6 Hz, 3H), 0.93 (d, *J* = 6.8 Hz, 3H), 0.88 – 0.84 (m, 1H), 0.77 – 0.70 (m, 1H). Number of protons: Expected: 143, Calculated from peak integration: 140. HRMS (ESI): Calcd for $(C_{99}H_{143}N_7O_{26} + 2H)^{2+}$: 924.0120, Found: 924.0126.

Supplementary Note 3. ^{1}H NMR and ^{13}C NMR Spectra of RapaBlock

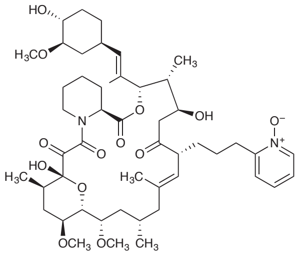

RapaBlock

NMR solvent: $CDCl_3$
Field Strength: 400 MHz (^{1}H)
100 MHz (^{13}C)

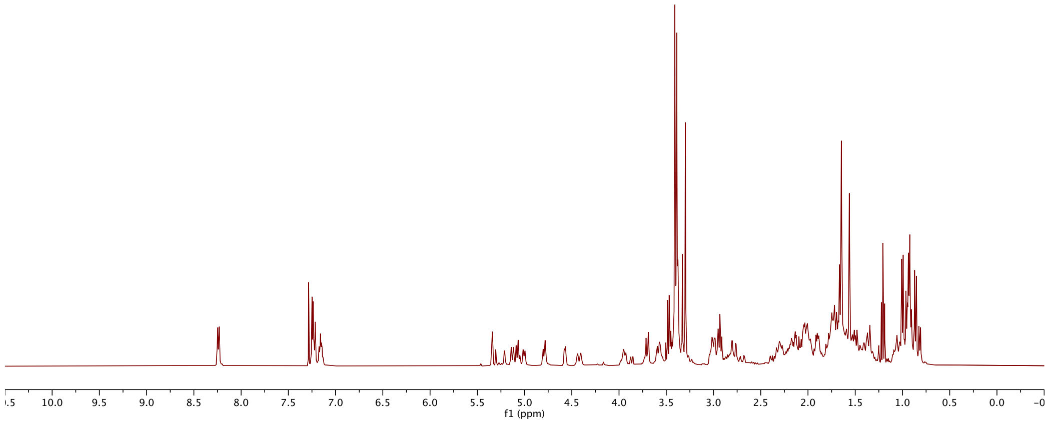

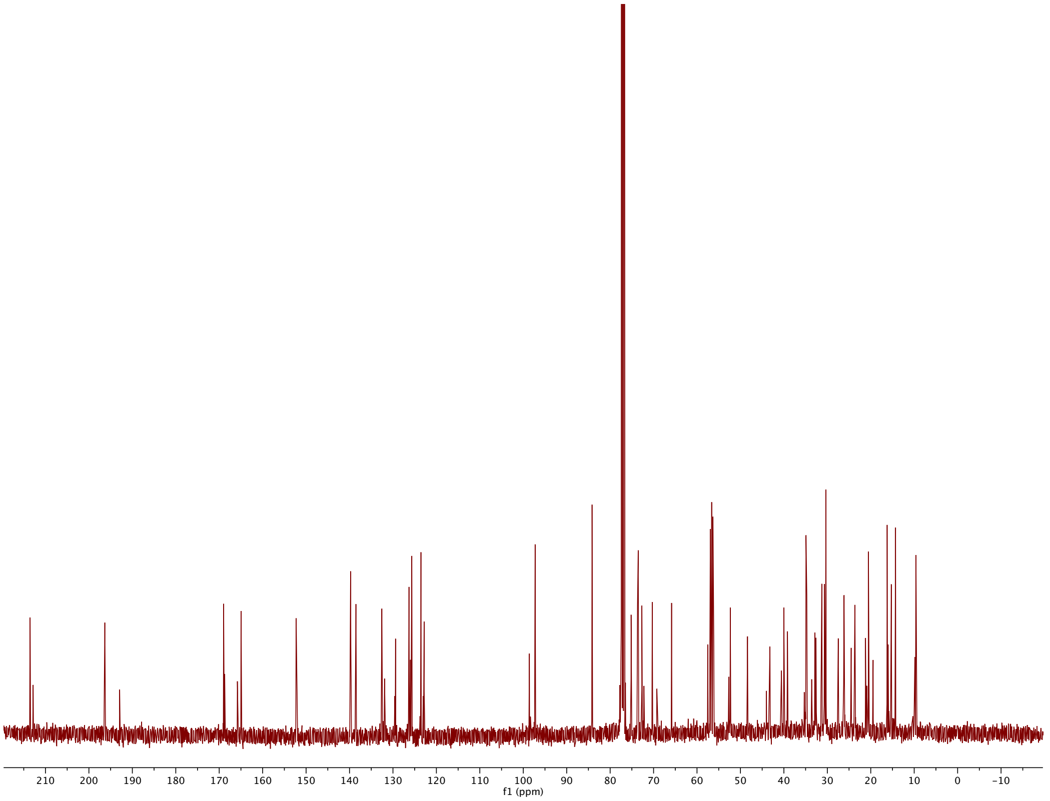

Supplement: Supplementary file 1 — This file contains Supplementary Figs. 1 and 2, Supplementary Table 2 (Supplementary Table 1 supplied separately) and Supplementary Notes 1–3 – see contents page for details. [file 41586_2022_5213_MOESM1_ESM.pdf]
