# Supplementary material for: Brain-restricted mTOR inhibition with binary pharmacology
Source: Nature. 2022 Sep 14;609(7928):822–8. doi: 10.1038/s41586-022-05213-y (PMC9492542; doi:10.1038/s41586-022-05213-y)
Supplement: Supplementary file 2 — Reporting Summary [file 41586_2022_5213_MOESM2_ESM.pdf]

nature research

Corresponding author(s): Kevan Shokat
Last updated by author(s): 06/08/2022

Reporting Summary

Nature Research wishes to improve the reproducibility of the work that we publish. This form provides structure for consistency and transparency in reporting. For further information on Nature Research policies, see our Editorial Policies and the Editorial Policy Checklist.

Statistics

For all statistical analyses, confirm that the following items are present in the figure legend, table legend, main text, or Methods section.

n/a	Confirmed	
☐	☒	The exact sample size (n) for each experimental group/condition, given as a discrete number and unit of measurement
☐	☒	A statement on whether measurements were taken from distinct samples or whether the same sample was measured repeatedly
☐	☒	The statistical test(s) used AND whether they are one- or two-sided<br>*Only common tests should be described solely by name; describe more complex techniques in the Methods section.*
☒	☐	A description of all covariates tested
☒	☐	A description of any assumptions or corrections, such as tests of normality and adjustment for multiple comparisons
☐	☒	A full description of the statistical parameters including central tendency (e.g. means) or other basic estimates (e.g. regression coefficient) AND variation (e.g. standard deviation) or associated estimates of uncertainty (e.g. confidence intervals)
☐	☒	For null hypothesis testing, the test statistic (e.g. F, t, r) with confidence intervals, effect sizes, degrees of freedom and P value noted<br>*Give P values as exact values whenever suitable.*
☒	☐	For Bayesian analysis, information on the choice of priors and Markov chain Monte Carlo settings
☒	☐	For hierarchical and complex designs, identification of the appropriate level for tests and full reporting of outcomes
☒	☐	Estimates of effect sizes (e.g. Cohen's d, Pearson's r), indicating how they were calculated

Our web collection on statistics for biologists contains articles on many of the points above.

Software and code

Policy information about availability of computer code

Data collection	Software used for data acquisition are listed below.<br>Flow cytometry: Thermo Fisher Scientific Attune NxT Software, 3.1.1243.0;<br>Plate reader assays: TECAN SparkControl 2.1;<br>Western Blot: Li-COR Odyssey 2.1.15<br>Western Blot (film based): SilverFast scan software (Version 6.4.2r9)
Data analysis	Flow cytometry: FlowJo 10.7.1<br>Plate reader assays: Prism 9.0<br>Western Blot: ImageJ 2.0.0-rc-69/1.52p<br>Calculation of cLogP and tPSA: ChemDraw Professional 16.0<br>Calculation of hydrogen bond donors and acceptors: MarvinSketch 20.21.0

For manuscripts utilizing custom algorithms or software that are central to the research but not yet described in published literature, software must be made available to editors and reviewers. We strongly encourage code deposition in a community repository (e.g. GitHub). See the Nature Research guidelines for submitting code & software for further information.

Data

Policy information about availability of data

All manuscripts must include a data availability statement. This statement should provide the following information, where applicable:

- Accession codes, unique identifiers, or web links for publicly available datasets
- A list of figures that have associated raw data
- A description of any restrictions on data availability

Unprocessed gel images for all immunoblots (figures 1d, 3a, 3d, 4a, extended data figures 3, 4c, 5, 7e, 7f, 8d) are provided in Supplementary Figure 1. Raw data for the kinase inhibition assays, cell growth assays and xenograft studies are available as source data files. All data that support the findings of this study are available from the corresponding author upon reasonable request.
Response to editor: The plate reader assay data in this manuscript are very small data sets using specialized assays (all described in the manuscript) which are not suitable for deposition in public data repositories. The manuscript does not contain new nucleic acid or protein structures. We are more than happy to provide the raw data upon request to the corresponding author.

Field-specific reporting

Please select the one below that is the best fit for your research. If you are not sure, read the appropriate sections before making your selection.

☒ Life sciences ☐ Behavioural & social sciences ☐ Ecological, evolutionary & environmental sciences

For a reference copy of the document with all sections, see nature.com/documents/nr-reporting-summary-flat.pdf

Life sciences study design

All studies must disclose on these points even when the disclosure is negative.

Sample size	Except for the kinase activity assays performed by thrid-party services, all in vitro experiments were performed in at least three replicates. For western blot analyses of mTOR signaling, three mice per treatment group were used. For U87MG xenograft studies, seven mice per treatment were used. For GBM43 xenograft studies, five mice per treatment were used. These numbers represent the maximum reasonable sample size that allowstimely administration of drugs and processing of tissue samples.
Data exclusions	No data was excluded from the analysis.
Replication	Kinase activity assays performed by third-party services (Figs 1b and 5c, Extended Figs 6c, 7b and 8b) were performed once with two technical replicates. Animal experiments were performed once with specified numbers of animals. All other experimental findings have been independently replicated at least twice (see figure captions for the number of replicates for individual experiments).
Randomization	For western blot analysis of mTOR signaling, mice were randomized into treatment groups. For U87MG tumor xenograft experiments, mice were sorted into four groups of equal mean bioluminescent signal at the beginning of the treatment(7 mice per group). For GBM43 tumor xenograft experiments, mice were sorted into four groups of equal mean bioluminescent signal at the beginning of the treatment(5 mice per group). Each biochemical experiment in this study is rationally designed and leads to a specific conclusion. Samples were not randomized for these experiments.
Blinding	Researchers were blinded from mice group assignment for imaging luciferase activity and measuring tumor burden. Solutions of RapaBlock were significantly more viscous than vehicle and solutions of RapaLink-1, and therefore full blinding in drug treatment was not attainable. Due to the short duration of treatment and limitation of personnel, animal experiments that analyze acute changes in mTOR signaling (Fig 4a and Extended Fig 3b) in this study were not blinded. Each biochemical experiment in this study is rationally designed and leads to a specific conclusion. Samples were not blinded for these experiments.

Reporting for specific materials, systems and methods

We require information from authors about some types of materials, experimental systems and methods used in many studies. Here, indicate whether each material, system or method listed is relevant to your study. If you are not sure if a list item applies to your research, read the appropriate section before selecting a response.

Materials & experimental systems

n/a	Involved in the study
☐	☒ Antibodies
☐	☒ Eukaryotic cell lines
☒	☐ Palaeontology and archaeology
☐	☒ Animals and other organisms
☒	☐ Human research participants
☒	☐ Clinical data
☒	☐ Dual use research of concern

Methods

n/a	Involved in the study
☒	☐ ChIP-seq
☐	☒ Flow cytometry
☒	☐ MRI-based neuroimaging

Antibodies

Antibodies used

A full list of antibodies used in provided in Supplementary Information.

Validation

P-Akt[S473] antibody (CST-4060) was validated by the manufacturer using wortmannin-treated and PDGF-treated samples as negative and positive controls, respectively. We independently validated this antibody using a pan-mTOR inhibitor MLN0128 in our experiments. https://www.cellsignal.com/products/primary-antibodies/phospho-akt-ser473-d9e-xp-rabbit-mab/4060
Akt antibody (CST-2920) was validated by the manufacturer using recombinant GST proteins and standard human cell lines. https://media.cellsignal.com/coa/2920/8/2920-lot-8-coa.pdf
P-S6 (S240/244) antibody (CST-5364) was validated by the manufacturer using insulin- or FBS-treated samples. We independently validated this antibody using the mTORC1 inhibitor rapamycin in our experiments. https://www.cellsignal.com/products/primary-antibodies/phospho-s6-ribosomal-protein-ser240-244-d68f8-xp-rabbit-mab/5364
P-S6 (S235/236) antibody (CST-4858) was validated by the manufacturer using lambda-phosphatase- and FBS-treated samples. We independently validated this antibody using the mTORC1 inhibitor rapamycin in our experiments. https://www.cellsignal.com/products/primary-antibodies/phospho-s6-ribosomal-protein-ser235-236-d57-2-2e-xp-rabbit-mab/4858
S6 antibody (CST-2217) was validated by the manufacturer using standard human and mouse cell lines and has been cited by 1130 publications. https://www.cellsignal.com/products/primary-antibodies/s6-ribosomal-protein-5g10-rabbit-mab/2217
P-4EBP1[T37/46] antibody (CST-2855) was validated by the manufacturer using insulin-treated samples. We independently validated this antibody using a pan-mTOR inhibitor MLN0128 in our experiments. https://www.cellsignal.com/products/primary-antibodies/phospho-4e-bp1-thr37-46-236b4-rabbit-mab/2855
4EBP1 antibody (CST-9644) was validated by the manufacturer using a cell line that contains mutation in the 4EBP1 gene that causes the production of a higher-molecular weight protein. https://www.cellsignal.com/products/primary-antibodies/4e-bp1-53h11-rabbit-mab/9644
FKBP12 antibody (abcam58072) was validated using recombinant FKBP12 proteins produced in-house.
Actin antibody (Proteintech, 60008-1-Ig) was validated by the manufacturer using a range of cell lines in Western blot and immunofluorescence, and has been cited by 1972 publiactions. https://www.ptglab.com/products/ACTB-Antibody-60008-1-Ig.htm
GAPDH antibody (Proteintech, 60004-1-Ig) was validated by the manufacturer using a range of cell lines in Western blot and immunofluorescence, and has been cited by 2971 publiactions. https://www.ptglab.com/products/GAPDH-Antibody-60004-1-Ig.htm
P-Tyr(4G10) antibody (EMD Millipore, 05-321) was validated by the manufacturer using EGF-treated A431 cell lysates. https://www.emdmillipore.com/US/en/product/Anti-Phosphotyrosine-Antibody-clone-4G10,MM_NF-05-321
COX IV antibody (CST-4850) was validated by the manufacturer using a range of cell lines. https://www.cellsignal.com/products/primary-antibodies/cox-iv-3e11-rabbit-mab/4850
P-ERK[T202/Y204] antibody (CST-9101) was validated by the manufacturer using recombinant phospho-MAPK proteins. https://www.cellsignal.com/products/primary-antibodies/phospho-p44-42-mapk-erk1-2-thr202-tyr204-antibody/9101
ERK antibody (CST-4695) was validated by the manufacturer using specific siRNA knockdowns and has been cited by 2827 publications. https://www.cellsignal.com/products/primary-antibodies/p44-42-mapk-erk1-2-137f5-rabbit-mab/4695
P-HER2[Y1221/1222] antibody (CST-2243) was validated by the manufacturer using EGF-stimulated cell lysates. https://www.cellsignal.com/products/primary-antibodies/phospho-her2-erbb2-tyr1221-1222-6b12-rabbit-mab/2243
P-HER3[Y1289] antibody (CST-2842) was validated by the manufacturer using human neuregulin-1-stimulated cell lysates. https://www.cellsignal.com/products/primary-antibodies/phospho-her3-erbb3-tyr1289-d1b5-rabbit-mab/2842
HER2 antibody (CST-4290) was validated by the manufacturer using authentic cell lines with known HER2 expression profile. https://www.cellsignal.com/products/primary-antibodies/her2-erbb2-d8f12-xp-rabbit-mab/4290
HER3 antibodies (CST-4754) was validated by the manufacturer using authentic cell lines with known HER3 expression profile. https://www.cellsignal.com/products/primary-antibodies/her3-erbb3-1b2e-rabbit-mab/4754
Goat anti-rabbit IgG-IRDye 800 was validated by the manufacturer: https://www.licor.com/documents/rfm2hw40wf33p06f3ndjrcorwi5usbft
Goat anti-mouse IgG-IRDye 680 was validated by the manufacturer: https://www.licor.com/documents/7bohf1sfzugccz22fh0um00cvz8ocizf

Eukaryotic cell lines

Policy information about cell lines

Cell line source(s)

MCF7 cells were obtained from ATCC (HTB-22) and maintained in 1:1 DMEM:F12 (Gibco) + 10% heat-inactivated fetal bovine serum (FBS, Axenia Biologix) supplemented with 4 mM L-glutamine, 100 U/mL penicillin and 100 U/mL streptomycin (Gibco). SK-BR-3 cells were obtained from ATCC (HTB-30) and maintained in McCoy's 5A (Gibco) + 10% heat-inactivated FBS supplemented with 2 mM L-glutamine, 100 U/mL penicillin and 100 U/mL streptomycin (Gibco). K562 CRISPRi cells were a

gift from Dr. Luke Gilbert and maintained in RPMI 1640 (Gibco) + 10% heat-inactivated FBS supplemented with 2 mM L-glutamine, 100 U/mL penicillin, 100 U/mL streptomycin (Gibco) and 0.1% Pluronic F-68 (Gibco). RAW264.7 cells were obtained from ATCC (TIB-71) and maintained in DMEM (Gibco) + 10% heat-inactivated FBS supplemented with 2 mM L-glutamine, 100 U/mL penicillin and 100 U/mL streptomycin (Gibco). Jurkat cells were obtained from ATCC (TIB-152) and maintained in RPMI 1640 (Gibco) + 10% heat-inactivated FBS supplemented with 2 mM L-glutamine, 100 U/mL penicillin and 100 U/mL streptomycin (Gibco). Jurkat-Lucia NFAT cells were obtained from InvivoGen and maintained in IMDM (Gibco) + 10% heat-inactivated FBS supplemented with 2 mM L-glutamine, 100 U/mL penicillin, 100 U/mL streptomycin (Gibco) and 100 µg/mL Zeocin (InvivoGen). HEK293T cells were obtained from UCSF Cell Culture Facility and maintained in 1:1 DMEM:F12 (Gibco) + 10% heat-inactivated fetal bovine serum (FBS, Axenia Biologix) supplemented with 4 mM L-glutamine, 100 U/mL penicillin and 100 U/mL streptomycin (Gibco).

Authentication

Cell lines were obtained from vendors noted above and all cell experiments were performed within 15 passages from the initial ATCC stock. Cell morphology were regularly inspected. Cell lines from ATCC were STR profiled by the manufacturer. Jurkat-Lucia NFAT cells is a commercial cell line developed by InvivoGen and was authenticated by the manufacturer. Cell lines from UCSF Cell Culture Facility were STR profiled by the UCSF Cell Culture Facility. No further authentications were performed after the purchase.

Mycoplasma contamination

All cell lines were tested mycoplasma negative using MycoAlert™ Mycoplasma Detection Kit (Lonza).

Commonly misidentified lines
(See ICLAC register)

No commonly misidentified cell lines were used in the study.

Animals and other organisms

Policy information about studies involving animals; ARRIVE guidelines recommended for reporting animal research

Laboratory animals

4-6 week-old female athymic BALB/Cnu/nu athymic nude mice were used in vivo experiments. All mice at the Helen Diller Cancer center UCSF are housed in individually ventilated microisolator cages. The housing racks have automatic water that is sterilized through filtration, reverse osmosis, and UV light exposure. All mice are fed a standard diet that is pre-irradiated. The light/dark cycle is 12 light/12 dark. Temperature is maintained in the range of 68-74 degrees Fahrenheit. Humidity is maintained at 30-70%.

Wild animals

No wild animals were used in the study.

Field-collected samples

No field-collected samples were used in the study.

Ethics oversight

All animal experiments were conducted using protocols approved by University of California, San Francisco’s Institutional Animal Care and Use Committee (IACUC).

Note that full information on the approval of the study protocol must also be provided in the manuscript.

Flow Cytometry

Plots

Confirm that:

☒ The axis labels state the marker and fluorochrome used (e.g. CD4-FITC).

☒ The axis scales are clearly visible. Include numbers along axes only for bottom left plot of group (a 'group' is an analysis of identical markers).

☒ All plots are contour plots with outliers or pseudocolor plots.

☒ A numerical value for number of cells or percentage (with statistics) is provided.

Methodology

Sample preparation

Untransduced (No virus or sgRNA-) and sgRNA expression vector-transduced (sgRNA+) K562 CRISPRi cells were mixed in a 1:1 ratio, plated in 48-well plates, and incubated overnight. Cell mixtures were treated with the indicated TAMRA-linked compounds (300 µL final volume) and incubated for 24 h. Plates were placed over ice and 200 µL of each compound-cell mixture was transferred to a 96-well U-bottom plate. Cells were pelleted at 500g for 5 min, and subsequently washed 2× with ice-cold FACS buffer (PBS + 1% BSA + 0.1% NaN3). Cells were resuspended in 200 µL of ice-cold FACS buffer and assessed using an Attune NxT (Thermo Fisher Scientific). Relative uptake of TAMRA-linked compounds was determined by comparing TAMRA fluorescence (561 nm excitation laser, 585/16 emission filter) between sgRNA- and sgRNA+ cells within each well.

Instrument

Thermo Fisher Scientific Attune NxT

Software

FlowJo 10.7.1

Cell population abundance

50 µL of cell mixture was assayed (typically ≥ 10,000 total events) on an Attune NxT (Thermo Fisher Scientific). The gating strategy proceeded as follows: 1) A forward scatter area (FSC-A) vs. side scatter area (SSC-A) gate was used to exclude debris (~90% gated), 2) A FSC-A vs. forward scatter height (FSC-H) gate was used to exclude doublets (~90% gated), 3) A VL1 height (VL1-H, 405 nm excitation laser, 440/50 emission filter) vs. YL1 height (YL-H, 561 nm excitation laser, 585/16 emission filter) gate was used to assess sgRNA expression and TAMRA-linked compound uptake respectively

Gating strategy

Gating strategy is provided in Supplemental Fig. 2. This gating strategy is used for data shown in Extended Data Figure 1c and 6b.
Response to editor: this gating strategy is a graphical demonstration and cannot be directly placed in the reporting summary.

☒ Tick this box to confirm that a figure exemplifying the gating strategy is provided in the Supplementary Information.
